# Differential gene expression and gene ontologies associated with increasing water-stress in leaf and root transcriptomes of perennial ryegrass (*Lolium perenne*)

**DOI:** 10.1371/journal.pone.0220518

**Published:** 2019-07-30

**Authors:** Albert Fradera-Sola, Ann Thomas, Dagmara Gasior, John Harper, Matthew Hegarty, Ian Armstead, Narcis Fernandez-Fuentes

**Affiliations:** 1 Quantitative Proteomics, Institute of Molecular Biology (IMB), Mainz, Germany; 2 Institute of Biological, Environmental and Rural Sciences, Aberystwyth University, Aberystwyth, United Kingdom; Louisiana State University, UNITED STATES

## Abstract

Perennial ryegrass (*Lolium perenne*) is a forage and amenity grass species widely cultivated in temperate regions worldwide. As such, perennial ryegrass populations are exposed to a range of environmental conditions and stresses on a seasonal basis and from year to year. One source of potential stress is limitation on water availability. The ability of these perennial grasses to be able to withstand and recover after periods of water limitation or drought can be a key component of grassland performance. Thus, we were interested in looking at changes in patterns of gene expression associated with increasing water stress. Clones of a single genotype of perennial ryegrass were grown under non-flowering growth room conditions in vermiculite supplemented with nutrient solution. Leaf and root tissue was sampled at 4 times in quadruplicate relating to estimated water contents of 35%, 15%, 5% and 1%. RNA was extracted and RNAseq used to generate transcriptome profiles at each sampling point. Transcriptomes were assembled using the published reference genome sequence and differential gene expression analysed using 3 different programmes, DESeq2, edgeR and limma (with the voom transformation), individually and in combination, deriving Early, Middle and Late stage comparisons. Identified differentially expressed genes were then associated with enriched GO terms using BLAST2GO. For the leaf, up-regulated differentially expressed genes were strongly associated with GO terms only during the Early stage and the majority of GO terms were associated with only down-regulated genes at the Middle or Late stages. For the roots, few differentially expressed genes were identified at either Early or Middle stages. Only one replicate at 1% estimated water content produced high quality data for the root, however, this indicated a high level of differential expression. Again the majority of enriched GO terms were associated with down-regulated genes. The performance of the different analysis programmes and the annotations associated with identified differentially expressed genes is discussed.

## Introduction

Perennial ryegrass (*Lolium perenne*) is a widely cultivated perennial forage and amenity grass in temperate areas worldwide and, as such, is either the major component or is a key constituent of many perennial pasture types. As a consequence of this wide distribution, perennial ryegrasses are exposed to, and have to respond to, a variety of environmental stresses, some seasonal, but others with increasingly unpredictable onsets as a consequence of changing climates. Reduced water availability, as a result of low rainfall or irrigation, is one such environmental stress which can have significant effects on the establishment and persistence of grasslands. This, in turn, can influence both agricultural productivity and the economics of farming as well as having broader impacts on the environment and grassland sustainability [1, 2]. Thus, there is a driver to understand perennial ryegrasses responses to water deficit at both whole plant and molecular levels and to integrate this information into the process of developing new perennial ryegrasses which have the ability to withstand and recover from periods of drought.

Numerous studies have reported relative changes in transcriptome profiles of model and crop plants in response to water-stress, often with a focus on the identification of differentially expressed genes (DEGs) distinguishing drought tolerant from susceptible genotypes. Such studies frequently indicate the likely roles and interplay of phytohormone signalling, specific transcription factors, receptor kinases, transporter and stress-response proteins (among others) in implementing the metabolic and physiological programmes required to deal with the onset of water-stress (e.g., [3–17]). An aspect of the majority of these studies is that, for obvious practical reasons, they have isolated, analysed and compared transcriptomes from the aerial parts of the plant. While this is experimentally more tractable, this has led to a relative lack of knowledge of the gene expression responses of roots to the progression of water stress. To address this, experimental systems have been developed which allow access to growing roots during the imposition of water stress, both for transcriptomic and physiological analyses. These include hydroponic systems, with osmotic stress being imposed using high molecular weight polyethylene glycol (PEG) [18–21] and the use of other suitable soil, sand [4, 13, 22, 23] and non-soil based hydrated media such as vermiculite [24–28], from which roots can be isolated relatively rapidly while minimising mechanical damage, at least for simpler root systems.

In addition to developing suitable experimental systems, there are also a number of contrasting analytical methods, implemented in different computational approaches, which can be taken to the identification of differential gene expression. A number of studies have compared the performance of different packages for identifying differentially expressed genes (DEGs) from RNAseq datasets [29–32] and made recommendations, taking into account factors such the structure of the overall analysis pipeline and the numbers of replicates. For instance, Schurch et al. [30] identified 5 programs that show a high level of inter-software consistency (DESeq [33], DESeq2 [34], EBSeq [35], edgeR [36], limma [37]) and recommended the use of DESeq2 for experiments with fewer replicates per condition. Costa-Silva et al. [29] identified that combining different packages can refine sets of DEGs to make them more concordant with qRT-PCR data. The identification of numbers of DEGs within experiments is often given a broader biological relevance through the use of Gene Ontologies (GO). These allow the results of individual experiments to be contextualised within the body of existing experimental evidence to identify consistent patterns of gene expression associated with defined biological processes, molecular functions and cellular compartments. To enable this, a number of open-access and other resources have become available which allow for the assignment of functions or putative functions to genes or gene models through the use of sequence similarity searches in combination with direct or indirect experimental evidences (e.g., BLAST2GO [38]; The Gene Ontology Consortium[39] QuickGO [40])

Our overall aim in this study has been to develop a greater understanding of changes in gene expression patterns in leaves and roots of perennial ryegrass and, particularly, how these reflect underlying biological processes as indicated through GO terms. Additionally, we were interested in whether derived conclusions might be influenced by the use of different softwares. To approach this we have: A) generated leaf and root transcriptomes from a single genotype of perennial ryegrass at four points during a time course reflecting increasing water stress, B) compared changes in DEG expression patterns using 3 commonly used programmes, DESeq2, limma with voom transformation (hereafter referred to as limma-voom) and edgeR and C) identified enriched GO terms associated with groups of up- and down-regulated DEGs at the different time points, comparing DEGs identified by: i) the 3 programmes individually, ii) all the programmes, and iii) any of the programmes. The results are discussed in the contexts of the different analytical approaches and the biological responses to increasing water-stress.

## Materials and methods

### Plant material and drought treatments

A single, largely homozygous, genotype of perennial ryegrass (p226/135/16), derived from an inbred line was used for the RNAseq experiments. This is the same genotype used as the subject of the first published perennial ryegrass reference genome assembly [41]. All replicates were derived by clonal propagation (tillering) from this single genotype.

Throughout the experiment, plants were maintained in a 20°C growth room with an 8 hour photoperiod. Perennial ryegrass is a long-day plant and these conditions maintained the replicates in the vegetative growth stage (i.e., no induction to flowering).

Sixteen single tillers were taken from compost grown clonal replicates of p226/135/16, rinsed of compost, and transferred to containers of water until they showed new root growth, after about 6 days on average. At this point they were transferred to 90 mm pots containing vermiculite (graded for horticultural use, 2–5 mm) to establish and were watered with Hoagland’s solution [42] twice a week. Once established, between 15 and 21 days after tillering, watering was stopped and water content was estimated (estimated water content; EWC) using a moisture meter HH2 Delta-T meter (AT Delta-T devices, Cambridge, UK). At each estimation 3 different moisture readings were taken and averaged. Leaves and roots were sampled at 35% (full watering, day 0) and when 15% (day 5 –no visible wilting), 5% (day 12 –visibly starting to wilt) and 1% (day 20 –marked wilting) EWC levels were reached (see S1 Fig for images of the replicates at the different EWC stages). Leaf samples were cut and flash frozen in liquid nitrogen and stored at -80°C. The roots were briefly washed with distilled water to remove the growing medium and then blotted dry prior to storage at -80°C. Four different clones were sampled at each EWC-point, deriving 4 biological replicates for each stage.

### RNA extraction and sequencing

Total RNA was extracted from leaf material using the Trizol method (Sigma-Aldrich, Poole, UK) and quantified using Qubit fluorescence spectrophotometry. 1ug of total RNA was used per sample for library construction according to the Illumina TruSeq Stranded mRNA Library Preparation Kit protocol. Samples were indexed such that 24 samples could be multiplexed per lane of a HiSeq2500 platform (2x126bp format). Samples were run across two high-output flowcells and reads demultiplexed using the bcl2fastq script.

### RNAseq processing and quality control and mapping

Prior to mapping, raw reads were processed using Trimmomatic v.0.33 [43] to remove adapters using the following parameters (optimized after several run tests): ILLUMINACLIP:TruSeq3-PE-2.fa LEADING:15 SLIDINGWINDOW:4:15 MINLEN:30 HEADCROP:12, and the quality of resulting trimmed and cleaned reads was assessed using FastQC v.0.11 [44]. Reads were then mapped to perennial ryegrass (p226/135/16) reference genome assembly [41] using the splice-aware mapper hisat2 v2.0.0 [45] with default parameters to generate the bam files.

#### Pre-processing and quantification of transcripts

Prior to analysing the count matrices a pre-processing filtering was performed to remove potential artefacts and assess the quality of the replicates as described elsewhere [46, 47]. Count matrices were derived from bam files above using the *GenomicFeatures and GenomicAlignments* R libraries. Transcripts with a count lower than in any samples were discarded. We applied the regularized logarithm transformation (rlog) as implemented in the DESeq2 package to decrease the variance among gene expression values as proposed by Love et al. [34] and then calculated a distance matrix between samples and performed a principle component analysis (PCA) to quantify experimental covariates and batch effects among samples and replicates [48]. The R scripts implementing the pre-processing and generation of count matrices are available in S1 Methods.

#### Estimating the completeness of transcriptomes

The transcriptome in each sample was assessed for its completeness as a measure of quality of the sequencing. Clean reads were mapped to the reference genome [41] using the splice-aware mapper Hisat2 v.2.0.0 [45] with default parameters and special options:—dta (required for downstream processing with StringTie)—phred33 (required to handle Illumina reads). Subsequently transcripts were assembled and merged using StringTie v1.1.0 [49] using default parameters. The completeness of each transcriptome was assessed using BUSCO [50] on the early_release plantdb set, composed of 1440 core genes.

### Identification of DEGs using DESeq2, edgeR and limma-voom and deriving Jury 1 and Jury 3 DEG categories

Three different, count-based, methods were used to identify DEGs: DESeq2 [34], edgeR [36], and limma [51] with voom transformation [37]—limma-voom. In the case of DESeq2 the counts where transformed using the Relative Log Expression function [33][30] while in the case of edgeR and limma-voom the trimmed mean M-values were used [52]. Two scenarios were considered for the identification of DEGs referred to as *against reference* (AR) and *time-course* (TC). In the case of AR, the RNA samples taken at 35% EWC were considered the reference or control, hence samples at 15%, 5% and 1% EWC were compared against the 35% sample to generate the Early, Middle and Late comparisons, respectively. In the case of TC analyses the comparisons performed were: 35 vs 15% (Early), 15 vs 5% (Middle) and 5 vs 1% (Late), i.e., each sampling point was compared to the previous sampling point, the latter being considered to be the reference. Only genes with a 2-fold change in expression level and a 5% false discovery rate (FDR) were considered as significant (R scripts code are available in S2–S4 Methods for DESeq2, edgeR and limma-voom, respectively).

For each of the AR and TC categories, we further defined the Jury 1 (J1) and Jury 3 (J3) classes. For J1 we considered any gene as differentially expressed if identified as significant by any of the methods used, i.e., a gene with ≥ 2-fold change in expression level and ≤ 5% FDR in any of the following: DESeq2, edgeR or limma-voom. In the case of J3, only those genes identified as significant by all three softwares were considered.

#### Functional annotation of DEGs

The reference genome was functionally re-annotated using Blast2GO 5.25 (Pro) [38] as a prior step before computing GO term enrichments. The functional annotation was done as follows: BLAST searches were performed on the nr database (release Jan 2017) using BLASTx command from ncbi-blast-2.2.28+ release [53] at an e-value cut-off of 0.000001 and selecting the top 20 hits. InterPro searches were performed using InterProScan v.5.18–57 [54] on TIGRFAM [55], PFAM [56], SMART [57], PANTHER [58], Gene3d [59] and PIRSF [60] databases. The annotation table is available in S1 Table.

#### Partitioning of DEGs between expression categories

RNAseq was performed on samples extracted at the four EWC stages, 35%, 15%, 5% and 1% and DEGs were identified using DESeq2, edgeR and limma-voom and the combination categories J1 and J3 (as described above). DEGs identified in the Early, Middle and Late AR and TC comparisons were classified as to whether they were up- or down-regulated relative to the 35% EWC stage (AR) or the previous EWC stage (TC). This meant that every gene model could be assigned to one of 27 expression categories for AR and TC comparisons, describing whether it was significantly up-regulated, down-regulated or showed no significant change in expression levels at Early, Middle and Late comparison stages. For example, a gene model identified as up-regulated at the Early AR comparison, down-regulated at the Middle AR comparison and showing no significant change at the Late AR comparison is described as belonging to an expression category using the notation AR-*up_down_ns*.

### Identification of enriched GO terms

After placing each gene model showing significant differential expression in at least one of the Early, Middle or Late comparisons in one of the AR and/or TC expression categories, significantly enriched GO terms were identified for each expression category using the Enrichment Analysis (Fisher’s Exact Test) module in BLAST2GO with the FDR set at 0.05, and the GO terms associated with the annotated perennial ryegrass genome as the reference set. Subsequently, the gene models contributing to the significant enrichment of the individual GO terms associated with each expression category were extracted from the BLAST2GO output files.

### Kyoto Encyclopedia of Genes and Genomes (KEGG) analysis

Enzyme activity codes were assigned to subsets of gene models using the BLAST2GO KEGG [61] interface. Where the numbers of up- and down-regulated DEGs assigned to particular enzyme codes within certain expression categories were compared, significance was estimated using Fisher’s Exact Test.

### Additional functional information on proteins

As described in the Results and Discussion in relation to particular DEGs, additional functional information was inferred from annotations using the Uniprot database[62].

## Results

### Pre-processing, mapping, and quality of sequencing and replicates

The RNA libraries were sequenced in a single batch yielding an average of around 15 and 10M reads per sample for shoot and root tissue respectively (Tables 1 and S2). The number of reads across replicates was fairly consistent with the exception of root tissues at 5 and 1% EWC where there were large differences among replicates. As a consequence root sample replicate 3 from 5% EWC and replicates 1, 3 and 4 from 1% EWC were not considered when computing summary statistics or DEGs (see below). Thus, the 1% EWC root sample was unreplicated. After removing adaptors, low quality and non-paired reads, the number of reads dropped by around 13% on average (Tables 1 and S2). The completeness of the transcriptome was estimated using BUSCO (Table 2); the average number of core genes represented in the transcriptome, including complete and partial genes, was c. 93% for the leaf samples and 88% for the root samples (excluding the low quality root replicates).

**Table 1:** Sequencing and mapping statistics. The total number and number of paired reads shown as the aggregate value across all four replicates at different estimated water contents. Alignment rates are shown as the range among replicates. The mapped reads (bam files) were submitted to the European Nucleotide Archive (ENA) repository under study accession number PRJEB31812.

	Estimated water content			
**Leaf**	**35%**	**15%**	**5%**	**1%**
Total reads	71830102	61024525	86519927	67957287
Paired Reads	52433026	45412817	60453862	46278550
Alignment rate (%)	95–96	94–95	95–95	87–95
**Root**	**35%**	**15%**	**5%**^1^	**1%**^2^
Total reads	31810877	54311559	27220469	11123223
Paired Reads	26154592	43984322	21061835	8504146
Alignment rate (%)	77–91	91–95	95–96	92

^1^Average/range of 3 biological replicates

^2^One biological replicate

**Table 2:** Transcriptome completeness. The % completeness of the different transcriptomes at the different estimated water contents (EWCs) as estimated using the BUSCO core gene set.

Tissue	EWC (%)	BUSCO		
		Complete	Partial	Missing
Leaf	35	84.41	7.11	8.47
	15	84.41	10.67	7.43
	5	84.73	6.90	8.37
	1	85.04	7.32	7.64
Root	35	81.90	8.05	10.04
	15	84.52	7.32	8.16
	5^1^	79.81	7.64	12.55
	1^2^	71.15	9.31	19.54

^1^Average of 3 biological replicates

^2^One biological replicate

**Fig 1:**
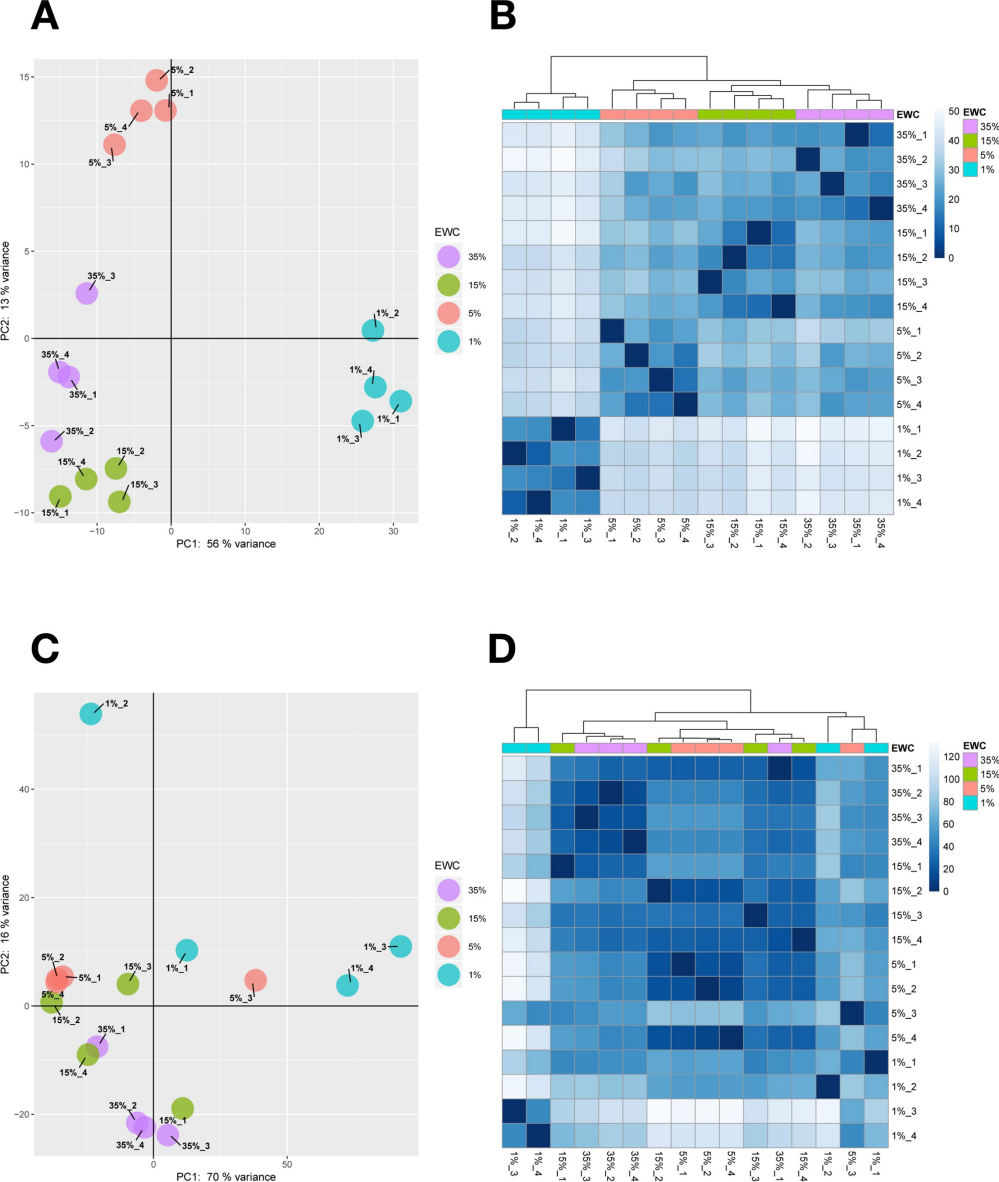
Variability among replicates from leaf and root transcriptomes. Panels A and C show PCA and heat map plots for shoot and root tissues respectively and Panels B and D portrays the heat maps computed with Euclidian distance within samples for shoot and root tissues respectively. Samples at EWC 35, 15, 5, and 1% are coloured in purple, green, red and cyan respectively.

The variability among sampling point replicates are shown in Figs 1 and S2 and S3. For the shoot samples (S2 Fig), the distributions of rlog values were homogeneous across the 16 samples (four EWC points and four replicates) indicating consistent biological replication. As referred to above, there was more variation among the root samples at 5% and 1% EWC (S3 Fig), leading to the omission of some of the replicates from the DEG analysis. The analysis of variability among replicates in the form of PCA and heat maps using the count matrices showed that shoot replicates within each EWC sampling point clustered together and the first two components of the PCA accounts for up to 69% of the overall variability (Fig 1). Thus, change in EWC as the experiment progressed was the main driver explaining the variability between EWC sampling points. This analysis also indicated that samples taken at 1% EWC were the most dissimilar, and that 35% and 15% EWC sampling points were the most similar. The PCA and heat map plots generated from the root data, by contrast, indicated a higher degree of variability between replicates and a greater degree of overlap between the 35% and 15% EWC sampling points (Fig 1). Three of the 4 replicates at 5% EWC were very consistent, though the fourth was an outlier that was not used. The single replicate retained from the 1% EWC sampling point was distinctly separated from the other retained sampling point replicates, particularly on the basis of the second principle component.

### Differentially expressed gene models

A summary of the numbers of DEGs detected at the different comparison stages and by the 3 analysis programmes is given in Table 3. As a trend, the number of DEGs detected at each stage comparison increased as the experiment proceeded in both AR and TC comparisons, varying between 642 DEGs (J3, Early AR/TC) to 3762 (J1, Late, AR). The pattern of DEG identification in the root transcriptomes contrasted markedly with the leaf transcriptomes particularly in reference to the performance of the different analysis programmes. Only 5 DEGs were called in the Early comparison stage, all by DESeq2; in the Middle AR comparison stage a total of 844 DEGs were called, but again only by DESeq2 (with the exception of 1 DEG also called by limma-voom). For the Late comparison stage, as described earlier, 3 of the 4 replicates generated low or very low alignments with the reference genome and only 1 of the replicates was useable. However, from this one sample, between 3380 and 3673 DEGs were identified by DESeq2, edgeR and limma-voom which indicates substantial differential expression as the roots approached this end stage. Thus, major changes in differential expression in the leaf transcriptome occurred over the duration of the experiment, whereas major changes in differential expression in the root transcriptome did not occur in the Early and Middle comparison stages and were detected only during the most severe water stress (bearing in mind this conclusion is based on an unreplicated result for the Late stage comparison).

**Table 3:** The number of significant differentially expressed genes detected by each analysis method for each expression comparison.

		Analysis method				
	Comparison	J1	J3	DESeq2	edgeR	limma-voom
**Leaf**	**AR/TC Early**	1050	642	934	754	774
	**AR Middle**	1838	1195	1564	1450	1494
	**TC Middle**	1860	1290	1622	1502	1553
	**AR Late**	3762	3024	3311	3352	3497
	**TC Late**	2701	2065	2397	2334	2403
**Root**	**AR/TC Early**	5	0	5	0	0
	**AR Middle**	844	0	844	0	1
	**TC Middle**	186	1	186	4	1
	**AR Late**	3932	2903	3673	3344	3380
	**TC Late**	2757	1787	2622	2196	2189

### Comparison of individual DEGs identified at different stages and through different analysis methods

Table 4 indicates the frequencies with which individual DEGs were called across the different combinations of methods. For the leaf transcriptome, between 61% and 80% of the DEGs included within J1 were called by all of the methods (J3). Between 3 and 5% of the DEGs included in J1 were called by combinations of just 2 of the programmes and between 3 and 9% were called by only one of the programmes–primarily DESeq2 or limma-voom. The root transcriptome again contrasted with the leaf transcriptome. As described earlier, few DEGs were called by any of the programmes in the Early and Middle stages and by far the majority of those that were, were called uniquely by DESeq2. For the single sample Late stage comparisons, in which a total of 3932 and 2757 DEGs were included in J1 for the AR and TC comparisons, respectively, 74% (AR) and 65% (TC) were called by all of the programmes (J3). These levels are comparable to those obtained for the leaf transcriptomes.

**Table 4:** The total number of differentially expressed genes (J1) identified at each comparison stage and the percentage of these identified by all possible combinations of the analysis methods DESeq2 (D), edgeR (E) and limma-voom (L).

		Combinations of analysis methods							
		J1	D,E,L (J3)	D,E	D,L	E,L	D	E	L
	Comparison	n	%J1	%J1	%J1	%J1	%J1	%J1	%J1
**Leaf**	**AR/TC Early**	1050	61	8	2	2	18	1	8
	**AR Middle**	1838	65	9	3	3	8	2	10
	**AR Late**	3762	80	4	2	4	2	1	7
	**TC Middle**	1860	69	9	2	2	7	1	10
	**TC Late**	2701	76	7	2	3	4	1	8
**Root**	**AR/TC Early**	5	0	0	0	0	100	0	0
	**AR Middle**	844	0	0	0.1	0	100	0	0
	**AR Late**	3932	74	7	3	3	10	1	3
	**TC Middle**	186	1	2	0	0	98	0	0
	**TC Late**	2757	65	10	4	2	17	1	2

### DEG expression patterns across early, middle and late AR and TC stage comparisons and contribution to GO category enrichment

For the shoot transcriptomes, when the Early, Middle and Late comparisons were considered in sequence and in combination with whether a particular gene was significantly up- or down-regulated or showed no-significant relative change in expression level, 27 different categories could be derived for both AR and TC. Table 5 describes the distribution of genes across 26 of these categories (excluding the category for genes which were not called as significantly differentially expressed for any of the stage comparisons—*ns_ns_ns* in table nomenclature) as well as the number of DEGs within each category that contributed to enriched GO terms for the shoot transcriptomes. Firstly, while there were variations in outcomes for the different analysis programmes individually and in J1 and J3 combinations, overall the trends were similar. For the AR comparisons, it was mostly the expression categories which contained down-regulated but not up-regulated DEGs that could be associated with enriched GO terms. For example, category *ns_ns_down* for J3 contained 1153 DEGs, 88% of which could be assigned to 49 significantly enriched GO terms; category *ns_ns_up* for J3 contained 1016 DEGs, only 4% of these could be assigned to a single enriched GO term. The *up_ns_ns* category is the exception to this; for J3, 145 DEGs were present in this category, 77% of which contributed to 41 enriched GO terms. The TC comparisons were similar in terms of the overall trend for expression categories containing exclusively down-regulated DEGs being more likely to be associated with enriched GO terms.

**Table 5:** The total number of differentially expressed genes at each expression category for each analysis method and the percentage of these DEGs associated with the total number enriched gene ontology (GO) terms for that expression category.

AR /TC Expression category		J1			J3			DESeq2			edgeR			limma-voom		
		DEGs		GO terms	DEGs		GO terms	DEGs		GO terms	DEGs		GO terms	DEGs		GO terms
		Total	%		Total	%		Total	%		Total	%		Total	%	
**AR**	**down_down_down**	166	87	40	91	67	5	139	83	31	104	80	16	122	81	26
	**down_down_ns**	34	50	4	8	-	0	32	50	3	15	-	0	12	-	0
	**down_ns_down**	187	80	11	128	88	11	184	82	14	152	88	14	129	73	6
	**down_ns_ns**	116	-	0	63	-	0	109	-	0	82	-	0	74	-	0
	**down_ns_up**	13	-	0	6	-	0	12	-	0	8	-	0	6	-	0
	**down_up_ns**	15	-	0	9	-	0	15	-	0	11	-	0	9	-	0
	**down_up_up**	5	-	0	2	-	0	5	-	0	4	-	0	2	-	0
	**down_up_down**	4	-	0	2	-	0	4	-	0	2	-	0	2	-	0
	**down_down_up**	5	-	0	0	-	-	4	-	0	0	-	0	1	-	0
	**ns_down_down**	352	81	19	220	40	3	273	86	16	273	81	26	300	71	10
	**ns_down_ns**	404	53	2	260	69	2	342	73	8	309	74	9	333	51	1
	**ns_ns_down**	1276	87	37	1153	88	49	1160	89	44	1228	88	38	1276	86	41
	**ns_ns_up**	1139	-	0	1016	4	1	996	4	1	1084	4	1	1167	-	0
	**ns_up_up**	337	-	0	250	-	0	299	-	0	298	-	0	292	6	2
	**ns_up_down**	15	-	0	6	-	0	15	-	0	13	-	0	6	-	0
	**ns_down_up**	11	-	0	3	-	0	10	-	0	7	-	0	4	-	0
	**ns_up_ns**	319	-	0	253	-	0	285	-	0	300	-	0	290	-	0
	**up_ns_up**	122	-	0	92	10	3	109	-	0	98	-	0	109	-	0
	**up_ns_ns**	201	76	53	145	77	41	170	68	42	157	73	42	181	78	35
	**up_ns_down**	11	-	0	5	-	0	10	-	0	7	-	0	6	-	0
	**up_up_ns**	29	-	0	20	-	0	26	-	0	21	-	0	23	-	0
	**up_up_up**	105	-	0	41	-	0	81	-	0	63	-	0	64	-	0
	**up_up_down**	2	-	0	1	-	0	1	-	0	1	-	0	1	-	0
	**up_down_up**	6	-	0	4	-	0	5	-	0	5	-	0	5	-	0
	**up_down_down**	6	-	0	4	-	0	4	-	0	5	-	0	5	-	0
	**up_down_ns**	23	-	0	21	-	0	24	-	0	19	-	0	23	-	0
**TC**	**down_down_down**	0	-	-	0	-	-	0	-	-	0	-	-	0	-	-
	**down_down_ns**	4	-	0	2	-	0	2	-	0	2	-	0	4	-	0
	**down_ns_down**	113	83	18	69	-	0	110	90	18	78	62	3	75	67	3
	**down_ns_ns**	212	74	11	135	79	11	186	76	10	159	77	12	165	78	11
	**down_ns_up**	33	-	0	6	-	0	31	-	0	10	-	0	7	-	0
	**down_up_ns**	69	-	0	30	-	0	68	-	0	47	-	0	34	-	0
	**down_up_up**	8	-	0	4	-	0	6	-	0	5	-	0	5	-	0
	**down_up_down**	103	-	0	62	-	0	98	-	0	76	-	0	66	-	0
	**down_down_up**	3	-	0	1	-	0	3	-	0	1	-	0	1	-	0
	**ns_down_down**	52	85	11	15	-	0	48	85	12	32	38	3	20	-	-
	**ns_down_ns**	518	63	8	381	81	18	423	87	24	406	81	13	490	64	7
	**ns_ns_down**	872	77	18	758	81	23	817	81	23	836	80	21	819	79	19
	**ns_ns_up**	866	3	1	695	3	1	713	3	1	755	3	1	875	3	1
	**ns_up_up**	63	-	0	35	-	0	53	-	0	48	-	0	39	-	0
	**ns_up_down**	178	-	0	141	-	0	153	-	0	176	-	0	164	-	0
	**ns_down_up**	228	-	0	162	-	0	202	71	12	177	71	13	190	-	0
	**ns_up_ns**	423	4	1	321	-	0	396	5	1	381	-	0	357	12	2
	**up_ns_up**	55	-	0	28	82	1	52	-	0	38	-	0	33	79	1
	**up_ns_ns**	216	-	0	153	-	0	188	-	0	166	-	0	181	-	0
	**up_ns_down**	23	-	0	16	-	0	20	-	0	21	-	0	19	-	0
	**up_up_ns**	1	-	0	0	-	-	1	-	0	1	-	0	0	-	-
	**up_up_up**	2	-	0	0	-	-	2	-	0	1	-	0	0	-	-
	**up_up_down**	1	-	0	0	-	-	1	-	0	0	-	-	0	-	-
	**up_down_up**	90	60	4	69	-	0	78	63	4	73	66	4	85	61	4
	**up_down_down**	11	-	0	4	-	0	10	-	0	7	-	0	4	-	0
	**up_down_ns**	106	-	0	63	-	0	78	60	3	69	-	0	94	-	-

Table 6 contains the equivalent information for the root transcriptomes. The data is also presented in terms of just the possible 8 combinations (excluding the *ns_ns* category) of the Early and Middle comparisons for which there is replication. For these Early and Middle comparisons, substantial numbers of DEGs were only detected by DESeq2, but a high proportions of these could be associated with GO terms for the AR-*ns_up*, AR-*ns_down* and TC-*ns_down* expression categories. The *ns_down* categories were associated with c. 3 times as many GO terms as the *ns-up* categories. When the Early, Middle and Late comparisons were included, by far the majority of DEGs were contained within the AR/TC-*ns_ns_down* and *ns_ns_up* expression categories and for both of these categories across all the analysis methods a high proportion of the associated DEGs contributed to enriched GO terms though, noticeably, the *ns_ns_down* comparisons were associated with 6–7 times more GO terms than the *ns_ns_up* comparisons. Comparing AR and TC results for the Early and Middle root transcriptome comparisons, the numbers of DEGs called in the TC comparisons were only 23% of those called in the AR comparisons (DESeq2). For the Early, Middle and Late comparisons, the equivalent figure was about 60%.

**Table 6:** The total number of root differentially expressed genes at each expression category for each analysis method and the percentage of these DEGs associated with the total number enriched gene ontology (GO) for that expression category.

**AR /TC** **Expression category**^**1**^		J1			J3			DESeq2			edgeR			limma-voom		
		DEGs		GO terms	DEGs		GO terms	DEGs		GO terms	DEGs		GO terms	DEGs		GO terms
		Total	%		Total	%		Total	%		Total	%		Total	%	
**AR**	**ns_up**	**314**	**70**	**9**	0	-	-	**314**	**70**	**9**	0	-	-	0	-	-
	**ns_down**	**528**	**88**	**26**	1	-	-	**528**	**88**	**26**	0	-	-	1	-	-
	**up_ns**	3	-	-	0	-	-	3	-	-	0	-	-	0	-	-
	**up_up**	2			0	-	-	2			0	-	-	0	-	-
	**ns_down_down**	**330**	**89**	**35**	0	-	-	**329**	**89**	**34**	0	-	-	1	-	0
	**ns_down_ns**	**195**	**70**	**16**	0	-	-	**197**	**70**	**16**	0	-	-	0	-	-
	**ns_ns_down**	**1761**	**89**	**79**	**1376**	**85**	**69**	**1624**	**90**	**84**	**1478**	**84**	**68**	**1493**	**84**	**65**
	**ns_ns_up**	**1805**	**45**	**11**	**1527**	**68**	**15**	**1556**	**66**	**14**	**1614**	**50**	**13**	**1861**	**45**	**11**
	**ns_up_up**	**156**	**53**	**8**	0	-	-	**154**	**53**	**9**	0	-	-	0	-	-
	**ns_up_down**	8	-	0	0	-	-	8	-	0	0	-	-	0	-	-
	**ns_down_up**	3	-	0	0	-	-	2	-	0	0	-	-	0	-	-
	**ns_up_ns**	**150**	**67**	**5**	0	-	-	**152**	**68**	**5**	0	-	-	0	-	-
	**up_ns_ns**	3	-	0	0	-	-	3	-	0	0	-	-	0	-	-
	**up_up_ns**	2	-	0	0	-	-	2	-	0	0	-	-	0	-	-
**TC**	**ns_up**	43	-	-	0	-	-	43	-	-	2	-	-	0	-	-
	**ns_down**	**144**	**83**	**24**	1	-	-	**144**	**83**	**24**	3	-	-	1	-	-
	**up_ns**	5	-	-	0	-	-	5	-	-	0	-	-	0	-	-
	**ns_down_down**	**21**	**29**	**1**	0	-	-	**21**	**29**	**1**	0	-	-	0	-	-
	**ns_down_ns**	**120**	**86**	**30**	0	-	-	**120**	**86**	**30**	3	-	0	0	-	-
	**ns_ns_down**	**1625**	**90**	**67**	**942**	**85**	**50**	**1514**	**90**	**65**	**1275**	**89**	**62**	**1048**	**84**	**49**
	**ns_ns_up**	**1274**	**48**	**12**	**845**	**51**	**12**	**1071**	**54**	**15**	**920**	**50**	**13**	**1141**	**44**	**9**
	**ns_up_up**	9	-	0	0	-	-	8	-	0	0	-	-	0	-	-
	**ns_up_down**	6	-	0	0	-	-	6	-	0	1	-	0	0	-	-
	**ns_down_up**	2	-	0	0	-	-	2	-	0	0	-	-	0	-	-
	**ns_up_ns**	28	-	0	0	-	-	29	-	0	0	-	-	0	-	-
	**up_ns_ns**	5	-	0	0	-	-	5	-	0	0	-	-	0	-	-

^1^Expression categories which contained 0 DEGs have been omitted for clarity.

### Enriched GO terms and related gene models in relation to expression categories

#### Leaf transcriptome

**Fig 2:**
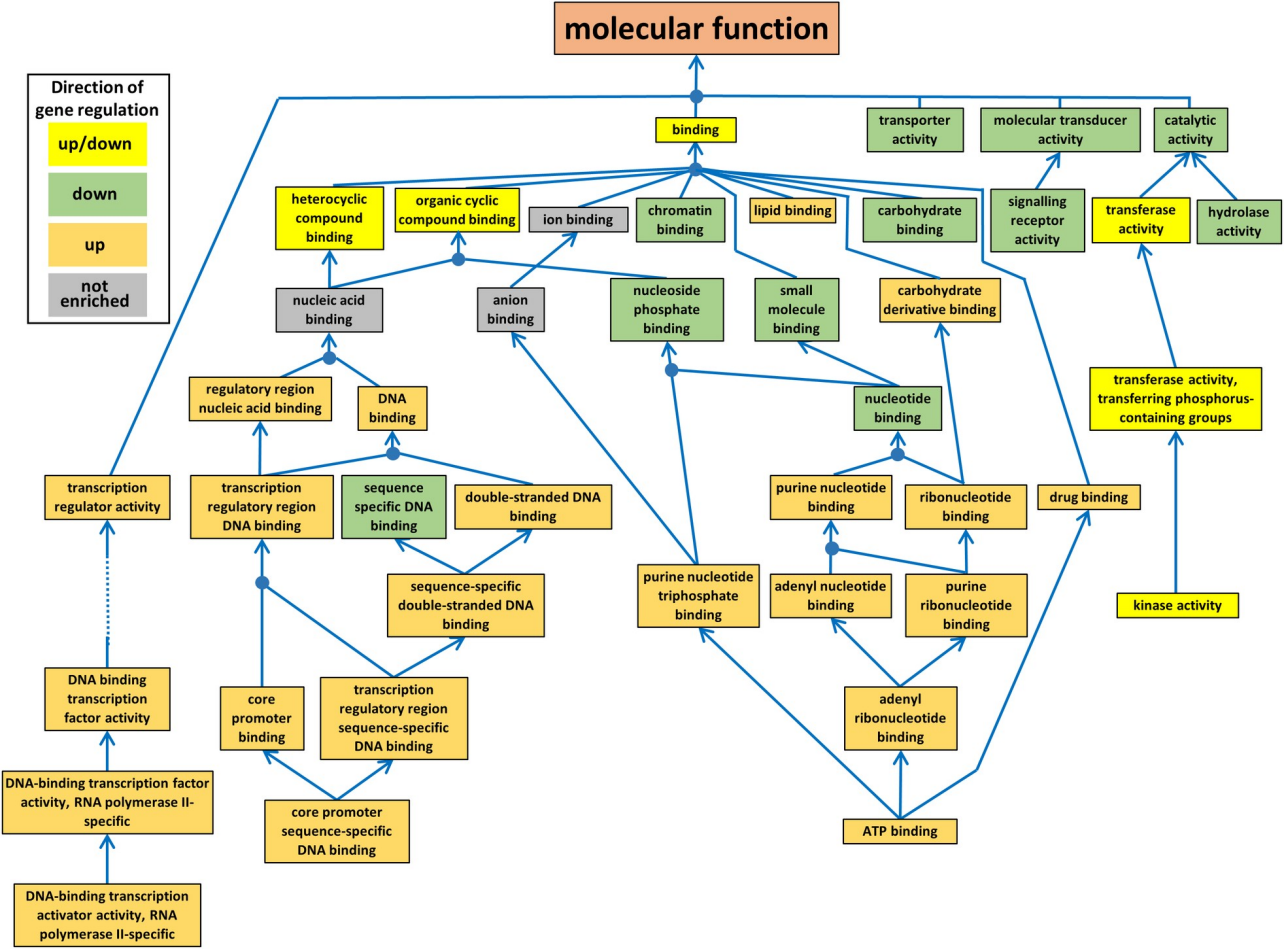
Enriched gene ontology (GO) terms associated with the Molecular Function GO category for shoot differentially expressed genes. The colour of the box indicates whether the enriched GO terms was associated with just up-regulated, just down-regulated or both up- and down-regulated DEGs across the 4 sampling points (35%, 15%, 5% and 1% EWC). Blue arrows indicate the direction of the GO hierarchy from child to parent and blue circles indicate where two or more child GO terms are associated with a single parent GO term.

Overall, when combining all of the 5 approaches (J1, J3, DESeq2, edgeR and limma-voom) 123 different GO terms were significantly enriched when the data was analysed according to the 26 expression categories described in Table 5. Of these, 91 GO terms were enriched in the 2 expression categories *AR-up_ns_ns* and *AR-ns_ns_down*, and the orders of GO terms in Tables 7 and S3 have been adjusted to reflect this. The relationship between different leaf MF GO terms is illustrated in Fig 2 and for all MF, BP and CC GO terms illustrated in S4 Fig. Results for enriched GO terms and associated gene models in Groups 1–4 (Table 7) are described below. Details of GO terms and associated gene models in Groups 5–8 (S3 Table) can be found in S1 Results.

**Table 7:** Enriched gene ontology (GO) terms associated with selected expression categories of shoot differentially expressed genes.

Group^1^	Enriched GO term				Analysis method^2^	
	i.d.	Category^3^	Position^4^	Description	AR-unn^5^	AR-nnd^5^
1	GO:0097367	MF	5	carbohydrate derivative binding	J1	-
	GO:0008144	MF	6	drug binding	J1	-
	GO:0017076	MF	6	purine nucleotide binding	J1	-
	GO:0032553	MF	6	ribonucleotide binding	J1	-
	GO:0030554	MF	7	adenyl nucleotide binding	J1	-
	GO:0032555	MF	7	purine ribonucleotide binding	J1	-
	GO:0035639	MF	7	purine ribonucleoside triphosphate binding	J1	-
	GO:0032559	MF	8	adenyl ribonucleotide binding	J1	-
	GO:0005524	MF	9	ATP binding	J1	-
2	GO:0009893	BP	5	positive regulation of metabolic process	J3,E	-
	GO:0009891	BP	6	positive regulation of biosynthetic process	J1,J3,D,E,L	-
	GO:0010604	BP	6	positive regulation of macromolecule metabolic process	J1,J3,D,E,L	-
	GO:0051173	BP	6	positive regulation of nitrogen compound metabolic process	J1,J3,D,E,L	-
	GO:0031325	BP	6	positive regulation of cellular metabolic process	J3,E	-
	GO:0010557	BP	8	positive regulation of macromolecule biosynthetic process	J1,J3,D,E,L	-
	GO:0031328	BP	8	positive regulation of cellular biosynthetic process	J1,J3,D,E,L	-
	GO:0045935	BP	8	positive regulation of nucleobase-containing compound metabolic process	J1,J3,D,E,L	-
	GO:0010628	BP	9	positive regulation of gene expression	J1,J3,D,E,L	-
	GO:0051254	BP	9	positive regulation of RNA metabolic process	J1,J3,D,E,L	-
	GO:1902680	BP	10	positive regulation of RNA biosynthetic process	J1,J3,D,E,L	-
	GO:1903508	BP	11	positive regulation of nucleic acid-templated transcription	J1,J3,D,E,L	-
	GO:0006366	BP	11	transcription by RNA polymerase II	J3	-
	GO:0045893	BP	12	positive regulation of transcription, DNA-templated	J1,J3,D,E,L	-
	GO:0006357	BP	12	regulation of transcription by RNA polymerase II	J3	-
	GO:0045944	BP	13	positive regulation of transcription by RNA polymerase II	J1,J3,D,E,L	-
	GO:0008289	MF	3	lipid binding	J1,D,L	-
	GO:0001067	MF	5	regulatory region nucleic acid binding	J1,J3,D,E,L	-
	GO:0003677	MF	5	DNA binding	J1,D,E,L	-
	GO:0003690	MF	6	double-stranded DNA binding	J1,J3,D,E,L	-
	GO:0044212	MF	6	transcription regulatory region DNA binding	J1,J3,D,E,L	-
	GO:0140110	MF	6	transcription regulator activity	J1,J3,D,E,L	-
	GO:1990837	MF	7	sequence-specific double-stranded DNA binding	J1,J3,D,E,L	-
	GO:0001047	MF	8	core promoter binding	J1,J3,D,E,L	-
	GO:0001046	MF	9	core promoter sequence-specific DNA binding	J1,J3,D,E,L	-
	GO:0001228	MF	9	DNA-binding transcription activator activity, RNA polymerase II-specific	J1,J3,D,E,L	-
	GO:0003700	MF	11	DNA-binding transcription factor activity	J1,J3,D,E,L	-
	GO:0000976	MF	14	transcription regulatory region sequence-specific DNA binding	J1,J3,D,E,L	-
	GO:0000981	MF	15	DNA-binding transcription factor activity, RNA polymerase II-specific	J1,J3,D,E,L	-
3	GO:0009987	BP	2	cellular process	J1,J3,D,E,L	J1,J3,D,E,L
	GO:0006807	BP	3	nitrogen compound metabolic process	J1,D,E	-
	GO:0071704	BP	3	organic substance metabolic process	J1,D,E	J1,J3,D,E,L
	GO:0044238	BP	4	primary metabolic process	J1,J3,D,E	J1,J3,D,E,L
	GO:0044237	BP	4	cellular metabolic process	J1,J3,D,E	J3,L
	GO:0044260	BP	5	cellular macromolecule metabolic process	J1,D,E	-
	GO:0043412	BP	5	macromolecule modification	J1,J3,D,E	J3,L
	GO:0044267	BP	6	cellular protein metabolic process	J1,D,E	J3,L
	GO:0036211	BP	6	protein modification process	J1,J3,D,E	J3,L
	GO:0006464	BP	7	cellular protein modification process	J1,J3,D,E	J3,L
	GO:0016020	CC	3	membrane	J1,J3,L	J1,J3,D,E,L
	GO:0071944	CC	4	cell periphery	J1,J3,D,E,L	J1,J3,D,E,L
	GO:0005886	CC	5	plasma membrane	J1,J3,D,E,L	J1,J3,D,E,L
	GO:0005488	MF	2	binding	J1,J3,D,E,L	J1,J3,D,E,L
	GO:0016740	MF	3	transferase activity	J1,E,L	J1,J3,D,E,L
	GO:0097159	MF	3	organic cyclic compound binding	J1,J3,D,E,L	J1,J3,D,E,L
	GO:1901363	MF	3	heterocyclic compound binding	J1,J3,D,E,L	J1,J3,D,E,L
	GO:0016772	MF	4	transferase activity, transferring phosphorus-containing groups	J1,J3,D,L	J1,J3,D,E,L
	GO:0016301	MF	5	kinase activity	J1,J3,D,L	J1,J3,D,E,L
4	GO:0008152	BP	2	metabolic process	-	J1,J3,D,E,L
	GO:0050896	BP	2	response to stimulus	-	J1,J3,D,E,L
	GO:0009056	BP	3	catabolic process	-	J1,J3,D,E,L
	GO:0009058	BP	3	biosynthetic process	-	J1,J3,D,E,L
	GO:0009719	BP	3	response to endogenous stimulus	-	J1,J3,D,E,L
	GO:0019748	BP	3	secondary metabolic process	-	J1,J3,D,E,L
	GO:0005975	BP	4	carbohydrate metabolic process	-	J1,J3,D,E,L
	GO:0006091	BP	4	generation of precursor metabolites and energy	-	J1,J3,D,E,L
	GO:0006629	BP	4	lipid metabolic process	-	J1,J3,D,E,L
	GO:0015979	BP	4	photosynthesis	-	J1,J3,D,E,L
	GO:1901564	BP	4	organonitrogen compound metabolic process	-	J3
	GO:0019538	BP	5	protein metabolic process	-	J3
	GO:0005576	CC	2	extracellular region	-	J1,J3,D,E,L
	GO:0005623	CC	2	cell	-	J1,J3,D,E,L
	GO:0043226	CC	2	organelle	-	J3,D
	GO:0044464	CC	3	cell part	-	J1,J3,D,E,L
	GO:0030312	CC	5	external encapsulating structure	-	J1,J3,D,E,L
	GO:0043227	CC	5	membrane-bounded organelle	-	J3,D
	GO:0005737	CC	6	cytoplasm	-	D
	GO:0005618	CC	6	cell wall	-	J1,J3,D,E,L
	GO:0009579	CC	6	thylakoid	-	J1,J3,D,E,L
	GO:0043229	CC	6	intracellular organelle	-	J3,D
	GO:0012505	CC	6	endomembrane system	-	J3,D,E
	GO:0043231	CC	7	intracellular membrane-bounded organelle	-	J3,D
	GO:0044444	CC	7	cytoplasmic part	-	J3,D
	GO:0005794	CC	8	Golgi apparatus	-	J1,D,E
	GO:0009536	CC	8	plastid	-	J1,J3,D,E,L
	GO:0003824	MF	2	catalytic activity	-	J1,J3,D,E,L
	GO:0005215	MF	2	transporter activity	-	J1,J3,D,E,L
	GO:0016787	MF	3	hydrolase activity	-	J1,J3,D,E,L
	GO:0030246	MF	3	carbohydrate binding	-	J1,J3,D,E,L
	GO:0036094	MF	4	small molecule binding	-	J1,J3,D,E,L
	GO:1901265	MF	4	nucleoside phosphate binding	-	J1,J3,D,E,L
	GO:0000166	MF	5	nucleotide binding	-	J1,J3,D,E,L

^1^Only Groups 1–4 are shown. Full details on all Groups 5–8 are given in S3 Table

^2^Analysis methods identifying significant DEGs associated with the indicated enriched GO term and expression category. D = DESeq2, E = edgeR and L = limma-voom.

^3^MF = Molecular Function; BP = Biological Process; CC = Cellular component.

^4^Ascending numbers indicate a more specific category in the GO terms hierarchies.

^5^Abbreviated expression categories. u = up; d = down; n = ns.

Considering GO terms enriched in AR-*up_ns_ns* but not in AR-*ns_ns_down* (Table 7, Groups 1 and 2), 2 related groups of GO terms were present: a) 7 MF GO terms associated with nucleoside/nucleotide binding, which were detected just by J1 and, b) 29 BP and MF GO terms associated with positive regulation of cellular processes and DNA binding/transcription factor activity, the majority of which were detected by all the analysis methods. The most-specific child GO term (referred to as the child GO term from this point onwards) for Group 1 was *ATP binding* (GO:0005524) which is clearly fundamental to many plant metabolic processes. Two of the 5 associated gene models (i.e., the 5 gene models which constituted the significant enrichment of the GO categories in Group 1 using BLAST2GO) have been annotated as ‘disease resistance’ proteins (S4A Table). The enrichment for these GO terms was only supported using one of the analysis approaches (J1), suggesting caution in interpretation of the significance. Group 2 contained 37 different gene models. The child GO terms for Group 2 were *positive regulation of transcription by RNA polymerase II* (BP, GO:0045944), *core promoter sequence-specific DNA binding* (MF, GO:0001046) and *DNA-binding transcription activator activity*, *RNA polymerase II-specific* (MF, GO:0001228). Not surprisingly, many of the associated gene models have been annotated as being potential transcription factors (S4A Table), though, this group also contained gene models associated with histones H1 and CENH3 as well as DNA and RNA polymerases. The majority of the Group 1 and 2 GO terms were largely absent from the other expression categories, indicating that the up-regulation of the associated gene models in the Early stage may have been quite specific to the onset of water-stress (at least for Group 2). However, three of the same GO terms were also enriched in expression category *TC-up_down_up*. Of the associated 17 gene models which constituted the enrichment in this case, 4 were in common with *AR-up_ns_ns* Group 2, including the Histone1 and CENH3, but the remaining 13 represent a different set of (largely) transcription factors, 6 of which were annotated as containing a WRKY domain and 2 heat stress/shock factors. WRKY domain and heat stress/shock containing transcription factors play multiple roles in plant stress responses and the high proportion of these in this group, in combination with the heat stress/shock factors, indicates a potential relevance to drought response.

Group 3 was enriched for BP, CC and MF GO terms in expression category AR-*up_ns_ns* and in many expression categories which showed down-, but not subsequent up-regulation in Middle or Late comparison stages (Tables 7 and S3 and Figs 2 and S4). The child term for the BP, CC and MF GOs were *cellular protein modification process* (GO:0006464), *plasma membrane* (GO:0005886) and *kinase activity* (GO:0016301), respectively, indicating an up-regulation of gene models involved in processes at the plasma membrane during the onset of water-stress–followed by a down-regulation of similar processes as the water-stress becomes more severe–(though not down-regulation of the same gene models significantly below the AR or TC reference significance levels). Group 3 AR-*up_ns_ns* contained 168 gene models within the enriched GO terms, compared to 1117 gene models for expression categories which showed down-, but not subsequent up-regulation in Middle or Late comparison stages. The annotations of the gene models indicated a representation of potential stress-response, transporter, transcription factor and kinase activities associated with both up- and down-regulated expression categories. While, both up- and down-regulated expression categories contained some annotations relating to plant hormones, the down-regulated categories included a number of auxin and ethylene associated transcription factors as well as gibberellin and cytokinin metabolic enzymes. The down-regulated gene expression category also contained 51 gene models annotated as ‘chloroplastic’ compared to a single gene model in the up-regulated expression category.

Group 4 consisted of GO terms which were not enriched in the AR-*up_ns_ns* expression category but were enriched across most of the analysis methods in the AR-*ns_ns_down* expression category as well as, to a lesser extent, other down regulated expression categories in the Middle or Late comparison stages. The child terms for these GO categories were, BP—*protein metabolic process* (GO:0019538), CC—*Golgi apparatus* (GO:0005794) and *plastid* (GO:0009536), and MF—*nucleotide binding* (GO:0000166) (Tables 7 and S3 and Figs 2 and S4). Together, these GO terms suggest that, particularly at the Late comparison stage, there is a down regulation of genes associated with metabolism more generically, i.e. affecting secondary metabolic, carbohydrate, lipid, photosynthetic and protein metabolic processes (BP terms) along with a reduction in nucleotide binding activity (i.e., ATP-binding). The most specific CC terms enriched were *Golgi apparatus* and *plastid*, which is compatible with reductions in post-translational modification of proteins, protein trafficking and photosynthetic activity likely to accompany a reduction in overall cell metabolic activity. Group 4 enriched GOs contained 2124 gene models, 193 of which contained the annotation ‘kinase’. Additional relatively highly represented terms included ‘chloroplastic’, ‘synthase’, ‘transporter’, ‘binding’, and ‘resistance’, all of which occurred in more than 50 of the gene models (S4A Table).

#### Root transcriptomes

**Fig 3:**
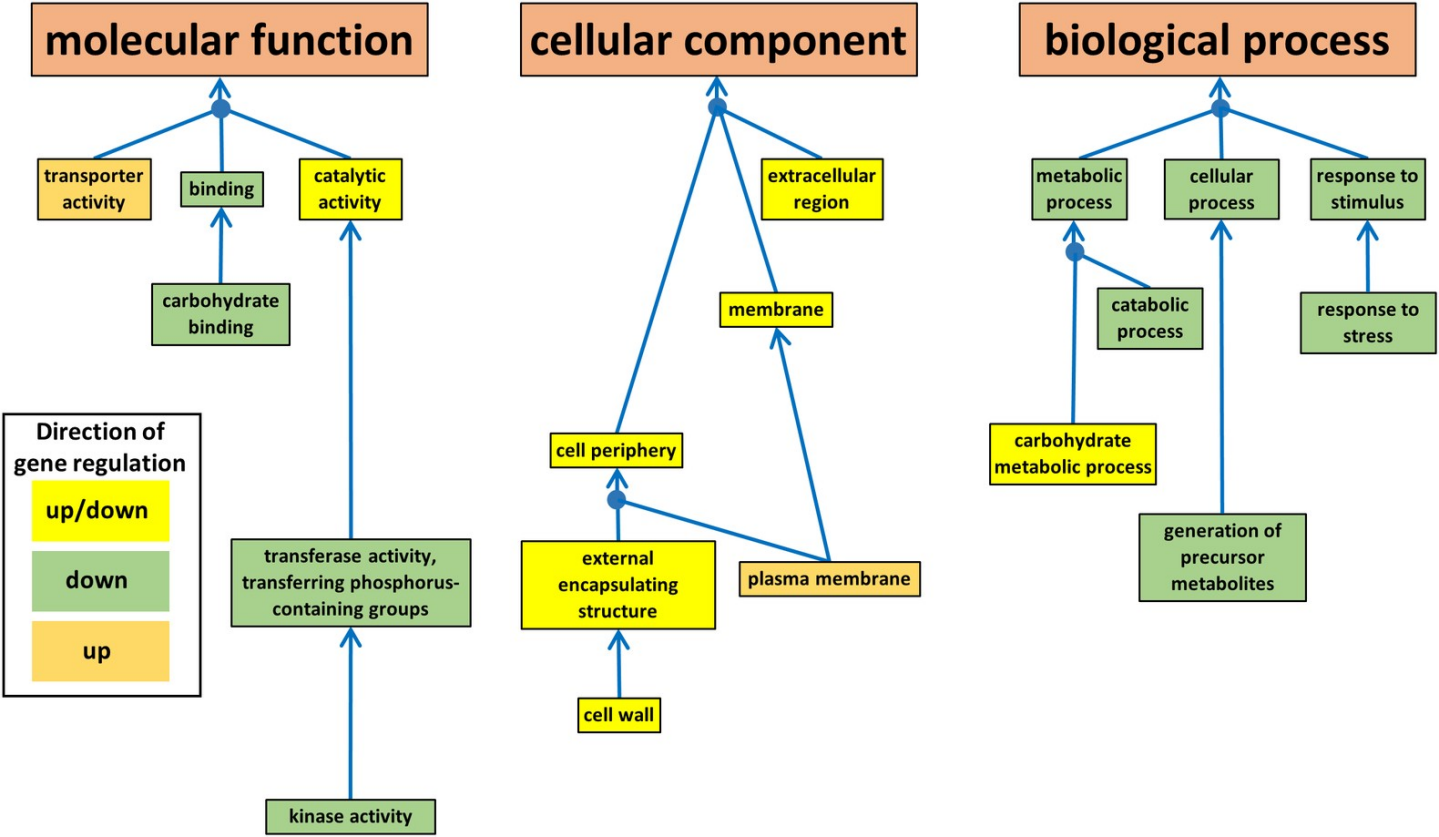
Enriched gene ontology (GO) terms associated with Molecular Function, biological process and cellular compartment GO categories for the root differentially expressed genes. The colour of the box indicates whether the enriched GO terms was associated with just up-regulated DEGs, just down-regulated DEGs or both up- and down-regulated DEGs across the 3 sampling points (35%, 15% and 5% EWC). Blue arrows indicate the direction of the GO hierarchy from child to parent and blue circles indicate where two or more child GO terms are associated with a single parent GO term.

Analysis of the root transcriptome identified 31 different enriched GO terms across the 8 Early and Middle expression categories and 110 enriched GO terms across the 26 Early, Middle and Late expression categories (described in Tables 8 and 9 and Figs 3 and S5 Expression Categories Roots A and B). For the Early and Middle comparisons (Table 8 and Fig 3), 3 Groups of GO terms were identified, relating to up-regulation (Group 1), up and down-regulation, (Group 2) and down-regulation at the Middle stage (Group 3). No GO terms were associated with early stage up- or down-regulation. As described earlier, all GO terms were associated with DEGs called only by DESeq2. Only 2 GO terms were contained within Group1, *plasma membrane* (CC, GO:0005886) and *transporter activity* (MF, GO:0005215). Of the associated 58 gene models, 39 had annotations containing ‘transporter’, ‘transport’, ‘antiporter, ‘permease’ or ‘channel’ and a further 8 were described as membrane transport proteins in Uniprot, including 3 aquaporins. Annotations also included 4 auxin transporters and one auxin induced transmembrane protein (S4B Table).

**Table 8:** Enriched gene ontology (GO) terms associated with selected expression categories of root differentially expressed genes.

Group^1^	Enriched GO term				Analysis method^2^		
	i.d.	Category^3^	Position^4^	Description	AR-nu^5^	AR-nd^5^	TC-nd^5^
1	GO:0005886	CC	5	plasma membrane	J1,D	-	-
1	GO:0005215	MF	2	transporter activity	J1,D	-	-
2	GO:0005975	BP	4	carbohydrate metabolic process	J1,D	J1,D	J1,D
2	GO:0005576	CC	2	extracellular region	J1,D	J1,D	J1,D
2	GO:0016020	CC	3	membrane	J1,D	J1,D	-
2	GO:0071944	CC	4	cell periphery	J1,D	J1,D	-
2	GO:0030312	CC	5	external encapsulating structure	J1,D	J1,D	J1,D
2	GO:0005618	CC	6	cell wall	J1,D	J1,D	J1,D
2	GO:0003824	MF	2	catalytic activity	J1,D	J1,D	J1,D
3ab	GO:0008152	BP	2	metabolic process	-	J1,D	J1,D
3ab	GO:0009987	BP	2	cellular process	-	J1,D	J1,D
3ab	GO:0050896	BP	2	response to stimulus	-	J1,D	J1,D
3a	GO:0019748	BP	3	secondary metabolic process	-	J1,D	-
3a	GO:0009628	BP	3	response to abiotic stimulus	-	J1,D	J1,D
3ab	GO:0009056	BP	3	catabolic process	-	J1,D	J1,D
3a	GO:0044237	BP	3	cellular metabolic process	-	J1,D	J1,D
3ab	GO:0006950	BP	3	response to stress	-	J1,D	-
3ab	GO:0006091	BP	4	generation of precursor metabolites and energy	-	J1,D	J1,D
3a	GO:0015979	BP	4	photosynthesis	-	J1,D	J1,D
3a	GO:0005623	CC	2	cell	-	J1,D	J1,D
3a	GO:0044464	CC	3	cell part	-	J1,D	J1,D
3a	GO:0005622	CC	4	intracellular	-	J1,D	J1,D
3a	GO:0044424	CC	5	intracellular part	-	-	J1,D
3a	GO:0043227	CC	5	membrane-bounded organelle	-	-	J1,D
3a	GO:0009579	CC	6	thylakoid	-	J1,D	J1,D
3a	GO:0005737	CC	6	cytoplasm	-	J1,D	J1,D
3a	GO:0044444	CC	7	cytoplasmic part	-	J1,D	J1,D
3a	GO:0043231	CC	7	intracellular membrane-bounded organelle	-	-	J1,D
3a	GO:0009536	CC	8	plastid	-	J1,D	J1,D
3ab	GO:0005488	MF	2	binding	-	J1,D	J1,D
3ab	GO:0030246	MF	3	carbohydrate binding	-	J1,D	-
3b	GO:0016772	MF	5	transferase activity, transferring phosphorus-containing groups	-	J1,D	-
3b	GO:0016301	MF	7	kinase activity	-	J1,D	-

^1^Group3a = GO terms enriched only if DEGs with chloroplast-related annotations are included; Group3b = GO terms enriched only if DEGs containing chloroplast-related annotations are omitted; Group 3ab = GO terms which are enriched irrespective of the inclusion or omission of DEGs with chloroplast-related annotations.

^2^Analysis methods identifying significant DEGs associated with the indicated enriched GO term and expression category. D = DESeq2, E = edgeR and L = limma-voom.

^3^MF = Molecular Function; BP = Biological Process; CC = Cellular component.

^4^Ascending numbers indicate a more specific category in the GO terms hierarchies.

^5^Abbreviated expression categories. u = up; d = down; n = ns.

**Table 9:** KEGG enzyme activity codes for which significantly different numbers of associated differentially expressed genes were present in comparisons of ‘mirror image’ expression categories.

Tissue	Expression category comparison^1^	Number of DEGs in each expression category		Number of DEGs associated with KEGG enzyme activity codes in the contrasted expression categories			
				KEGG enzyme activity code	DEGs		P^2^
	AR/TC-*1_2*	1	2		1	2	
**Shoot**	AR-*uuu_ddd*	105	166	EC:3.1.3.16—phosphatase	5	1	3.39E-02
	AR-*nun_ndn*	319	404	EC:3.6.1.15—phosphatase	3	26	<1.00E-04
				EC:3.6.1.3—adenylpyrophosphatase	1	15	1.50E-03
	AR-*nnu_nnd*	1139	1276	EC:1.11.1.7—lactoperoxidase	4	21	1.89E-03
				EC:3.6.1.15—phosphatase	17	50	2.70E-04
				EC:3.6.1.3—adenylpyrophosphatase	12	35	2.87E-03
	TC-*nud_ndu*	178	228	EC:3.6.1.15—phosphatase	2	12	2.73E-02
				EC:3.6.1.3—adenylpyrophosphatase	0	10	3.08E-03
	TC-*nun_ndn*	423	518	EC:3.6.1.15—phosphatase	4	18	1.51E-02
	TC-*nnu_nnd*	866	872	EC:1.11.1.7—lactoperoxidase	3	14	9.34E-04
**Root**	AR-*nu_nd*	314	528	EC:1.11.1.7—lactoperoxidase	3	21	9.68E-03
				EC:4.1.1.2—decarboxylase	0	20	6.73E-04
	AR-*nuu_ndd*	156	330	EC:1.11.1.7—lactoperoxidase	1	12	7.05E-02
				EC:4.1.1.2—decarboxylase	0	18	1.25E-03
	AR-*nnu_nnd*	1805	1761	EC:1.11.1.7—lactoperoxidase	9	44	<1.00E-04
				EC:3.6.1.15—phosphatase	39	86	<1.00E-04
				EC:3.6.1.3—adenylpyrophosphatase	33	63	1.28E-03
	TC-*nnu_nnd*	1274	1625	EC:3.1.3.16—phosphatase	18	9	1.93E-02
				EC:1.11.1.7—lactoperoxidase	6	45	<1.00E-04
				EC:3.6.1.15—phosphatase	28	87	<1.00E-04
				EC:3.6.1.3—adenylpyrophosphatase	21	65	1.60E-04

^1^Abbreviated expression categories. *u* = *up*; *d* = *down*; *n* = *ns*.

^2^P-values were calculated using Fisher’s Exact Test comparing the total number of DEGs in each of expression categories 1 and 2 with the corresponding values associated with the KEGG enzyme activity codes.

Group 2 contained 7 enriched GO terms, with child GO terms of *carbohydrate metabolic process* (BP, GO:0005975), *cell wall* (CC, GO:0005618) and *catalytic activity* (MF, GO:0003824) (Table 8 and Fig 3). Group 2 AR-*ns_up* enriched GO terms contained 219 gene models. Focussing on the *carbohydrate metabolic process* and *cell wall* GO categories, the most obvious difference between the up- and down-regulated gene models was the number of annotations associated with ‘germin’ or ‘peroxidase’. Of the 43 up-regulated gene models in these categories, none were annotated as ‘germin’ and only 2 as ‘peroxidase’; in the down-regulated gene models, 46 (40%) were annotated as ‘germin’ and 22 (19%) as ‘peroxidase’. This indicates substantially different metabolic activities for the up- and down- regulated gene-models within Group 2 acting on the cell wall (S4B Table).

Group 3a/ab (Table 8 and Fig 3) consisted of 439 down-regulated gene models with child GO terms of *photosynthesis* (BP, GO:0015979), *plastid* (CC, GO:0009536) and *carbohydrate binding* (MF, GO:0030246). Of the 439 gene models, 83 (19%) contained the annotation ‘*chloroplastic*’ or ‘*chlorophyll*’. The average count data for these 83 gene models at 35% EWC from the root data was c. 90x less than that for the same gene models at the same stage from the shoot data. Thus, the enrichment for these GO terms may not be particularly significant in a biological context. When the chloroplast-associated gene models were omitted from the AR- and TC-*ns_down* expression categories, the enriched GO terms that remained (Group 3b/ab) were *metabolic process* (BP, GO:0008152), *cellular process* (BP, GO:0009987), *response to stimulus* (BP, GO:0050896), *catabolic process* (BP, GO:0009056), *response to stress* (BP, GO:0006950), *generation of precursor metabolites and energy* (BP, GO:0006091), *binding* (MF, GO:0005488) and *carbohydrate binding* (MF, GO:0030246)–and a further 2 were identified: *transferase activity*, *transferring phosphorus-containing groups* (MF, GO:0016772) and *kinase activity* (MF, GO:0016301). While most of these Group 3b/ab GO terms are quite general, these latter two groups were more defined and contained 53 gene models, the majority of which were annotated as receptor-kinases (S4 Table). This indicates a likely transmembrane, cell-surface location and down-regulation of cell-signalling functions.

Details of GO terms relating to different expression categories and analysis methods when the unreplicated 1% EWC stage for the root data is included can be found in S2 Results.

### KEGG pathways

Significantly up- and down-regulated gene models from ‘mirror-image’ expression categories (i.e., AR-*up_ns_ns* and AR-*down_ns_ns*; TC-*ns_ns_up* and TC-*ns_ns_down*; AR-*up_down_up* and AR-*down_up_down*, etc.) were compared in terms of the number of up- and down-regulated gene models which could be associated with particular enzyme codes from the KEGG pathways and significant differences identified (Fisher’s Exact Test; p<0.05). As illustrated in Table 9, five enzyme codes were identified across root and shoot samples, with EC3.6.1.15 (phosphatase), EC3.6.1.3 (adenylpyrophosphatase) and EC1.11.1.7 (lactoperoxidase) the most frequently represented. For all but 2 of the 17 enzyme codes occurrences (excluding and AR-*nu_nd* and TC-*nud_ndu* in which up- and down-regulation are present on both sides of the comparison) represented by significantly different numbers of up- and down-regulated DEGs, there were a greater number of down-regulated than up-regulated DEGs.

## Discussion

Water-stress, like many environmental abiotic stresses, is unlikely to have a sudden onset. The stress will increase gradually over time as available water diminishes in the soil. Consequently, plants exposed to water-stress will adapt gradually over time. Thus, when looking at the effects of increasing water-stress in terms of changes in the transcriptome, it is important to remember that we are sampling the plant transcriptome at discrete points along what is likely to be a continuum of changing patterns of gene expression. In addition, while there may be genes that have major effects which can act independently of genetic background or, at least, the effects of which can be observed across multiple genetic backgrounds, in seeking to improve the resilience of plants in the face of water limitation, we are trying to manipulate interacting biological processes and gene family effects [63–66]. For highly heterogeneous and outbreeding perennial populations, such as perennial ryegrass, which are widely distributed across multiple environments this is likely to be particularly true [2]. Thus, in the present study, the emphasis has not been on trying to identify candidate genes as such, but more to describe and compare, through the lens of GO terms, more generalised biological processes occurring in the leaves and roots of perennial ryegrass with increasing water stress. The genotype under study is, in some ways, atypical in that it is an inbred and, as a consequence, largely homozygous genotype. This genotype was chosen as it was used for the published genome and physical map assemblies for perennial ryegrass [41, 67] and so brings direct advantages to the analysis in terms of the quality of the available gene annotations. Additionally, as this study directly references the published perennial ryegrass genome assembly, it is hoped that this will also contribute to the development of a genomic platform for the further genetic dissection of the drought response in a wider selection of perennial ryegrass germplasm reflecting both agricultural and landscape adaptations.

The identification of GO terms associated with the progression of water-stress depends on the range of DEGs identified. This, itself, is dependent on the type of analysis undertaken. In this study, one aspect that we were interested in was whether differences in DEGs identified by different analysis software tools affected the range of associated GO terms. Additionally, we wished to see how using a ‘jury’ system (J1 and J3) affected the range of GO terms. The choice of the 3 programmes was partly that they incorporate different assumptions about data distributions, normalisation and significance testing but also because they are widely used and have performed well in larger-scale comparisons, including studies where replicate number has been limited [30–32]. Additionally, Costa-Silva et al. [29] suggested that combining the results of analysis programmes can provide more reliable results (with reliability being equated with qRT-PCR replication in that study).

### Comparisons of DESeq2, edgeR, limma-voom, J1 and J3

#### Shoot transcriptomes

Table 10 describes the numbers and % of GO terms detected by each combination of methods. Focussing on the AR comparisons (a discussion of AR versus TC comparisons can be found in S1 Discussion), the largest proportion of GO terms were detected by all the methods. When the methods are considered individually, J1, DESeq2 and edgeR (in that order) detected the most enriched GO terms and limma-voom and J3 the least. It is, perhaps, slightly surprising that most enriched GO terms are generated by the DEGs in the J1 set and the least by the J3 set. J1 contains more DEGs than J3 by design, but the J1 set could be considered to be less stringently selected than the J3 set (called by any of the tools as opposed to all of the tools) so, possibly, more likely to accumulate false positives and less likely to be within the FDR criteria for the Fisher’s Exact Test. But this appears not to have been the case.

**Table 10:** The number and proportion of enriched gene ontology (GO) terms detected by the different analysis methods for the shoot data.

Analysis method combination	Enriched GO terms for shoot					
	AR+TC		AR		TC	
	n	%	n	%	n	%
**J1,J3,D,E,L**	110	32	80	36	30	23
**J1,D**	40	11	18	8	22	17
**J1,D,E**	20	6	15	7	5	4
**D,E**	20	6	8	4	12	9
**D**	19	5	2	1	17	13
**J1**	16	5	14	6	2	2
**J1,D,E,L**	16	5	9	4	7	5
**E**	16	5	12	5	4	3
**J3**	11	3	7	3	4	3
**J3,D,E**	11	3	6	3	5	4
**J1,D,L**	9	3	8	4	1	1
**L**	9	3	6	3	3	2
**J3,E**	7	2	3	1	4	3
**J3,L**	7	2	5	2	2	2
**J3,D**	7	2	5	2	2	2
**J1,L**	7	2	6	3	1	1
**J1,J3,D,E**	6	2	6	3	0	0
**J1,E,L**	4	1	4	2	0	0
**J1,J3,D,L**	3	1	2	1	1	1
**J3,D,E,L**	3	1	0	0	3	2
**J1,E**	3	1	3	1	0	0
**J1,J3,D**	3	1	0	0	3	2
**J1,J3,L**	1	<1	1	<1	0	0

Looking more closely at the distribution of enriched AR expression category GO terms according to method (Table 7) Group 1 was exceptional in that only J1 DEGs were significantly enriched for the GOs in this section, the child term for which was *ATP binding* (GO:0005524). These connected GO terms (Fig 2) were only associated with 5 gene models and the probability values were close to the FDR threshold. Additionally, only 2 of these gene models were called as DEGs by all 3 programmes. So, this may suggest less confidence in these Group 1 GO terms as reflecting underlying biological processes specifically associated with the Early comparison stage. For the expression stages for which most DEGs were identified across all methods (AR *up_ns_ns* and *ns_ns_down*) for Group 2, 23 out of the 29 GO terms were enriched across all the methods; for Group 4, 24 out of the 34 GO terms were enriched across all methods. For Group 3, which contains GO terms that were enriched in both expression stages, the proportions were slightly less with 6 and 12 out of 19 of the GO terms being enriched across all methods for AR *up_ns_ns* and *ns_ns_down*, respectively. It is also worth noting that using just a single programme would have suggested either up-regulation or down-regulation of some GO terms (particularly within the BP category) within Group 3 AR *up_ns_ns* and *ns_ns_down*, but not both up- and down-regulation. More generally, it is also clear that the overall pattern of biological processes, at least when described through enriched GO terms, is certainly nuanced by the choice of analysis method.

#### Root transcriptomes

For the root transcriptomes, for the Early and Middle stage comparisons (Table 8), the choice of programme makes a major difference to the number of enriched GO terms detected. DESeq2 identified 844 DEGs and 35 enriched GO terms at the Middle comparison stage, compared to only 1 DEG detected by either of edgeR or limma-voom (Table 3). If we compare these GO terms with those identified when the Late comparison is included, it is interesting that all of the non-chloroplast-associated GO terms just detected by DESeq2 at the Middle stage are also detected by all of the programmes, up- or down-regulated, in the AR/TC-*ns_ns_down* expression categories–which tends to supports their detection. Conversely, the chloroplast-associated GO terms (i.e., Table 8, Group 3) were not detected in the AR/TC-*ns_ns_down* expression categories by any of the programmes and only in the AR/TC-*ns_down_ns* and AR-*ns_down_down* expression categories by DESeq2 (S5 Table). Thus, DSEeq2 may have been able to identify more marginal changes in differential expression patterns at an earlier stage, but this may have also been at the risk of generating GO terms that are less well supported.

### Patterns of enriched GO terms

During this discussion we will be considering GO terms which were enriched by any of the analysis methods, as described in Tables 7–9 and S3 and S5.

Probably the most striking aspect of this study of changes in gene expression in response to drought through enriched GO terms was the distinction between up- and down-regulated categories of gene models for the shoot transcriptome. With the exception of expression stage AR-*up_ns_ns*, very few GO terms were enriched at any of the other expression stages which indicated any up-regulation (18 expression categories, 33 enriched GO terms in total). This is a particularly stark comparison when we compare AR-*ns_ns_down* with AR *ns_ns_up*. Averaging across all analysis methods (Table 5) within AR-*ns_ns_down* there was an average of 1219 down-regulated DEGs, 87% of which could be associated with 42 enriched GO terms. The equivalent figures for AR *ns_ns_up* were 1080 up-regulated DEGs, 11% of which could be associated with 1 enriched GO term. A similar, but reverse, pattern can be seen comparing AR-*up_ns_ns* with AR *down_ns_ns* where the equivalent figures are 171 up-regulated DEGs, 74% of which are associated with 43 enriched GO terms and 89 down-regulated DEGs associated with 0 enriched GO terms. So, while there is an approximate balance in the overall numbers of up- and down-regulated DEGs across all expression categories (Table 4), there is a distinct imbalance in the degree to which they contribute to enriched GO terms and so indicate co-ordinated biological processes. Hong et al. [68], using mammalian cancer tumour datasets, showed that gene pairs with functional links in KEGG pathways tended to have positively correlated expression levels and proposed that analysing up- and down-regulated genes separately was more powerful than analysing all of the DEGs genes together—and this would also seem to be true in the present case. Due to different experimental designs and lack of reporting of separate analysis for up- and down-regulated genes in some cases, it is difficult to make direct comparisons with previously published studies in plant systems. However, Wang et al [69] and Kokáš et al [11] comparing drought-stressed and control *Festuca mairei* and barley, respectively, reported that up-and down- regulated genes fell into distinct GO categories–though this is not quite the same as the results we present here. The imbalance detected for the shoot transcriptomes, in terms of the relationship between direction of DEG regulation and the number of enriched GO terms, was not so apparent for the root transcriptomes, though we are dealing with a much smaller set of DEGs. AR-*ns_up* and *ns_down* were more or less equivalent (particularly if the GO terms associated with the chloroplast-related DEGs are omitted). However, a comparison of AR/TC-*ns_ns_up* with *ns_ns_down* did indicate a higher percentage of DEGs contributing to more enriched GO terms in the latter expression category compared to the former. It will be interesting to see if this can be confirmed in future work.

It is an observation of this study that while the overall balance in terms of the numbers of up- and down-regulated DEGs at the different stages is apparent, the association of these two groups of DEGs with enriched GO terms is markedly unbalanced and this raises the question as to why so many DEGs in some expression categories were associated with so few or no enriched GO terms. Many studies of differential gene expression show this pattern of overall balance in terms of numbers of up- and down- regulated DEGs at time points and it is interesting to speculate as to whether this balance serves a function in terms of maintaining the molecular equilibrium of the cell, with the impact of the balancing overall level of gene expression ‘uncoupled’ from a more coordinated effect on cell phenotype, thus generating fewer enriched GO terms.

### KEGG pathway activities

The comparison of ‘mirror-image’ expression categories (Table 9) identified 5 KEGG enzyme activity codes represented across the various expression categories associated with significantly different numbers of up- and down-regulated DEGs. As described earlier, the number of down-regulated DEGs associated with enzyme codes exceeded the number of up-regulated DEGs associated with expression codes the majority of the time. The most commonly occurring enzyme codes were EC3.6.1.15 (phosphatase) and EC3.6.1.3 (adenylpyrophosphatase) occurring across 6 and 5 expression categories, respectively. Both enzyme activities are nucleoside triphosphatases, EC3.6.1.3 being specifically an ATP phosphohydrolase and EC3.6.1.15 also having phosphohydrolase activity for nucleoside tri/diphosphates, thiamine diphosphate and flavin adenine dinucleotide. Across all expression categories described in Table 9, EC3.6.1.15 was associated with 268 down-regulated DEGs and 97 up-regulated DEGs with a further 14 DEGs showing evidence of both up- and down-regulation. The equivalent figures for EC3.6.1.3 were 178, 67 and 10 for down-, up and both down- and up regulated DEGs, respectively. Thus, the DEGS associated with both these key enzyme activities involved in driving fundamental cellular metabolic and cytoskeletal processes, showed overall down-regulation, which may well indicate the general slowing of cellular metabolic processes as the drought proceeds.

The other frequently represented enzyme activity code, occurring across 5 different expression categories (excluding AR-*nu_nd*) was EC1.11.1.7 (lactoperoxidase). This is an enzyme activity involved in the phenylpropanoid biosynthesis pathway and associated with the final stages of lignin biosynthesis in the development of secondary plant cell walls [70]. DEGs associated with this enzyme activity were also more often down- (157) rather than up-regulated (26) across shoot and root (Table 9). A number of previous studies have looked at the activities and/or expression profiles of enzymes from the phenylpropanoid pathway under drought stress and opposite or mixed trends, in terms of their regulation, have been reported [71–74]–though there are considerable variations in the species under study, experimental methodologies and time courses across studies. However, the frequent occurrences of genes involved in the phenylpropanoid pathway in drought studies does indicate the significance of cell-wall metabolism in the response to water-stress.

The other two enzyme classes were EC3.1.3.16 (phosphatase– 2 occurrences) and EC4.1.1.2 (decarboxylase– 2 occurrence). EC3.1.3.16, includes enzymes which show protein-serine/threonine phosphatase activity against a wide range of proteins and was the only enzyme class which showed predominantly up-regulated DEGs in the shoot (AR-*uuu/ddd*) and the root (TC-*nnu/nnd*). The second enzyme class, EC4.1.1.2, was identified just in the root during the AR-*nu_nd* AR_*ns_down_down/ns_up_up* comparison stage. All 20 of the DEGs associated with this enzyme class were down-regulated and annotated as ‘germin’. EC4.1.1.2 is specifically an oxalate de-carboxylase and accumulation of malic (leaves) and fumaric (roots) acids in perennial ryegrass in response to PEG-induced water-stress has been reported [17]. Similar observations for malic, fumaric and oxalic acids have been reported as a drought response in wheat leaves[75]. The observed down-regulation of DEGs associated with oxalate decarboxylase activity in the present study could be part of the process of maintaining intracellular ionic balance under water stress as mediated by organic acid concentrations.

## Conclusion

In comparing the performance of three RNAseq analysis tools, both individually and in combination, we have identified differences in terms of the range of DEGs identified and, in some cases, the inferences one might make in terms of the associated biological processes indicated by GO terms. In future work, it is likely that we will continue to use all 3 programmes, J1 and J3 and compare the performance profiles across a more diverse selection of germplasm to see if the most consistent and complete descriptions of transcriptome responses to diminishing water availability are generated by a single, or combinations of analysis methods. While this is not a conservative approach, relative levels of differential gene expression are not, in themselves, an experimental end but more a means with which sets of genes, post-translational products and broader biological processes can be identified for further experimentation using other approaches such as QTL analysis, proteomics, metabolomics and genetic modification.

Major differences were found in terms of the number of enriched GO terms at different stages depending on whether the gene model set was taken from up- or down-regulated DEGs, particularly for the leaf transcriptomes. For the leaf, up-regulated DEGs were associated with more enriched GO terms during the Early comparison stage and gene annotations indicated that many of these may have had transcription factor and membrane transporter activity. For all other stages, down-regulated DEGs generated more enriched GO terms with more general metabolic associations. For the root, few DEGs were identified at Early or Middle comparison stages (though more by DESeq2 than the other programmes) and the Late comparison stage was associated with a far larger number of DEGs. Gene annotations indicated that heavy-metal associated membrane transport and down-regulation of cytoskeleton-associated ATPase activities might be significant processes. However, because of lack of effective repetition at the Late comparison stage, these results have to be treated as preliminary. KEGG analysis also indicated that ATPase and lignin biosynthesis-associated peroxidase activities were affected by increasing water-stress.

## Supporting information

S1 Table *L*. *perenne* genome annotation table (BLAST2GO PRO table export.). (XLSX)

S2 Table Read and alignment statistics for the RNAseq replicates at each sampling point. (XLSX)

S3 Table GO terms associated with different expression categories for shoot DEGs. (XLSX)

S4 Table Gene models and annotations associated with enrichment of GO terms for indicated expression categories from leaf and root. (XLSX)

S5 Table GO terms associated with different expression categories for root DEGs using Early, Middle and Late expression comparisons. (XLSX)

S6 Table The number of shoot DEGs and their contribution to enriched GO terms for each analysis method arranged according to 'mirror-image' pairs. (XLSX)

S1 Fig Images of the four replicates of p226/135/16 sampled at each estimated water content (EWC) for RNAseq. (PPTX)

S2 Fig Count distributions derived from the leaf transcriptomes. DESeq2 rlog transformations plotted against density for each of the four replicates at each sampling point.
(PPTX)

S3 Fig Count distributions derived from the root transcriptomes. DESeq2 rlog transformations plotted against density for each of the four replicates at each sampling point. Note the variation among root-derived samples, particularly at 1% EWC, compared to the leaf-derived samples.
(PPTX)

S4 Fig GO-terms associated with leaf gene expression category comparisons. (PPTX)

S5 Fig GO-terms associated with root gene expression category comparisons. (PPTX)

S1 Methods R script codes implementing the pre-processing and generation of count matrices. (PDF)

S2 Methods R script code for the identification of DEGs using DESeq2. (PDF)

S3 Methods R script code for the identification of DEGs using edgeR. (PDF)

S4 Methods R script code for the identification of DEGs using limma-voom. (PDF)

S1 Results Description of enriched GO terms and associated gene models in Groups 5–8 of the leaf transcriptome. (DOCX)

S2 Results Enriched GO terms identified when including the Late comparisons for the root transcriptome. (DOCX)

S1 Discussion AR and TC comparisons. (DOCX)
