# Supplementary material for: Differential gene expression and gene ontologies associated with increasing water-stress in leaf and root transcriptomes of perennial ryegrass (Lolium perenne)
Source: PLoS One. 2019 Jul 30;14(7):e0220518. doi: 10.1371/journal.pone.0220518 (PMC6667212; doi:10.1371/journal.pone.0220518)
Supplement: S1 Fig — (PPTX) [file pone.0220518.s007.pptx]

## Slide 1
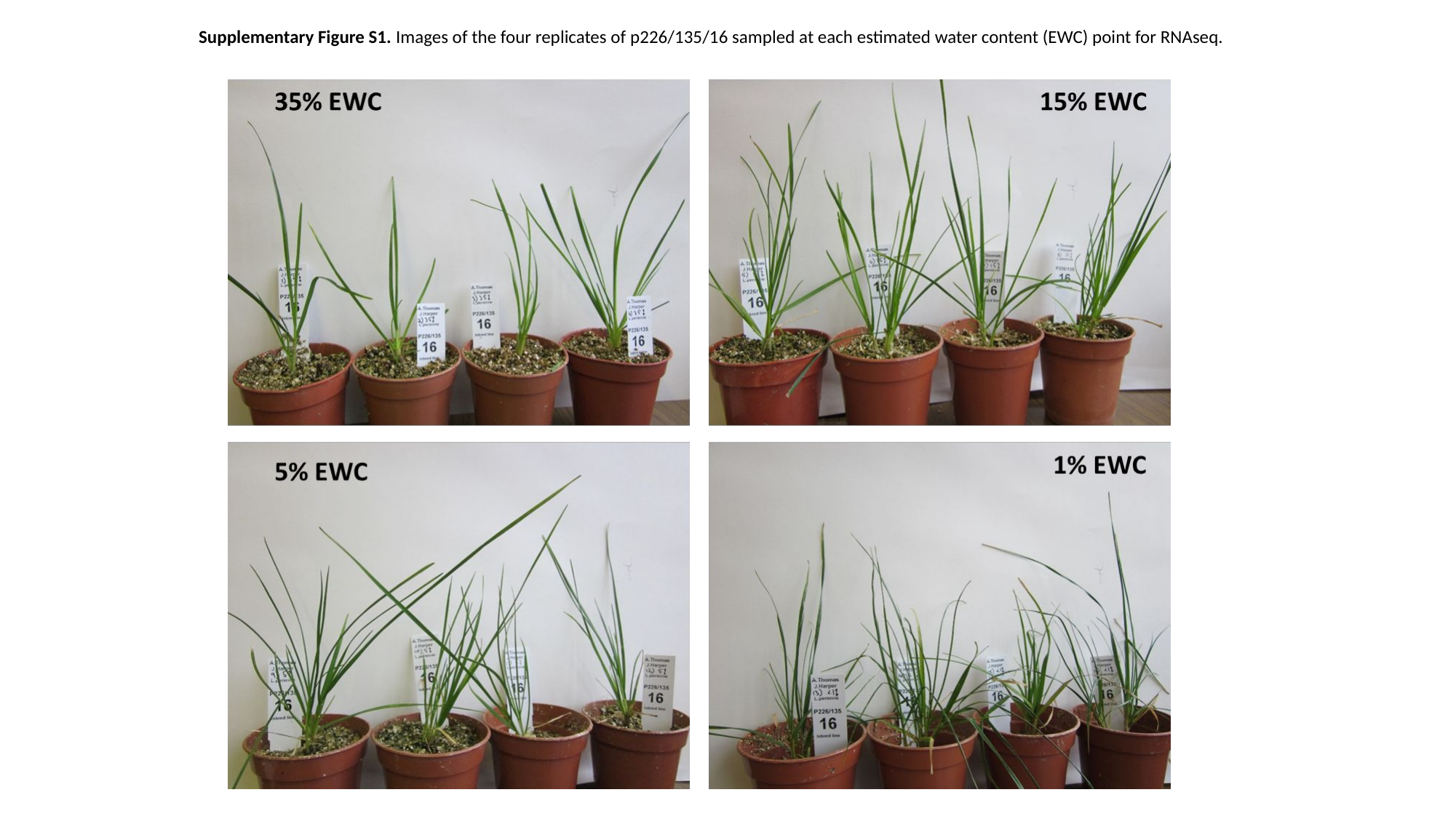

Supplementary Figure S1. Images of the four replicates of p226/135/16 sampled at each estimated water content (EWC) point for RNAseq.
