# Supplementary material for: Differential gene expression and gene ontologies associated with increasing water-stress in leaf and root transcriptomes of perennial ryegrass (Lolium perenne)
Source: PLoS One. 2019 Jul 30;14(7):e0220518. doi: 10.1371/journal.pone.0220518 (PMC6667212; doi:10.1371/journal.pone.0220518)

## Slide 1
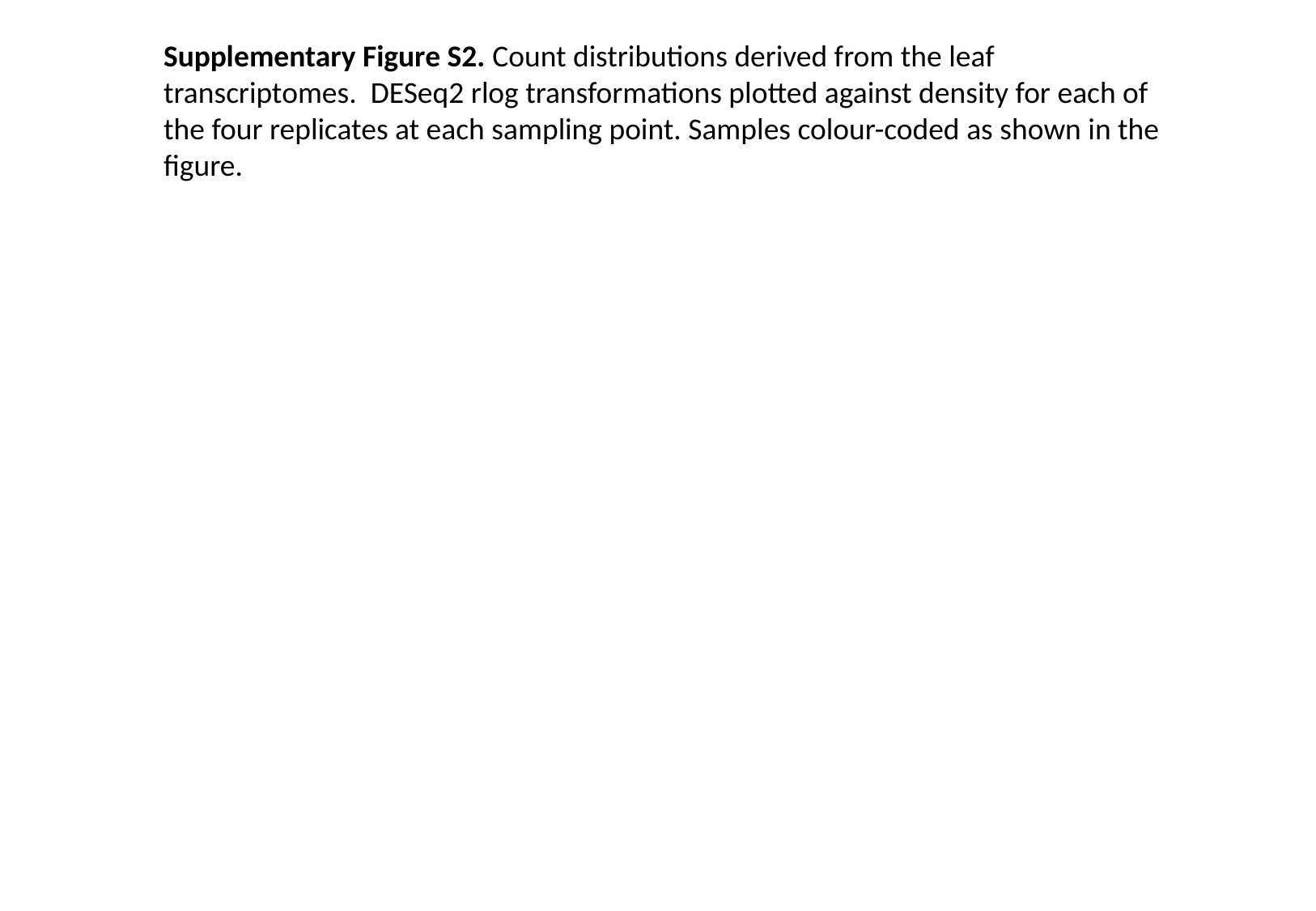

Supplementary Figure S2. Count distributions derived from the leaf transcriptomes. DESeq2 rlog transformations plotted against density for each of the four replicates at each sampling point. Samples colour-coded as shown in the figure.

## Slide 2
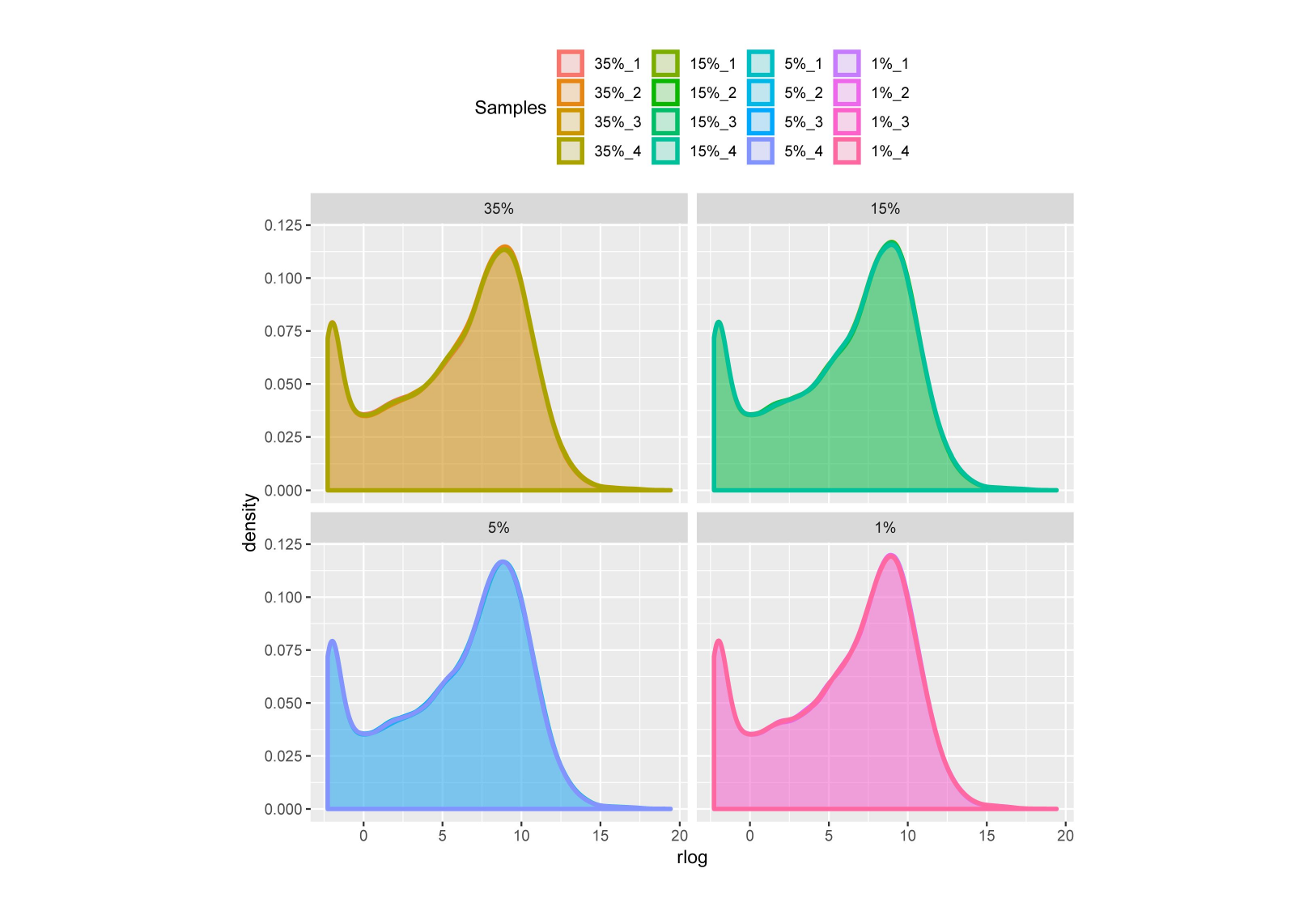

Supplement: S2 Fig — DESeq2 rlog transformations plotted against density for each of the four replicates at each sampling point. (PPTX) [file pone.0220518.s008.pptx]
