# Supplementary material for: Differential gene expression and gene ontologies associated with increasing water-stress in leaf and root transcriptomes of perennial ryegrass (Lolium perenne)
Source: PLoS One. 2019 Jul 30;14(7):e0220518. doi: 10.1371/journal.pone.0220518 (PMC6667212; doi:10.1371/journal.pone.0220518)

## Slide 1
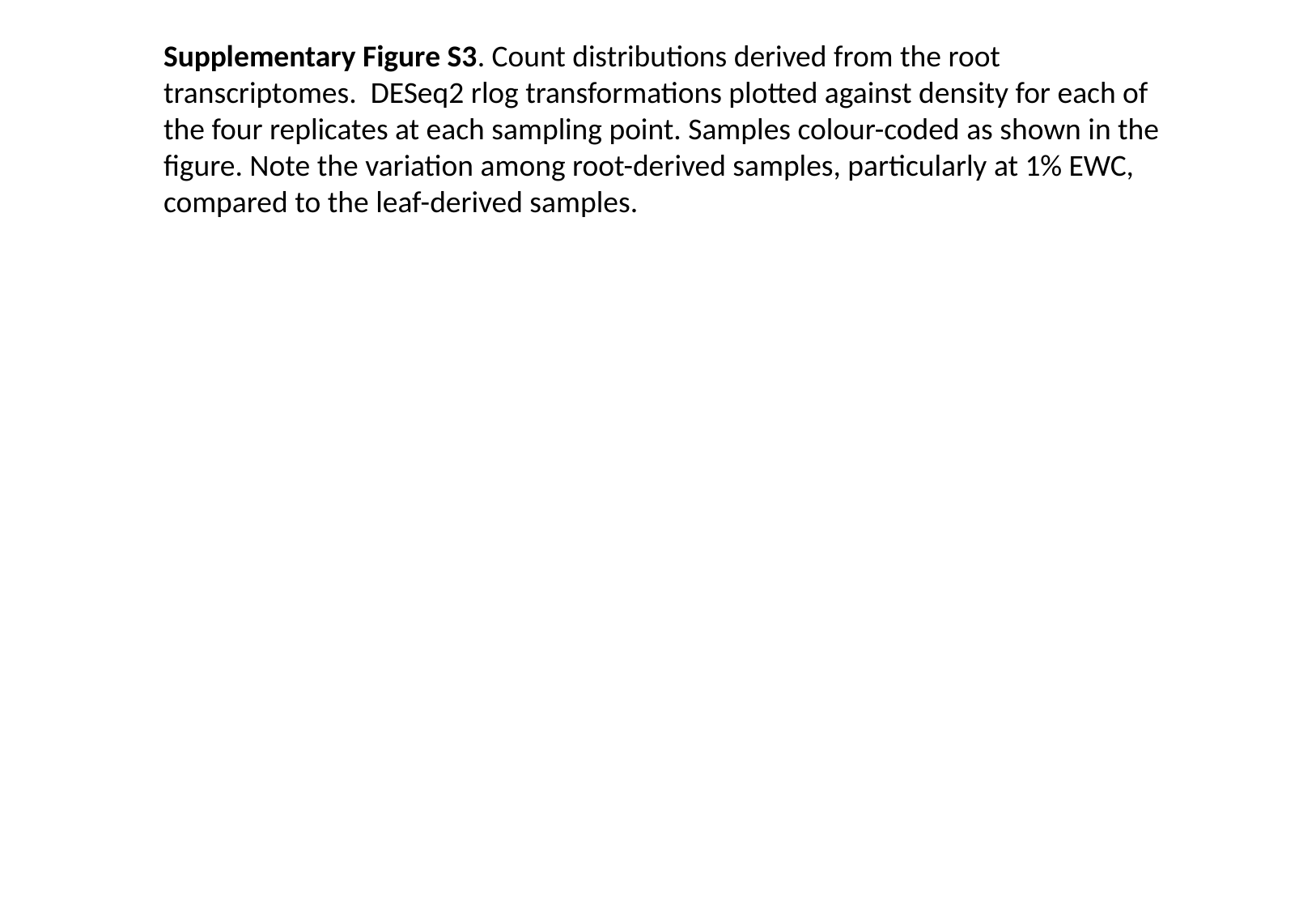

Supplementary Figure S3. Count distributions derived from the root transcriptomes. DESeq2 rlog transformations plotted against density for each of the four replicates at each sampling point. Samples colour-coded as shown in the figure. Note the variation among root-derived samples, particularly at 1% EWC, compared to the leaf-derived samples.

## Slide 2
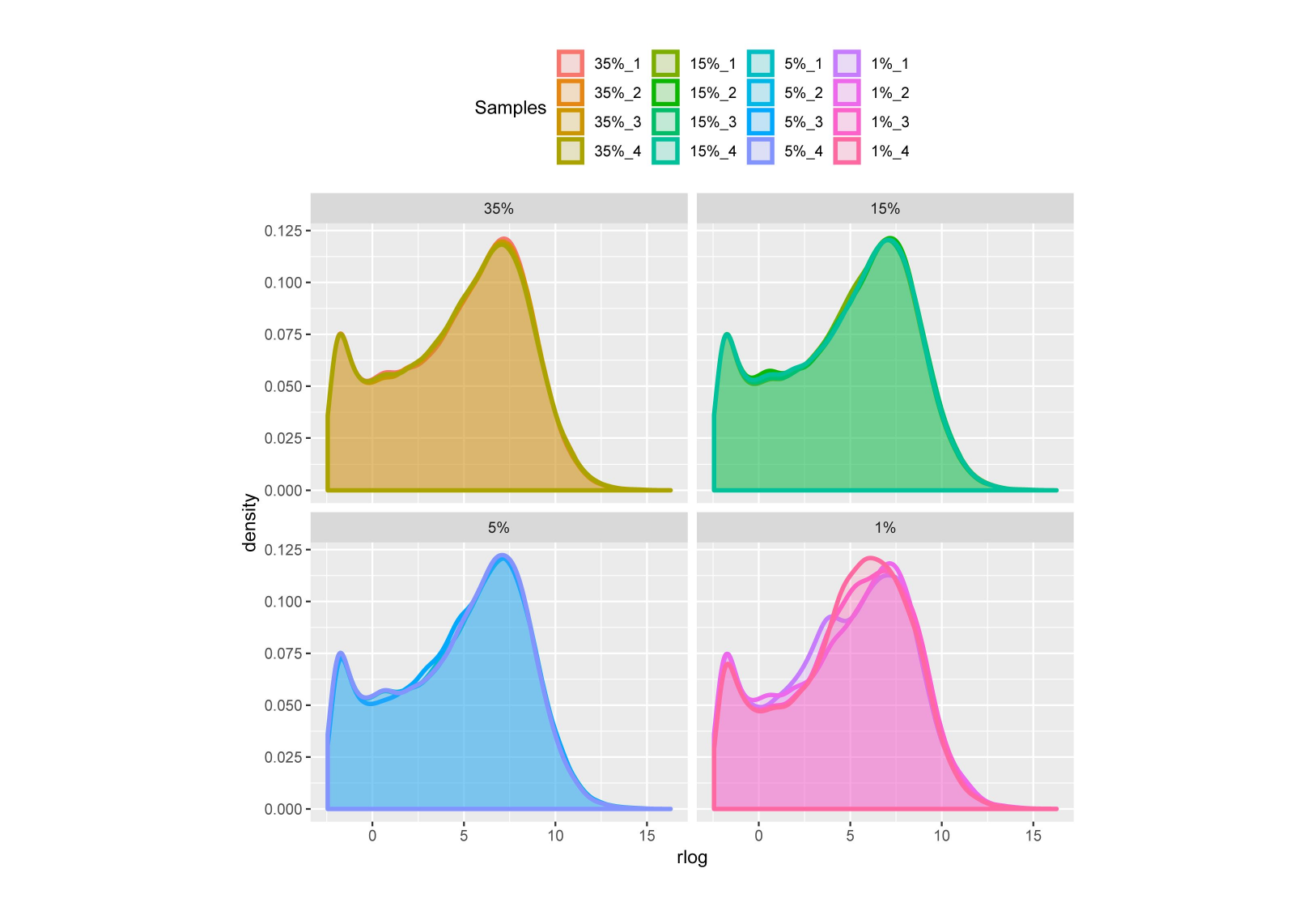

Supplement: S3 Fig — DESeq2 rlog transformations plotted against density for each of the four replicates at each sampling point. Note the variation among root-derived samples, particularly at 1% EWC, compared to the leaf-derived samples. (PPTX) [file pone.0220518.s009.pptx]
