# Supplementary material for: Differential gene expression and gene ontologies associated with increasing water-stress in leaf and root transcriptomes of perennial ryegrass (Lolium perenne)
Source: PLoS One. 2019 Jul 30;14(7):e0220518. doi: 10.1371/journal.pone.0220518 (PMC6667212; doi:10.1371/journal.pone.0220518)
Supplement: S4 Fig — (PPTX) [file pone.0220518.s010.pptx]

## Slide 1
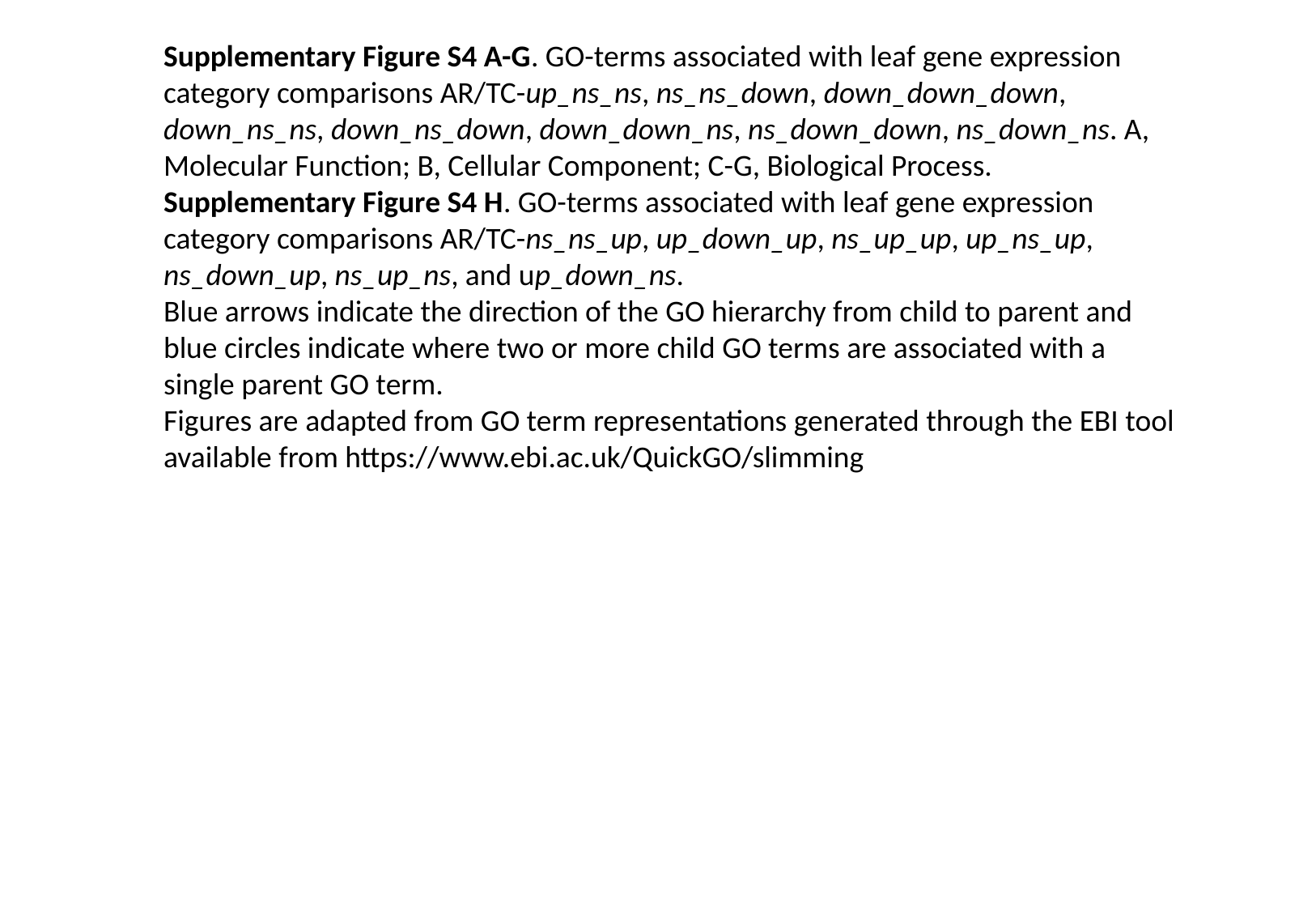

Supplementary Figure S4 A-G. GO-terms associated with leaf gene expression category comparisons AR/TC-up_ns_ns, ns_ns_down, down_down_down, down_ns_ns, down_ns_down, down_down_ns, ns_down_down, ns_down_ns. A, Molecular Function; B, Cellular Component; C-G, Biological Process.
Supplementary Figure S4 H. GO-terms associated with leaf gene expression category comparisons AR/TC-ns_ns_up, up_down_up, ns_up_up, up_ns_up, ns_down_up, ns_up_ns, and up_down_ns.
Blue arrows indicate the direction of the GO hierarchy from child to parent and blue circles indicate where two or more child GO terms are associated with a single parent GO term.
Figures are adapted from GO term representations generated through the EBI tool available from https://www.ebi.ac.uk/QuickGO/slimming

## Slide 2
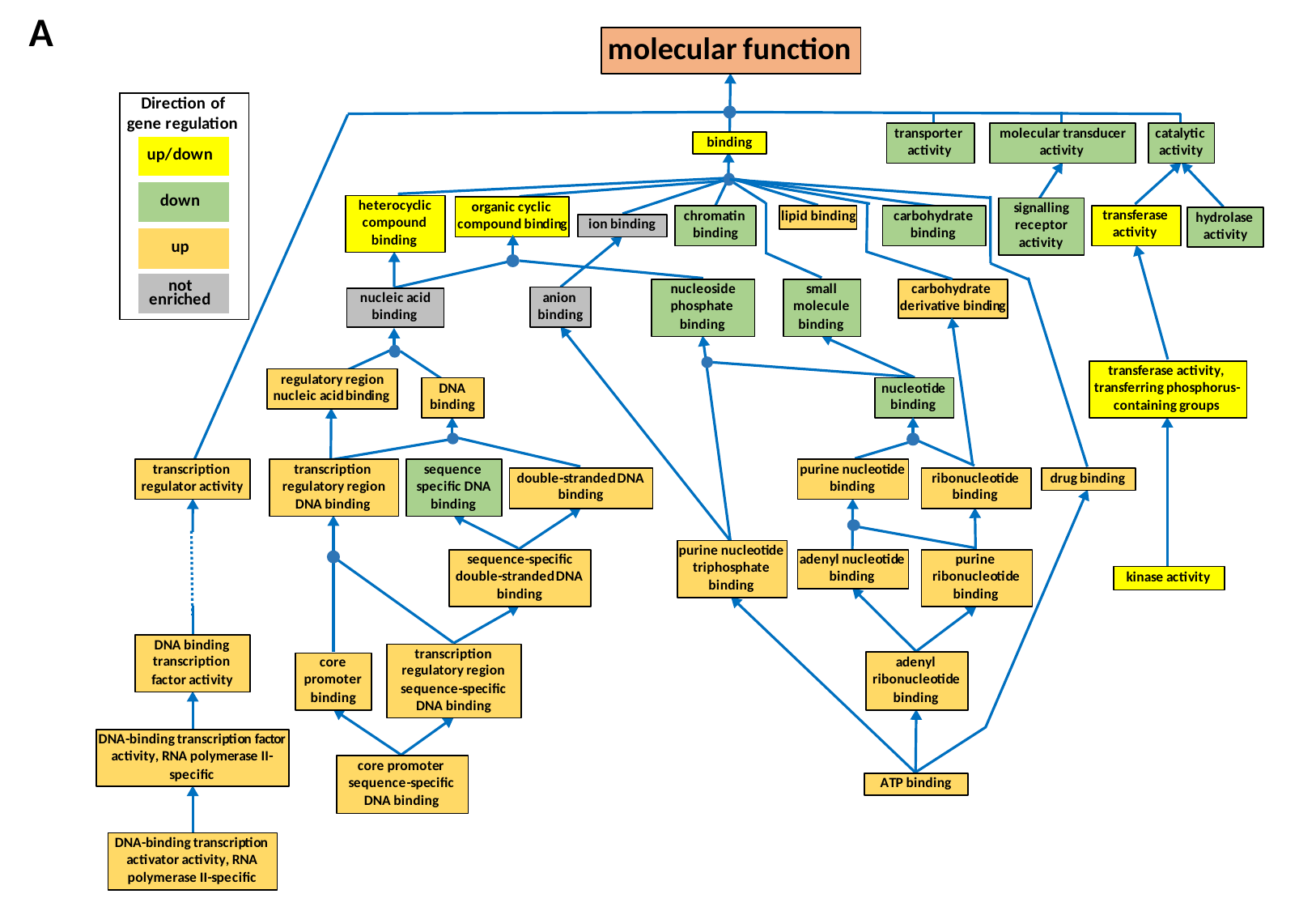

A

## Slide 3
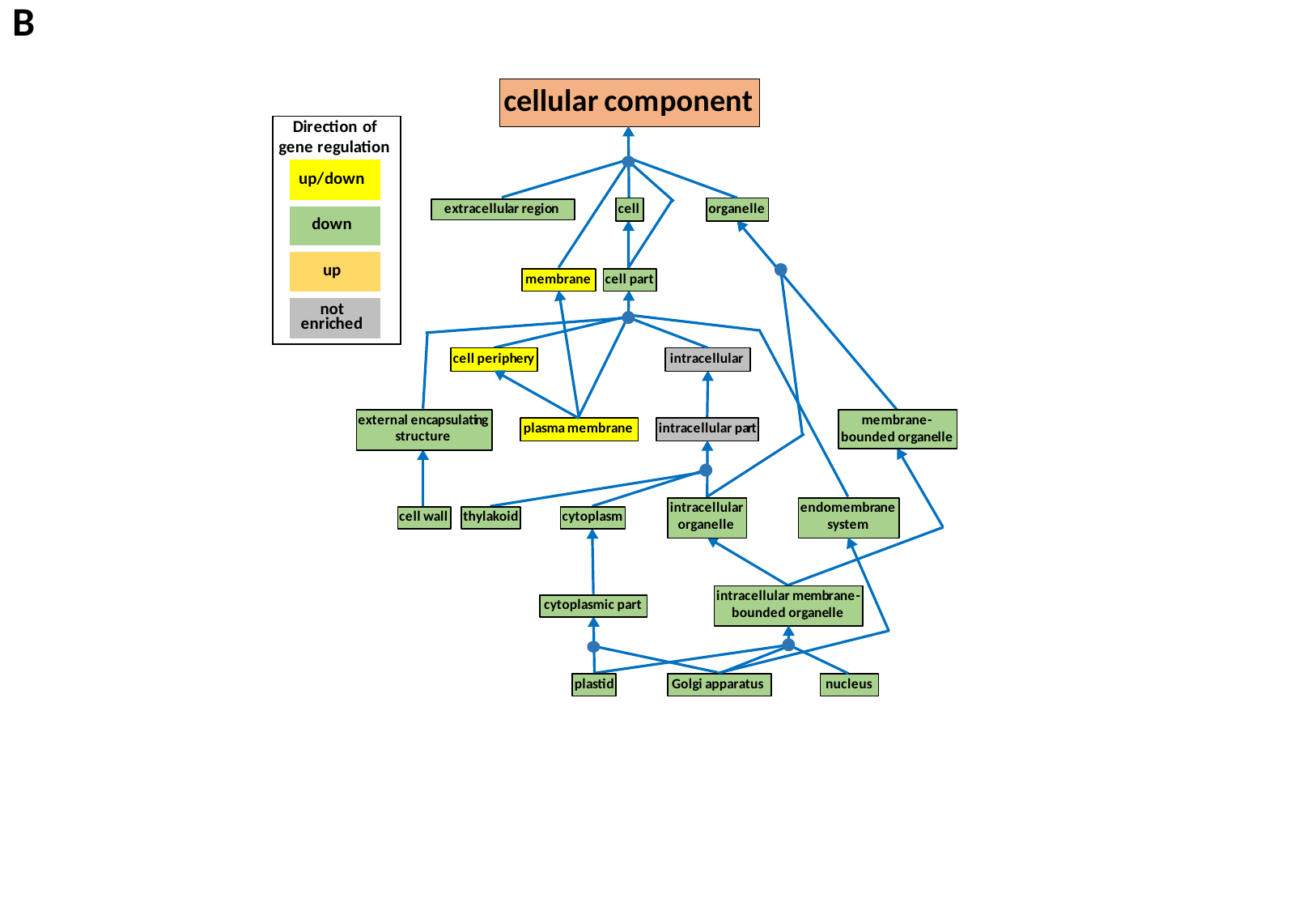

B

## Slide 4
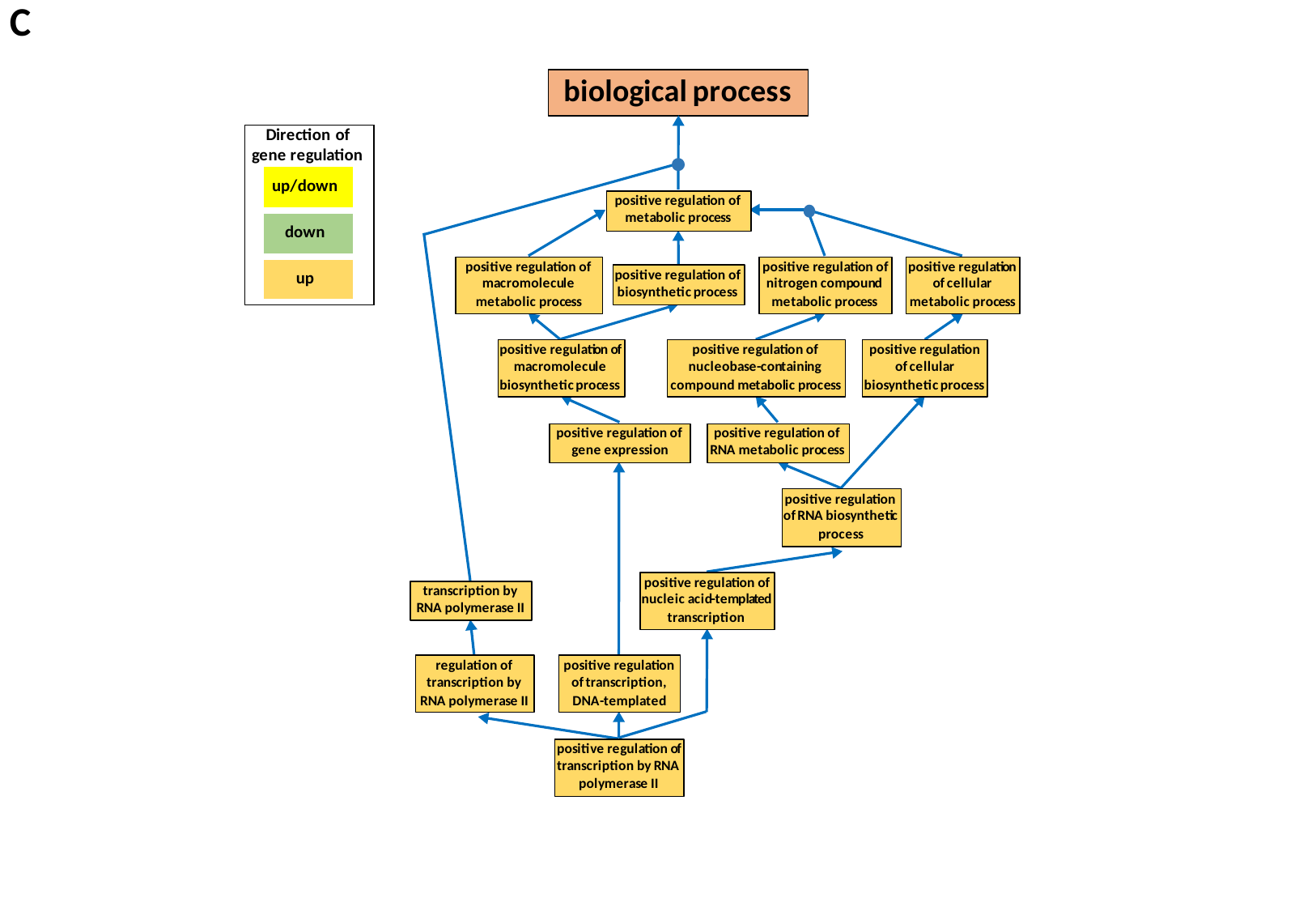

C

## Slide 5
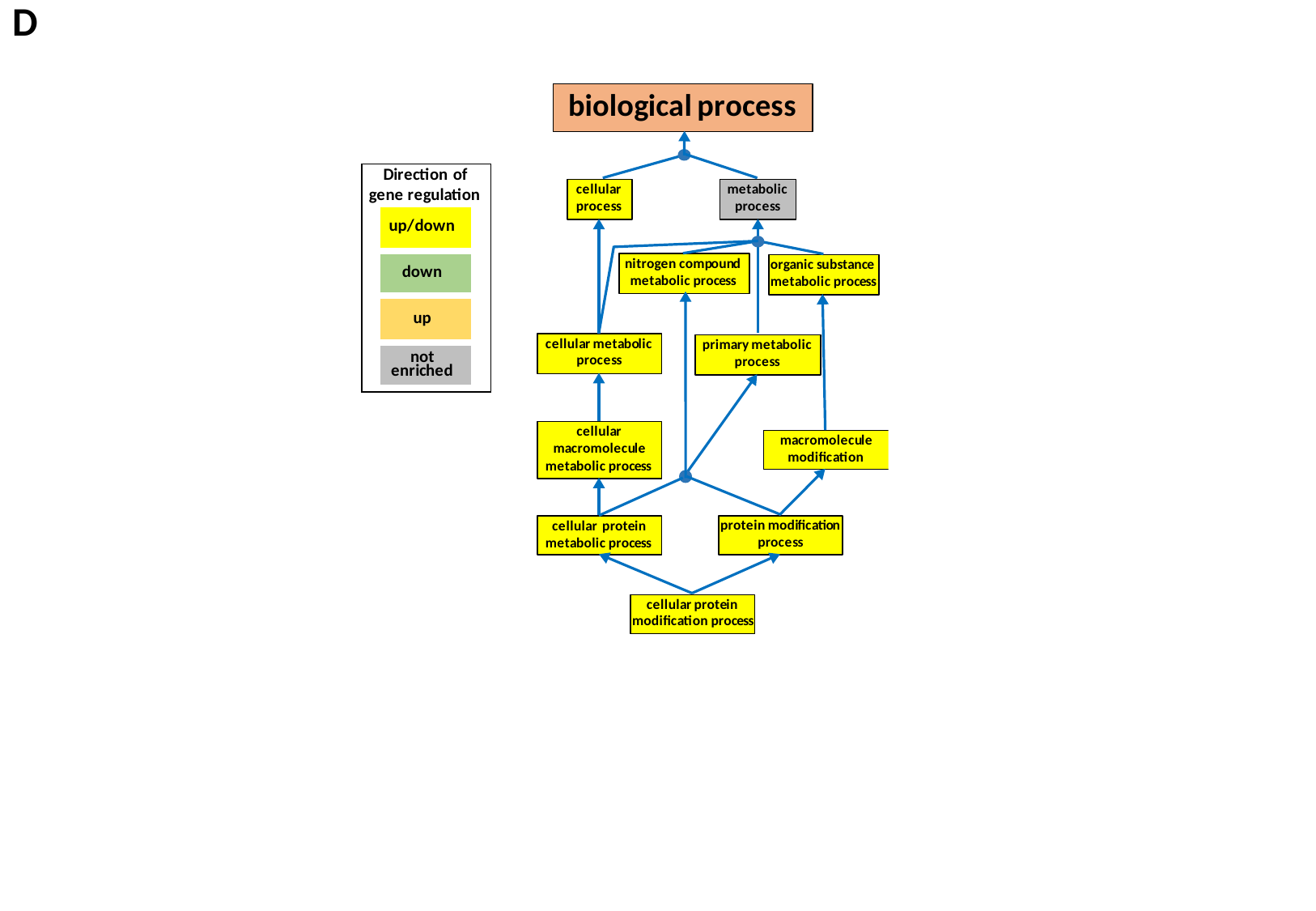

D

## Slide 6
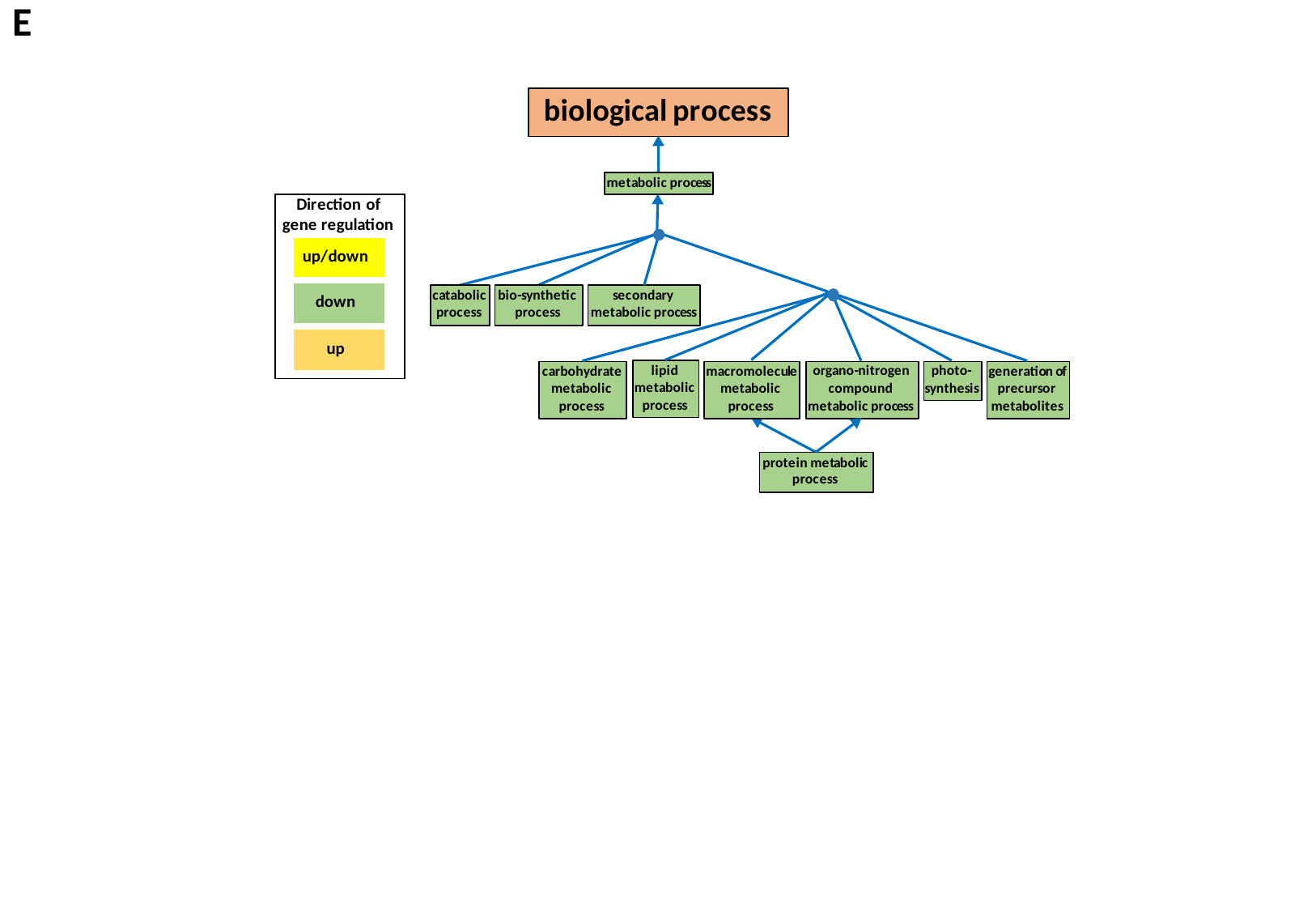

E

## Slide 7
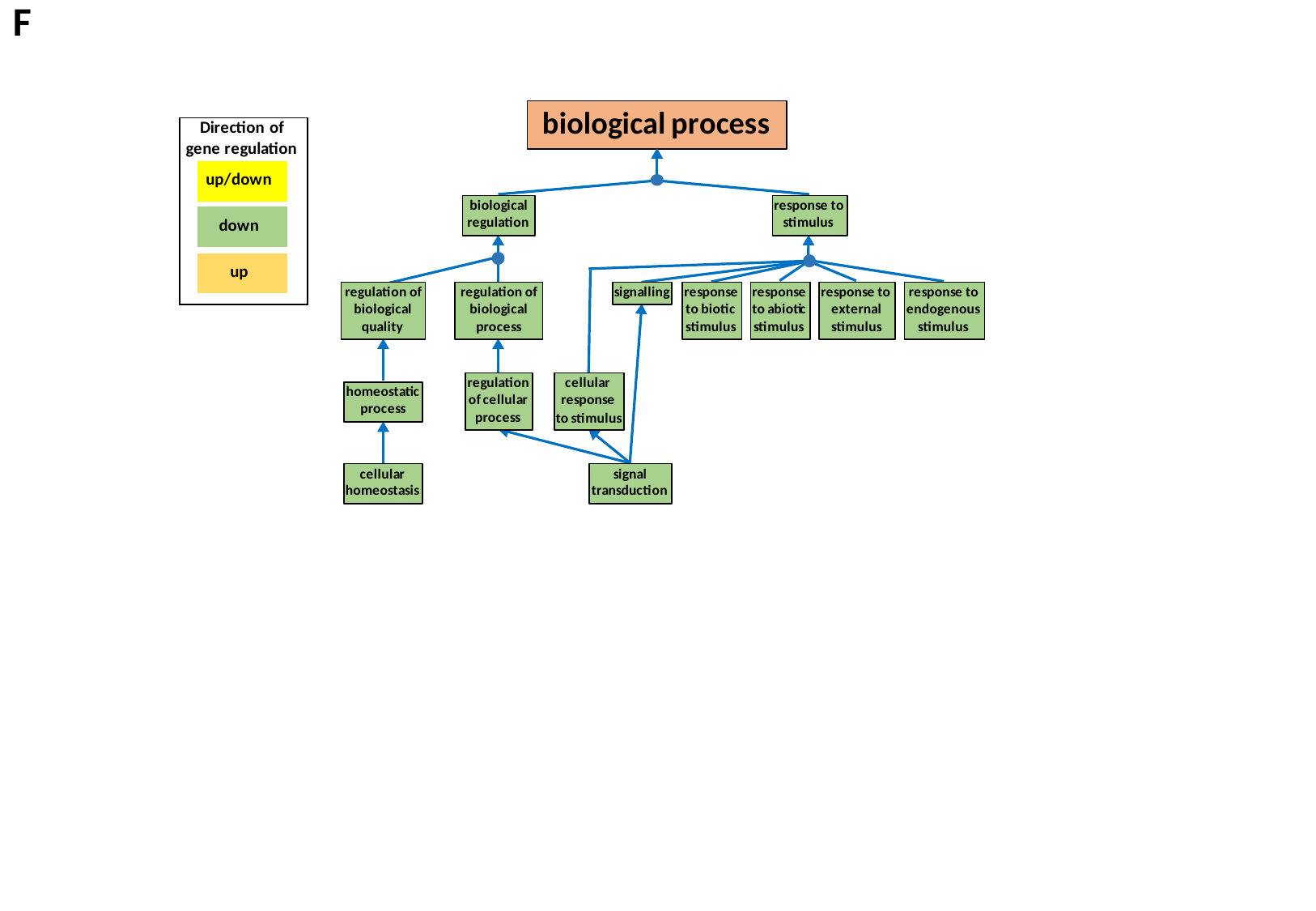

F

## Slide 8
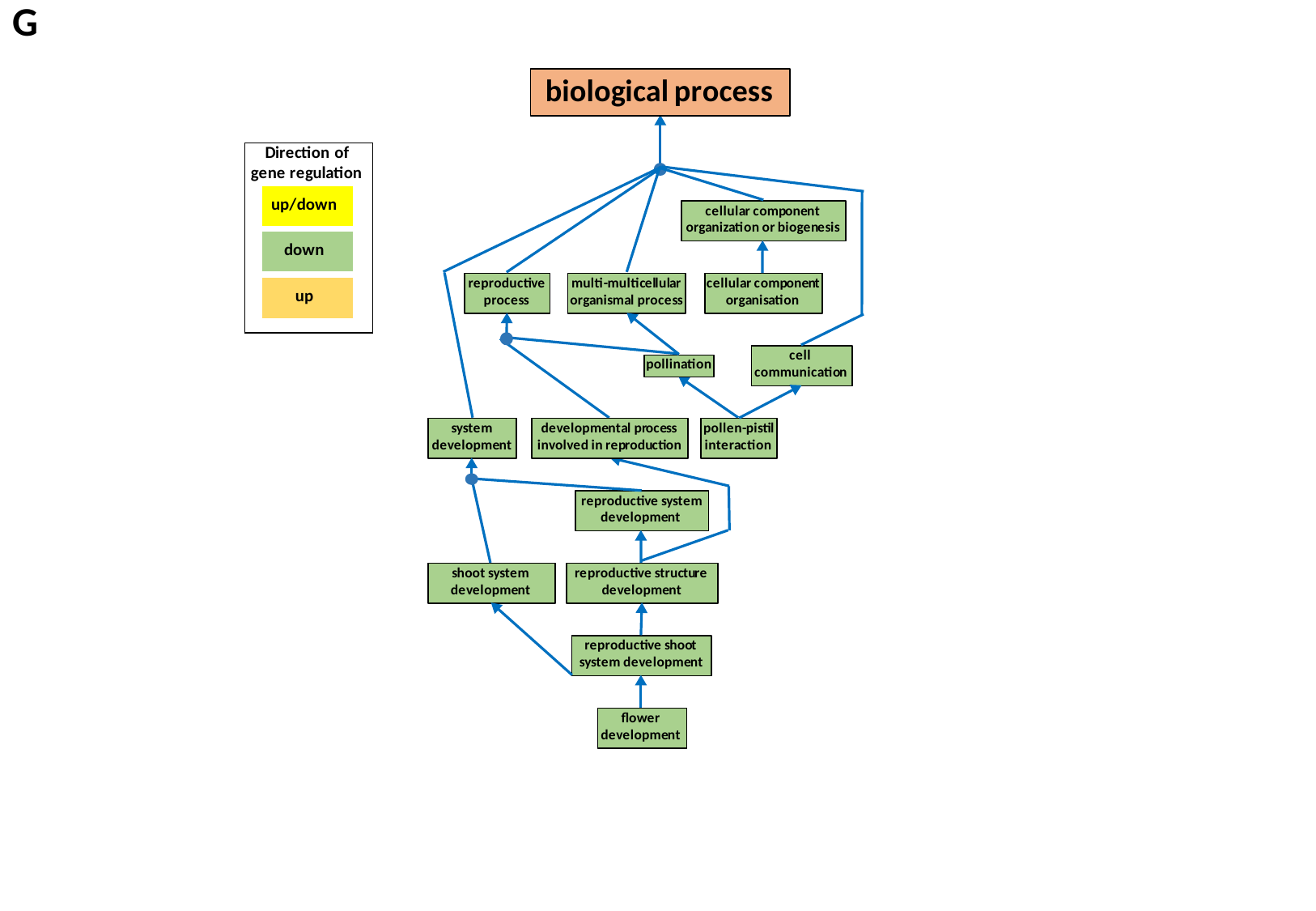

G

## Slide 9
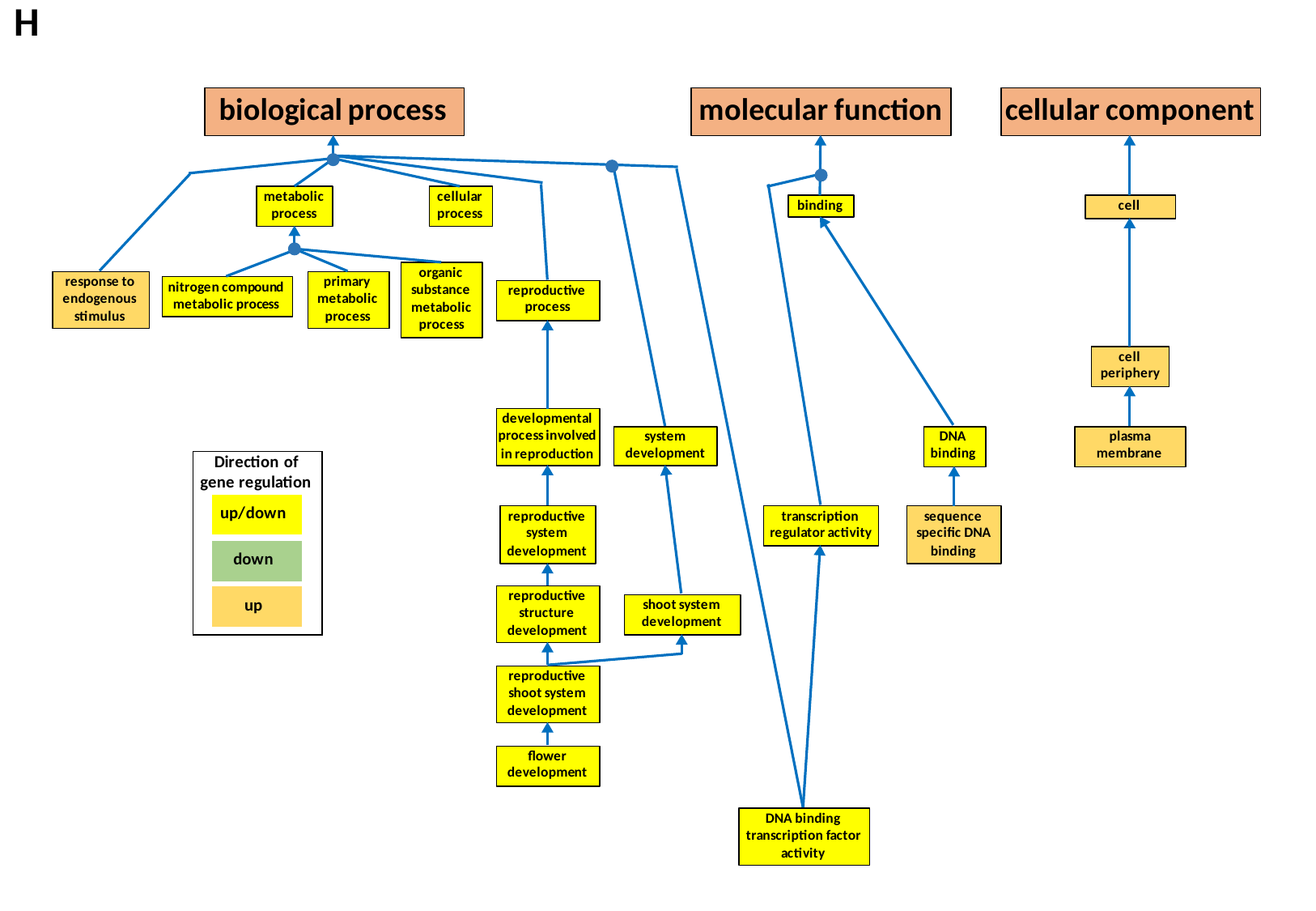

H
