# Supplementary material for: Differential gene expression and gene ontologies associated with increasing water-stress in leaf and root transcriptomes of perennial ryegrass (Lolium perenne)
Source: PLoS One. 2019 Jul 30;14(7):e0220518. doi: 10.1371/journal.pone.0220518 (PMC6667212; doi:10.1371/journal.pone.0220518)
Supplement: S5 Fig — (PPTX) [file pone.0220518.s011.pptx]

## Slide 1
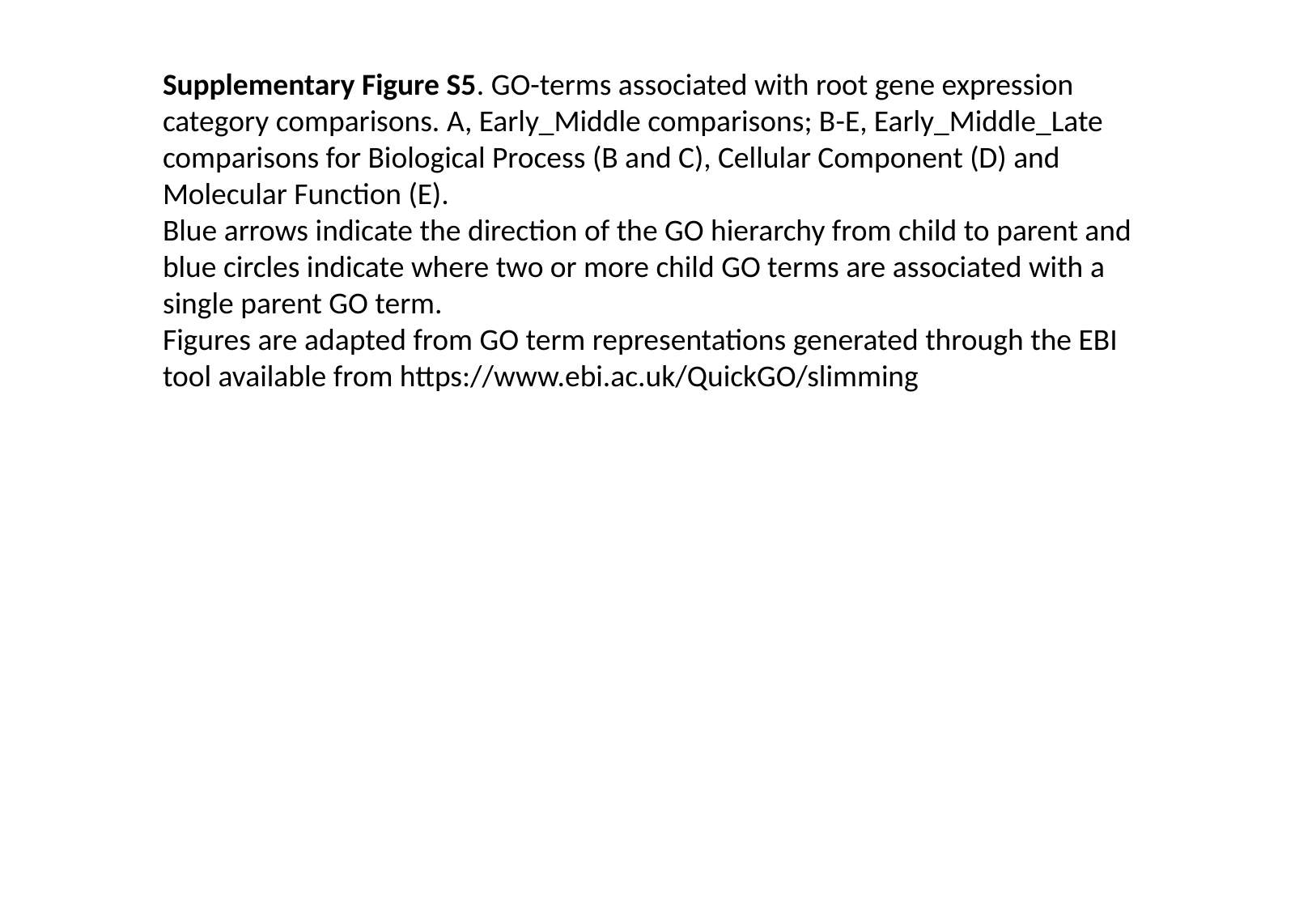

Supplementary Figure S5. GO-terms associated with root gene expression category comparisons. A, Early_Middle comparisons; B-E, Early_Middle_Late comparisons for Biological Process (B and C), Cellular Component (D) and Molecular Function (E).
Blue arrows indicate the direction of the GO hierarchy from child to parent and blue circles indicate where two or more child GO terms are associated with a single parent GO term.
Figures are adapted from GO term representations generated through the EBI tool available from https://www.ebi.ac.uk/QuickGO/slimming

## Slide 2
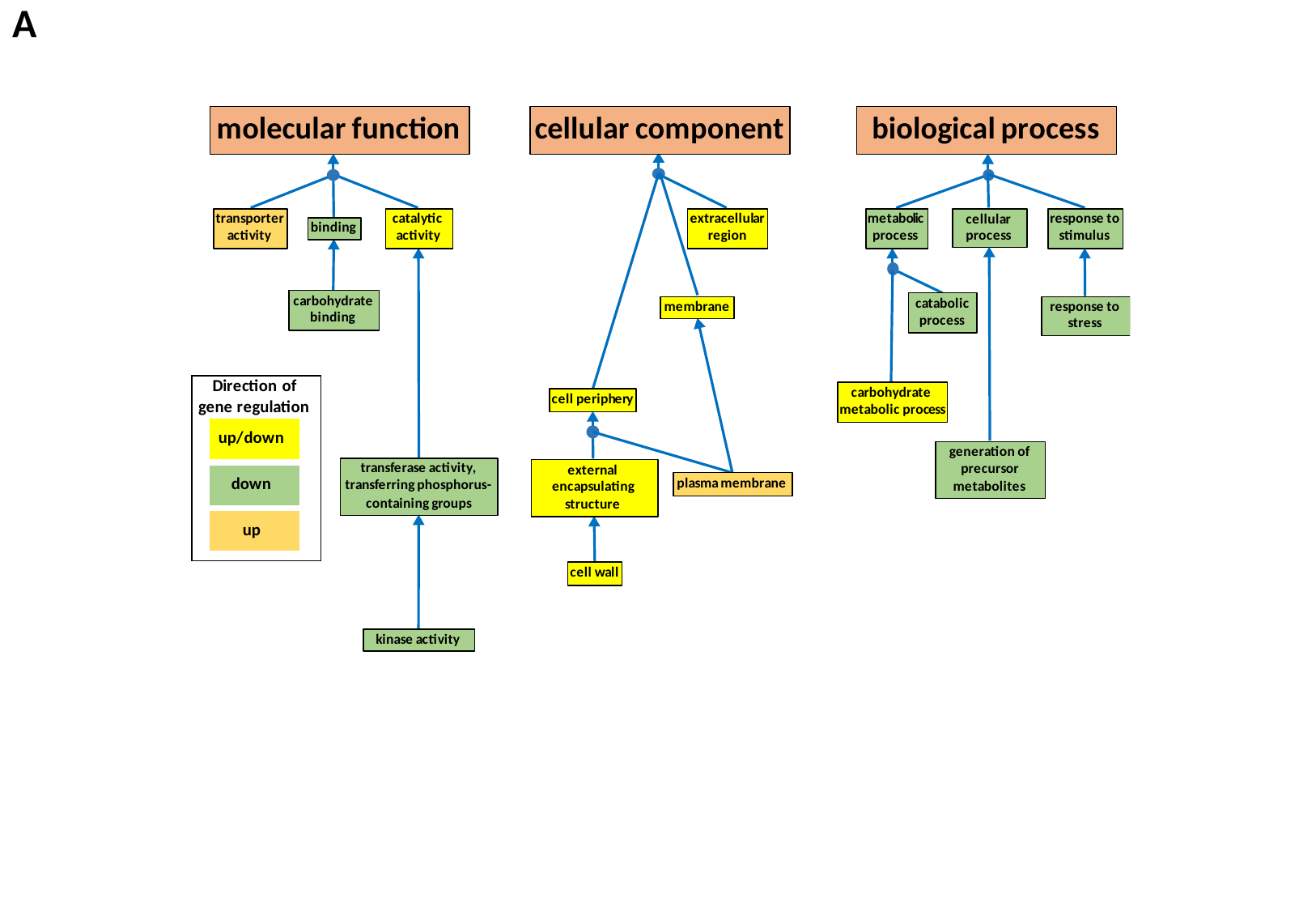

A

## Slide 3
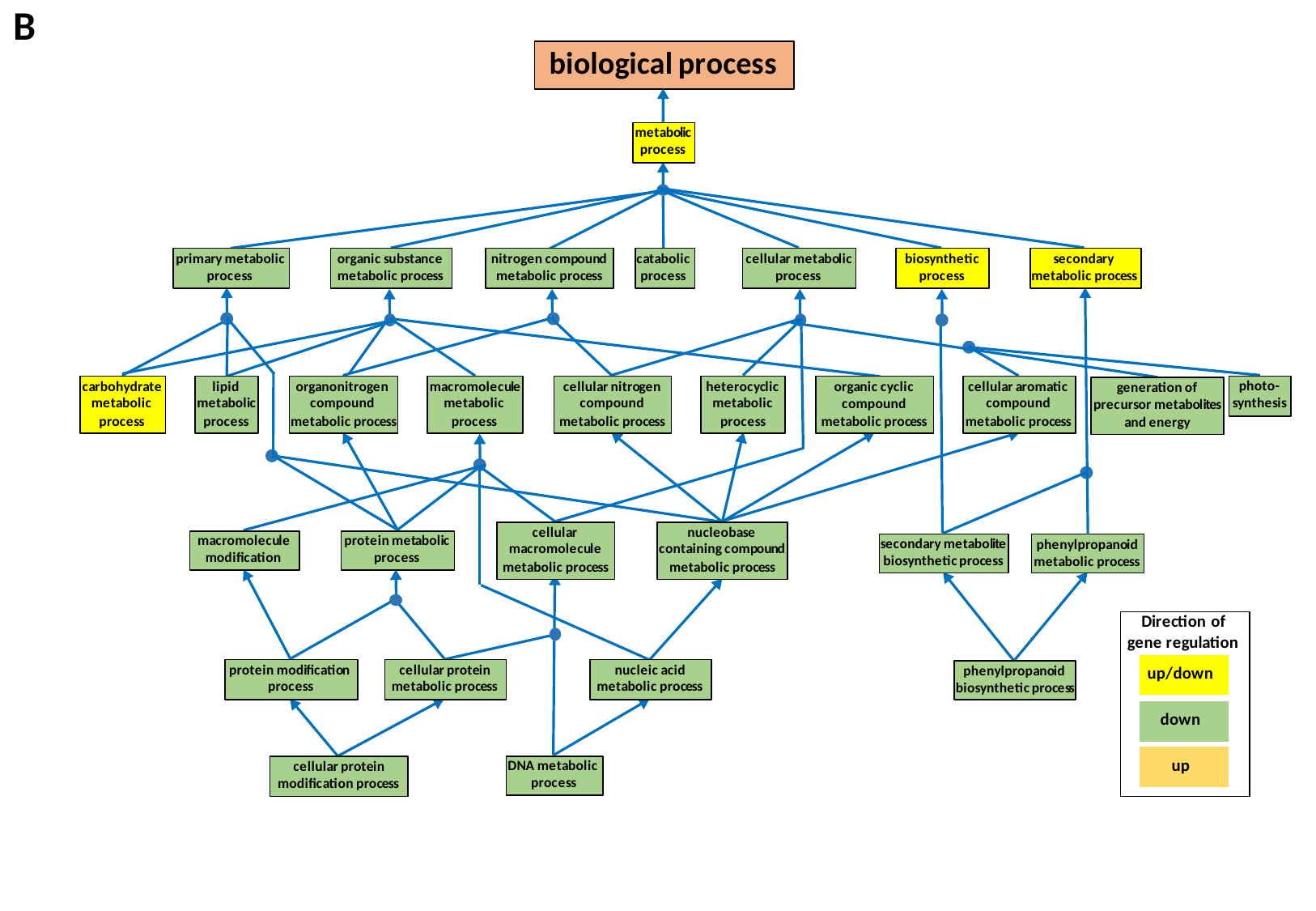

B

## Slide 4
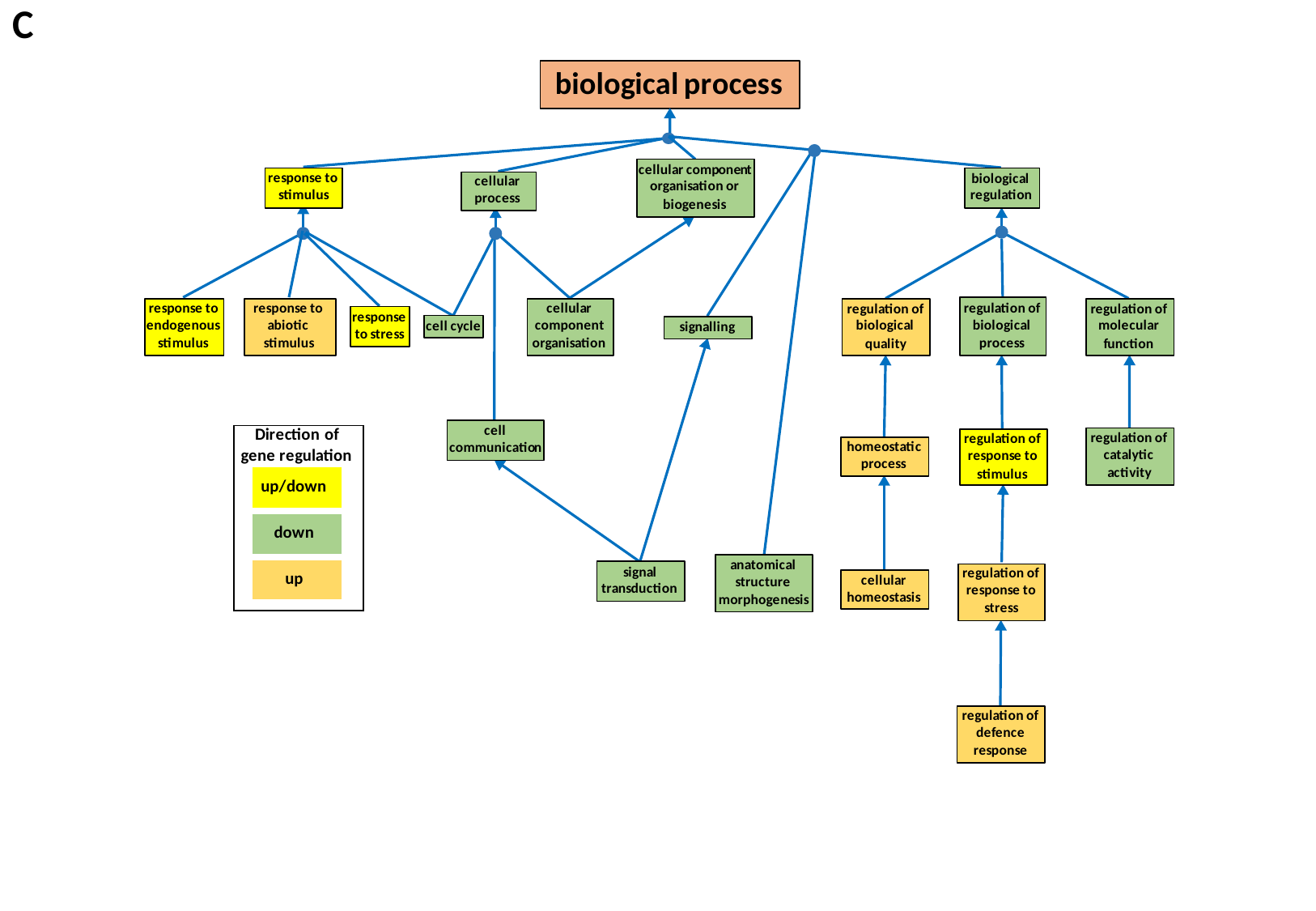

C

## Slide 5
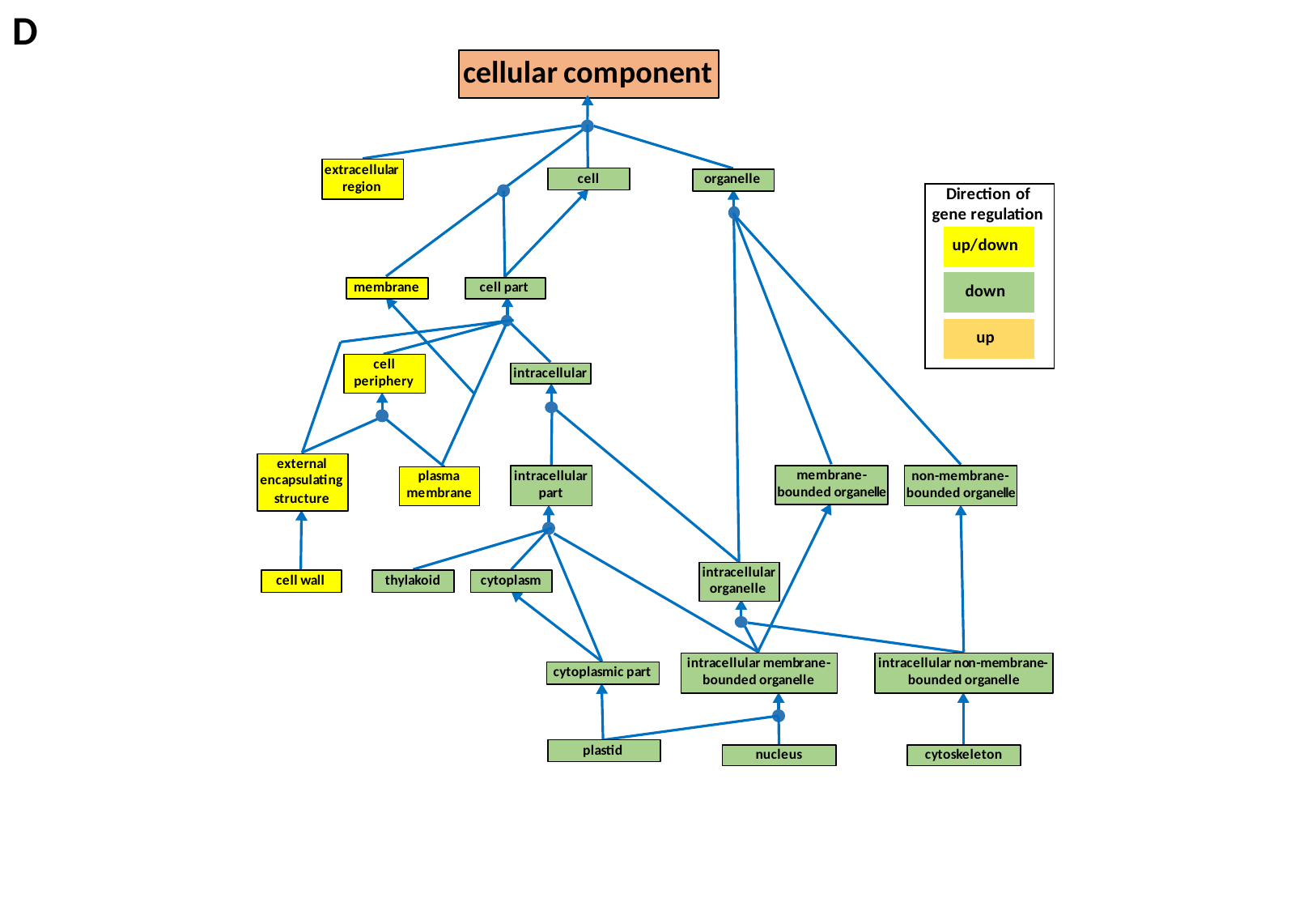

D

## Slide 6
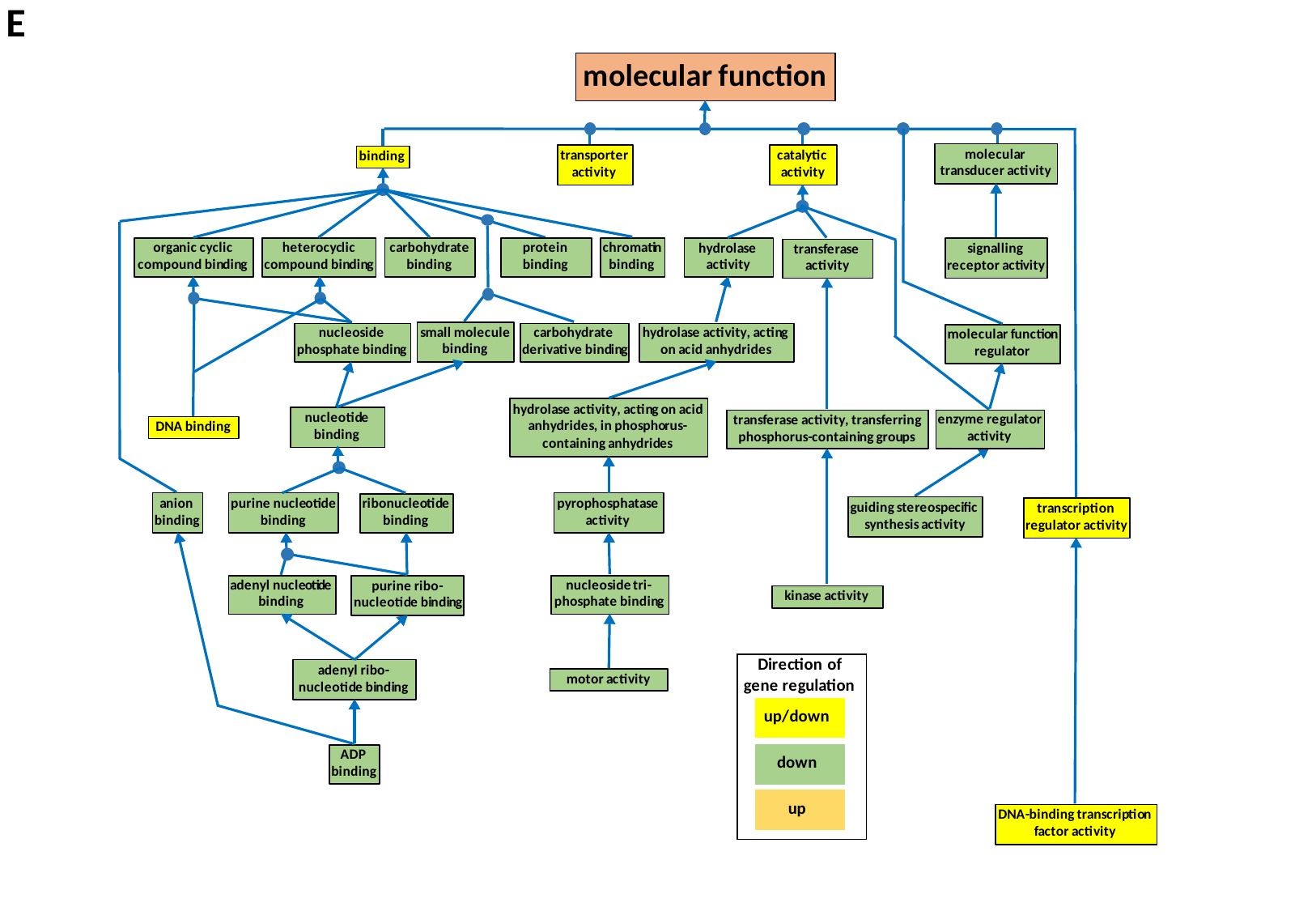

E
