# Supplementary material for: Differential gene expression and gene ontologies associated with increasing water-stress in leaf and root transcriptomes of perennial ryegrass (Lolium perenne)
Source: PLoS One. 2019 Jul 30;14(7):e0220518. doi: 10.1371/journal.pone.0220518 (PMC6667212; doi:10.1371/journal.pone.0220518)
Supplement: S1 Methods — (PDF) [file pone.0220518.s012.pdf]

Pre-processing analysis

Pipeline developer: Albert Fradera Sola

31 March, 2019

Summary

Introduction **1**

Librarys: **1**

Leaf Samples **2**

Independent filtering: 2

Data imput: 2

Extracting the phenotype manifest: 2

Extracting and filtering the count matrices: 2

Multivariate visualization and ordination: 2

Rlog Transformation: 2

Box and density plots: 3

Principal Components Analysis: 3

Heat map of sample distances: 4

Root Samples **5**

Data imput: 5

Figures: **5**

Introduction

This document includes the pre-processing code for the analysis of both leaf and root samples. It also contains the additional plots that result from the analysis and which are not included in the main text.

Librarys:

List of libraries used in this pipeline:

```
library(Biobase)
library(knitr)
library(DESeq2)
library(gplots)
library(ggplot2)
library(RColorBrewer)
library(ggrepel)
library(dplyr)
library(metaMA)
library(xlsx)
library(SummarizedExperiment)
```

```r
library(reshape)
library(pheatmap)
```

Leaf Samples

Independent filtering:

Data imput:

We load the summirized experiment obtained previously (supplementary file S1):

```r
#1-. Load the SE objects:
load("/home/albert/Desktop/Lolium/P226/se_P226.RData")
#2-. Extract leaf samples:
se.f <- se_P226[, se_P226$Tissue == "Leaf"]
```

Extracting the phenotype manifest:

```r
#1-. We extract the phenotype information from the SE object:
pheno <- colData(se.f)
```

Extracting and filtering the count matrices:

```r
#1-. We extract the count matrices from the SE object:
counts <- assay(se.f)
#2-. We change the name of the count matrices:
colnames(counts) <- pheno$SampleName
#3-. We set our independent filtering criteria:
keep <- rowSums(counts)>0
#4-. We apply our independent filtering criteria:
counts.f <- counts[keep,]
```

Multivariate visualization and ordination:

Rlog Transformation:

```r
#1-. We create the statistical model:
dds.model <- DESeqDataSetFromMatrix(counts.f, pheno, design = ~ Soil_Humidity)
#2-.We also relevel the factor soil_huimidity so that 35% is taken as reference level:
dds.model$Soil_Humidity <- relevel(dds.model$Soil_Humidity, "35%")
#3-.We create the DESeq object:
dds <- DESeq(dds.model)
#4-.We apply the rlog transformation:
rld <- rlog(dds)
```

Box and density plots:

We start by creating the box plots from figure 1:

```
#1-. We create a data frame from the rlog data:
dF <- as.data.frame(assay(rld))
#2-. We rename columns in the data frame:
colnames(dF) <- substr(colnames(dF), 6, 10)
colnames(dF)[9:16] <- substr(colnames(dF)[9:16], 2, 5)
#3-. We melt the data frame:
dF <- melt(dF, variable_name = "Samples")
#4-. We create a new data frame based on the conditions:
dF <- data.frame(dF, Condition = substr(dF$Samples, 1, nchar(as.character(dF$Samples))-2))
#5-. We rearrenge the factor leveling:
dF$Condition <- factor(dF$Condition, levels = c("35%", "15%", "5%", "1%"))
#6-. We generate the box plots:
pdf("P226_Leaf_Boxplot.pdf")
ggplot(dF, aes(x = Samples, y = value, fill = Condition))+
  geom_boxplot()+
  xlab("") +
  ylab("rlog transformation")+
  labs(title = "Leaf Tissue Samples Box Plots")+
  theme(axis.text.x = element_text(angle = 60, hjust = 1))
dev.off()
```

Then, we create the density plots:

```
#1-. Using the previous data frame, we create the density plots:
pdf("P226_Leaf_DensityPlot.pdf")
ggplot(dF, aes(x = value, colour = Samples, fill = Samples))+
  geom_density(alpha = 0.2, size = 1.25) +
  facet_wrap(~ Condition) +
  theme(legend.position = "top") +
  xlab(expression("rlog"))
dev.off()
```

Principal Components Analysis:

```
#1-. Row-wise var prior to PCA:
rv <- rowVars(assay(rld))
select <- order(rv, decreasing = TRUE)[seq_len(min(500,length(rv)))]
#2-. Compute PCA
pca <- prcomp(t(assay(rld)[select, ]))
percentVar <- pca$sdev^2/sum(pca$sdev^2)
#3-. Print variance summary:
summary(pca)
#4-. Get scores values of the two first components:
scores <- data.frame(pca$x[,c("PC1","PC2")])
scores$EWC <- as.character(pheno$Soil_Humidity)
scores$EWC[9:16] <- substr(scores$EWC[9:16], 2, nchar(as.character(scores$EWC[9:16])))
scores$EWC <- factor(scores$EWC, levels = c("35%", "15%", "5%", "1%"))
#5-. Labelling samples
D <- as.data.frame(assay(rld))
lab <-substr(colnames(D), 6, 10)
```

```
lab[9:16] <- substr(lab[9:16], 2, nchar(as.character(lab[9:16])))
#6-. Plot scores
pdf("P226_Leaf_PCA.pdf")
ggplot(data=scores,aes(x=PC1, y=PC2, colour=EWC))+
  geom_point(alpha = I(0.7), size=10)+
  geom_text_repel(data = scores,
                  mapping = aes(label = lab),
                  size = 3,
                  fontface = 'bold',
                  color = 'black',
                  box.padding = unit(0.5, "lines"),
                  point.padding = unit(0.5, "lines"))+
  geom_hline(yintercept = 0)+
  geom_vline(xintercept = 0)+
  xlab(paste("PC1: ", round(percentVar[1] * 100), "% variance"))+
  ylab(paste("PC2: ", round(percentVar[2] * 100), "% variance"))+
  labs(title = "Leaf Tissue Principal Component Analysis")+
  scale_fill_manual("EWC",  values =  c("1%" = "#C77CFF",
                                        "5%" = "#7CAE00",
                                        "15%" = "#F8766D",
                                        "35%" = "#00BFC4"))+
  scale_color_manual("EWC",  values =  c("1%" = "#C77CFF",
                                         "5%" = "#7CAE00",
                                         "15%" = "#F8766D",
                                         "35%" = "#00BFC4"))
dev.off()
```

Heat map of sample distances:

```
#1-. Row-wise var prior to sample distances:
rv <- rowVars(assay(rld))
select <- order(rv, decreasing = TRUE)[seq_len(min(500,length(rv)))]
#2-.Labels:
count <-t(assay(rld)[select, ])
#3-.Compute sample distances:
sampleDists <- dist((count), method ="euclidian")
#4-. Transform distances into a matrix:
sampleDistMatrix <- as.matrix(sampleDists)
#5-. Define the colours of the heat map:
colours <- colorRampPalette(rev(brewer.pal(9,"Blues")))(255)
EWC <- substr(rownames(sampleDistMatrix),6,8)
EWC[9:16] <- substr(EWC[9:16], 2, nchar(as.character(EWC[9:16])))
df <- data.frame(EWC)
df$EWC <- factor(df$EWC, levels =c ("35%", "15%", "5%", "1%"))
#6-. Change row and column names:
rownames(sampleDistMatrix) <- rownames(sampleDistMatrix)
rownames(sampleDistMatrix) <- substr(rownames(sampleDistMatrix), 6, 10)
rownames(sampleDistMatrix)[9:16] <- substr(rownames(sampleDistMatrix)[9:16], 2, 5)
colnames(sampleDistMatrix) <- rownames(sampleDistMatrix)
rownames(df) <- rownames(sampleDistMatrix)
#6-. Compute the plot:
pheatmap(sampleDistMatrix,
```

```
         main="Leaf Sample Distances",
         col=colours,
         annotation_col = df,
         cluster_rows = F,
         filename = "P226_Leaf_HeatMap_Distances.pdf",
         width = 7,
         height = 7)
```

Root Samples

We use the same code than the one above for leaf samples, calling for root tissue instead of leaf tissue:

Data imput:

We load the summirized experiment obtained previously (supplementary file S1):

```
#1-. Load the SE objects:
load("/home/albert/Desktop/Lolium/P226/se_P226.RData")
#2-. Extract root samples:
se.f <- se_P226[, se_P226$Tissue == "Root"]
```

We obtain the same plots than for leaf; box plots, which are not included in the main text, are included in figure 2:

Figures:

List of Figures

1 Parallel boxplots from rlog data. 6
2 Parallel boxplots from rlog data. 7

Generation of count matrices

Pipeline developer: Albert Fradera Sola

31 March, 2019

Summary

Create the "Sample Table" **1**

Allocate the .BAM files: **2**

Allocate the .GTF file: **2**

Create a TxDb object: **2**

Compute count matrices: **2**

Create the "Sample Table"

First step is to relate our .BAM files to each sample and make sure each SampleName correlates to its FileName:

```
#1-. Create a vector containg all the samples:
SampleName <- c("Root_35%_1", "Root_35%_2", "Root_35%_3", "Root_35%_4",
                "Root_15%_1", "Root_15%_2", "Root_15%_3", "Root_15%_4",
                "Root_05%_1", "Root_05%_2", "Root_05%_3", "Root_05%_4",
                "Root_01%_1", "Root_01%_2", "Root_01%_3", "Root_01%_4",
                "Leaf_35%_1", "Leaf_35%_2", "Leaf_35%_3", "Leaf_35%_4",
                "Leaf_15%_1", "Leaf_15%_2", "Leaf_15%_3", "Leaf_15%_4",
                "Leaf_05%_1", "Leaf_05%_2", "Leaf_05%_3", "Leaf_05%_4",
                "Leaf_01%_1", "Leaf_01%_2", "Leaf_01%_3", "Leaf_01%_4")
#2-. Create a vector containg all the file names:
FileName <- c("P226_1R.mapped.cleaned.sorted.bam", "P226_2R.mapped.cleaned.sorted.bam",
              "P226_3R.mapped.cleaned.sorted.bam", "P226_4R.mapped.cleaned.sorted.bam",
              "P226_5R.mapped.cleaned.sorted.bam", "P226_6R.mapped.cleaned.sorted.bam",
              "P226_7R.mapped.cleaned.sorted.bam", "P226_8R.mapped.cleaned.sorted.bam",
              "P226_9R.mapped.cleaned.sorted.bam", "P226_10R.mapped.cleaned.sorted.bam",
              "P226_11R.mapped.cleaned.sorted.bam", "P226_12R.mapped.cleaned.sorted.bam",
              "P226_13R.mapped.cleaned.sorted.bam", "P226_14R.mapped.cleaned.sorted.bam",
              "P226_15R.mapped.cleaned.sorted.bam", "P226_16R.mapped.cleaned.sorted.bam",
              "P226_1L.mapped.cleaned.sorted.bam", "P226_2L.mapped.cleaned.sorted.bam",
              "P226_3L.mapped.cleaned.sorted.bam", "P226_4L.mapped.cleaned.sorted.bam",
              "P226_5L.mapped.cleaned.sorted.bam", "P226_6L.mapped.cleaned.sorted.bam",
              "P226_7L.mapped.cleaned.sorted.bam", "P226_8L.mapped.cleaned.sorted.bam",
              "P226_9L.mapped.cleaned.sorted.bam", "P226_10L.mapped.cleaned.sorted.bam",
              "P226_11L.mapped.cleaned.sorted.bam", "P226_12L.mapped.cleaned.sorted.bam",
              "P226_13L.mapped.cleaned.sorted.bam", "P226_14L.mapped.cleaned.sorted.bam",
              "P226_15L.mapped.cleaned.sorted.bam", "P226_16L.mapped.cleaned.sorted.bam")
#3-. Create a vector containg the tissue type:
```

```r
Tissue <- rep(c("Root","Leaf"), each= 16)
#4-. Create a vector containg the SWC level:
Soil_Humidity <- rep(c("35%","15%","05%", "01%"), each = 4, times =2)
#5-. Create a table containing all the vectors:
sampleTable <- data.frame(SampleName, FileName, Tissue, Soil_Humidity)
#6-. Save the table:
write.csv(sampleTable, file = "sampleTable.csv", row.names = F)
```

Allocate the .BAM files:

We must indicate where our .Bam files can be found in our machine:

```r
#1-. Indicate the path to our files:
extDataDir <- c("C:/Users/Albert/Documents/Master/TFM/RawFiles")
#2-. Pick all the files contained in our sample table from the path:
bamFiles <- file.path(extDataDir, sampleTable$FileName)
```

Allocate the .GTF file:

In a similar way than before, we need to indicate where is our reference genome file in our machine:

```r
#1-. Indicate the path to our files:
annotFile <- "~/Lp_Annotation_V1.1.mp.gff3"
```

Create a TxDb object:

TxDb objects store transcript metadata and are used for creating the count matrices:

```r
#1-. Load the GenomicFeatures library:
library(GenomicFeatures)
#2-. Create the TxDb object using the reference genome:
hse <- makeTxDbFromGFF(file=annotFile, format="gff3")
#3-. Extract your target material (in our case, exons at gene level):
exonsByGene <- exonsBy(hse, by="gene")
```

Compute count matrices:

Finally, we create a summarezied experiment object which contains our count matrices:

```r
#1-. Load the GenomicAlignments library:
library(GenomicAlignments)
#2-. Create the summarized experiment object using the TxDb object and the .BAM files:
se <- summarizeOverlaps(exonsByGene, BamFileList(bamFiles, yieldSize = 2000000),
                        mode = "Union", singleEnd = F, fragments = T)
#3-. Relate data in our summarized experiment object to our sample table:
colData(se) <- DataFrame(sampleTable)
#4-. Reaname columns from our summarized experiment object:
colnames(se) <- sampleTable$SampleName
```

```r
#5-. Save the summarized experiment object:
save(se, file= "se_lolium.RData")
```
