# Supplementary material for: Differential gene expression and gene ontologies associated with increasing water-stress in leaf and root transcriptomes of perennial ryegrass (Lolium perenne)
Source: PLoS One. 2019 Jul 30;14(7):e0220518. doi: 10.1371/journal.pone.0220518 (PMC6667212; doi:10.1371/journal.pone.0220518)
Supplement: S2 Methods — (PDF) [file pone.0220518.s013.pdf]

Supplementary methods S2

DESeq 2 analysis

Pipeline developer: Albert Fradera Sola

31 March, 2019

Summary

Introduction **1**

Pipiline - Leaf samples (against reference) **1**
- Data imput: 1
- 35% vs 15% analysis 1
  - Data imput: 1
  - Extracting the count matrices and the phenotype: 2
  - Independent Filtering: 2
- Model Building: 2
- Data testing: 2
  - 35% vs 15% analysis: 2
  - 35% vs 5% analysis: 3
  - 35% vs 1% analysis: 3
- Data saving: 3

Introduction

The DESeq2 analysis is performed 6 times, one for each tissue/statistical approach. Here, as an example, we show how it is performed with leaf samples against reference. For the other analysis, we follow the same pipeline, adjusting the variables to each wanted condition.

Pipiline - Leaf samples (against reference)

Data imput:

We load the summirized experiment obtained previously (supplementary file 1):

```
#1-. Load the SE objects:
load("se_lolium.RData")
#2-. Extract leaf samples:
se.f <- se[, se$Tissue == "Leaf"]
```

35% vs 15% analysis

Data imput:

We load the summirized experiment obtained previously (supplementary file 1):

```r
#1-. Load the SE objects:
load("/home/albert/Desktop/Lolium/P226/se_P226.RData")
#2-. Extract leaf samples:
se.f <- se_P226[, se_P226$Tissue == "Leaf"]
```

Extracting the count matrices and the phenotype:

```r
#1-. We extract the phenotype contained in the columns
pheno <- colData(se.f)
#2-. We extract the count matrices
counts <- assay(se.f)
#3-. We rename the count matrices accoding to the phenotype
colnames(counts) <- pheno$SampleName
```

Independent Filtering:

```r
#1-. We stablish our independent filtering threshold
keep <- rowSums(counts)>0
#2-. We apply the threshold to our counting matrices
counts.f <- counts[keep,]
```

Model Building:

```r
#1-. We create a dds object:
dds.model <- DESeqDataSetFromMatrix(counts.f, pheno, design = ~ Soil_Humidity)
#2-. We set 35% as our first level:
dds.model$Soil_Humidity <- relevel(dds.model$Soil_Humidity, "35%")
#3-. We estimate the parameters of our model:
dds <- DESeq(dds.model)
```

Data testing:

35% vs 15% analysis:

```r
#1-. We contrast the conditions we want to assess:
res_35vs15 <- results(dds, contrast = c("Soil_Humidity", "15%", "35%"))
#2-. We transform our data into a data.frame:
res.df.1 <- as.data.frame(res_35vs15)
#3-. We get rid off those genes with NA values:
res.df.1 <- res.df.1[complete.cases(res.df.1),]
#4-. We order the results by
res.ord.1 <- res.df.1[order(res.df.1$padj, decreasing = TRUE), ]
#5-. We save the results in a .csv file:
write.csv(file="P226_DESeq2_Results_Leaf_AR_1.csv", x=res.ord.1)
#6-. We save the results in an excel file:
write.xlsx(res.ord.1, file="P226_DESeq2_Results_Leaf_AR_1.xlsx", row.names = TRUE)
```

35% vs 5% analysis:

```
#1-. We contrast the conditions we want to assess:
res_15vs05 <- results(dds, contrast = c("Soil_Humidity", "05%", "35%"))
#2-. We transform our data into a data.frame:
res.df.2 <- as.data.frame(res_15vs05)
#3-. We get rid off those genes with NA values:
res.df.2 <- res.df.2[complete.cases(res.df.2),]
#4-. We order the results by
res.ord.2 <- res.df.2[order(res.df.2$padj, decreasing = TRUE), ]
#5-. We save the results in a .csv file:
write.csv(file="P226_DESeq2_Results_Leaf_AR_2.csv", x=res.ord.2)
#6-. We save the results in an excel file:
write.xlsx(res.ord.2, file="P226_DESeq2_Results_Leaf_AR_2.xlsx", row.names = TRUE)
```

35% vs 1% analysis:

```
#1-. We contrast the conditions we want to assess:
res_05vs01 <- results(dds, contrast = c("Soil_Humidity", "01%", "35%"))
#2-. We transform our data into a data.frame:
res.df.3 <- as.data.frame(res_05vs01)
#3-. We get rid off those genes with NA values:
res.df.3 <- res.df.3[complete.cases(res.df.3),]
#4-. We order the results by
res.ord.3 <- res.df.3[order(res.df.3$padj, decreasing = TRUE), ]
#5-. We save the results in a .csv file:
write.csv(file="P226_DESeq2_Results_Leaf_AR_3.csv", x=res.ord.3)
#6-. We save the results in an excel file:
write.xlsx(res.ord.3, file="P226_DESeq2_Results_Leaf_AR_3.xlsx", row.names = TRUE)
```

Data saving:

```
#1-. We create an .RData object containing all data generated:
save.image(file= "P226_DESeq2_Leaf_AR.RData")
```
