# Supplementary material for: Differential gene expression and gene ontologies associated with increasing water-stress in leaf and root transcriptomes of perennial ryegrass (Lolium perenne)
Source: PLoS One. 2019 Jul 30;14(7):e0220518. doi: 10.1371/journal.pone.0220518 (PMC6667212; doi:10.1371/journal.pone.0220518)
Supplement: S3 Methods — (PDF) [file pone.0220518.s014.pdf]

Supplementary methods S3

edgeR analysis

Pipeline developer: Albert Fradera Sola

31 March, 2019

Summary

Introduction **1**

Pipiline - Leaf samples (against reference) **1**

Data imput: 1
Extracting the count matrices and the phenotype: 1
Independent Filtering: 2
Data normalization: 2
Model Building: 2
Data testing: 2
35% vs 15% analysis: 2
35% vs 5% analysis: 3
35% vs 1% analysis: 3
Data saving: 3

Introduction

The edgeR analysis is performed 6 times, one for each tissue/statistical approach. Here, as an example, we show how it is performed with leaf samples against reference. For the other analysis, we follow the same pipeline, adjusting the variables to each wanted condition.

Pipiline - Leaf samples (against reference)

Data imput:

We load the summirized experiment obtained previously (supplementary file 1)

```
#1-. Load the SE objects:
load("/home/albert/Desktop/Lolium/P226/se_P226.RData")
#2-. Extract leaf samples:
se.f <- se_P226[, se_P226$Tissue == "Leaf"]
```

Extracting the count matrices and the phenotype:

```
#1-. We extract the phenotype contained in the columns
pheno <- colData(se.f)
#2-. We set 35% as our first level:
pheno$Soil_Humidity <- relevel(pheno$Soil_Humidity, "35%")
#3-. We extract the count matrices
counts <- assay(se.f)
```

```r
#4-. We rename the count matrices accoding to the phenotype
colnames(counts) <- pheno$SampleName
```

Independent Filtering:

```r
#1-. We stablish our independent filtering threshold
keep <- rowSums(counts)>0
#2-. We apply the threshold to our counting matrices
counts.f <- counts[keep,]
```

Data normalization:

```r
#1-. We create a dge object:
dge <- DGEList(counts=counts.f, group=pheno$Soil_Humidity)
#2-. We normalize our data using the TMM method:
dge.n <- calcNormFactors(dge, method = "TMM")
```

Model Building:

```r
#1-. We set the desing for our model:
design <- model.matrix(~0+dge.n$samples$group)
#2-. We change the rownames of our design:
rownames(design) <- colnames(dge.n)
#3-. We change the colnames of our design:
colnames(design) <- c("Leaf_35%","Leaf_01%", "Leaf_05%", "Leaf_15%")
#4-. We estimate the dispersion parametes of our data:
dge.p <- estimateDisp(dge.n, design, robust=TRUE)
#5-. We fit our model:
fit <- glmQLFit(dge.p, design, robust=TRUE)
```

Data testing:

35% vs 15% analysis:

```r
#1-. We contrast the conditions we want to assess:
qlf.1 <- glmQLFTest(fit, contrast=c(-1,0,0,1))
#2-. We obtain our genes results:
res_35vs15 <- topTags(qlf.1, n= nrow(qlf.1$table))
#3-. We transform our data into a data.frame:
res.df.1 <- as.data.frame(res_35vs15)
#4-. We get rid off those genes with NA values:
res.df.1 <- res.df.1[complete.cases(res.df.1),]
#5-. We order the results by
res.ord.1 <- res.df.1[order(res.df.1$FDR, decreasing = TRUE), ]
#6-. We save the results in a .csv file:
write.csv(file="P226_edgeR_Results_Leaf_AR_1.csv", x=res.ord.1)
```

```r
#7-. We save the results in an excel file:
write.xlsx(res.ord.1, file="P226_edgeR_Results_Leaf_AR_1.xlsx", row.names = TRUE)
```

35% vs 5% analysis:

```r
#1-. We contrast the conditions we want to assess:
qlf.2 <- glmQLFTest(fit, contrast=c(-1,0,1,0))
#2-. We obtain our genes results:
res_35vs05 <- topTags(qlf.2, n= nrow(qlf.2$table))
#3-. We transform our data into a data.frame:
res.df.2 <- as.data.frame(res_35vs05)
#4-. We get rid off those genes with NA values:
res.df.2 <- res.df.2[complete.cases(res.df.2),]
#5-. We order the results by
res.ord.2 <- res.df.2[order(res.df.2$FDR, decreasing = TRUE), ]
#6-. We save the results in a .csv file:
write.csv(file="P226_edgeR_Results_Leaf_AR_2.csv", x=res.ord.2)
#7-. We save the results in an excel file:
write.xlsx(res.ord.2, file="P226_edgeR_Results_Leaf_AR_2.xlsx", row.names = TRUE)
```

35% vs 1% analysis:

```r
#1-. We contrast the conditions we want to assess:
qlf.3 <- glmQLFTest(fit, contrast=c(-1,1,0,0))
#2-. We obtain our genes results:
res_35vs01 <- topTags(qlf.3, n= nrow(qlf.3$table))
#3-. We transform our data into a data.frame:
res.df.3 <- as.data.frame(res_35vs01)
#4-. We get rid off those genes with NA values:
res.df.3 <- res.df.3[complete.cases(res.df.3),]
#5-. We order the results by
res.ord.3 <- res.df.3[order(res.df.3$FDR, decreasing = TRUE), ]
# 6-. We save the results in a .csv file:
write.csv(file="P226_edgeR_Results_Leaf_AR_3.csv", x=res.ord.3)
#7-. We save the results in an excel file:
write.xlsx(res.ord.3, file="P226_edgeR_Results_Leaf_AR_3.xlsx", row.names = TRUE)
```

Data saving:

```r
#1-. We create an .RData object containing all data generated:
save.image(file= "P226_edgeR_Leaf_AR.RData")
```
