# Supplementary material for: Differential gene expression and gene ontologies associated with increasing water-stress in leaf and root transcriptomes of perennial ryegrass (Lolium perenne)
Source: PLoS One. 2019 Jul 30;14(7):e0220518. doi: 10.1371/journal.pone.0220518 (PMC6667212; doi:10.1371/journal.pone.0220518)
Supplement: S1 Results — (DOCX) [file pone.0220518.s016.docx]

**Supplementary Results S1**

**GO terms and associated gene models in Groups 5-8 (S3 Table) of the leaf transcriptome.**

Groups 5-8 consisted of enriched GO terms associated with a limited number of expression categories often detected by fewer of the analysis methods. Group 5 just contained BP GO terms, with child terms *pollen-pistil interaction* (GO:0009875), which was just detected by methods (S3 Table, Fig 2 and S4 Fig). Both of these were enriched in the *TC-ns_ns_down* but not in the equivalent AR expression category. Thus the differential expression levels for these gene models must have increased non-significantly during Early and Middle comparison stages before being down-regulated at the late comparison stage - significantly for the TC comparison but not the AR comparison (i.e. significantly compared to the level at 5% EWC but DESeq2 and *cellular homeostasis* (GO:0019725) which was detected by all of the analysis not at 35% EWC). The enriched *cellular homeostasis* GO term contained 20 gene models, the annotations for which indicated metal ion binding functions for 10 gene models and water transport and response to osmotic stress for 7 gene models – with some overlap between the two categories. The *pollen-pistil interaction* enriched GO term contained 9 gene models, all of which had receptor kinase annotations. Group 6 was associated with expression categories *AR-down_down_down, TC-down_ns_ns* and *AR-down_ns_down* with child terms of *signal transduction* (BP, GO:0007165) and *signalling receptor activity* (MF: GO:0038023) (S3 Table, Fig. 2 and S4 Fig.). GO terms enriched in *AR-down_down_down* and *AR-down_ns_down* were detected by a sub-set of the analysis methods, whereas the 2 MF GO terms in *TC-down_ns_ns* were detected by all of the analysis methods. Clearly *signal transduction* and *signalling receptor activity* are related GO terms and detection in *AR-down_down_down* and *TC-down_ns_ns* expression categories indicates that the contained gene models were down-regulated at the Early stage comparison and remained down-regulated at Middle and Late stage comparisons. The expression category *AR-down_ns_down* indicated the same trend though with slightly more variation. Enriched GO terms in Group 6 contained 29 gene models in total with annotations that suggested roles in plant hormone (abscisic acid, ethylene and cytokinin) and pathogenesis related signalling. Group 7 enriched GO terms were only detected by edgeR (*AR-ns_down_down*) and DESeq2 and edgeR (*TC-ns_down_up*) (S3 Table, Fig 2 and S4 Fig). All the enriched GO terms in *AR-ns_down_down* contained 54 gene models, 49 of which were associated with *nucleus* (CC, GO:005634) and *chromatin binding* (MF, G:0003682). Gene model annotations related to DNA replication and histone as well as transcription factors were represented. The enriched BP GO terms in Group 7 had *flower development* (GO:0009908) as the child term. In expression category *AR-ns_down_down,* *flower development* contained 11 gene models related to DNA replication and the cell-cycle, floral transition, male gametophyte development and the circadian rhythm, including the flowering gene *GIGANTEA*. For expression category *TC-ns_down_up*, *flower development* contained 9 gene models, three of which were annotated as the ‘canonical’ flowering induction genes, *Flowering Locus T*, *GIGANTEA* and *Hd1* (*CONSTANS*) as well as annotations associated with male meiosis, and the circadian rhythm. Group 8 consists of enriched GO terms which could not be assigned to any particular pattern of expression category and related GO hierarchy (S3 Table, Fig 2 and S4 Fig). It contained the 2 GO categories *response to external stimulus* (GO:0009605), detected by DESeq2 and *response to biotic stimulus* (GO:0009607) detected by DESeq2 and edgeR. These enriched GO terms contained gene models with annotations indicating a range of potential functions including cell division, light perception, photosynthesis and stress responses (S4A Table).
