# Supplementary material for: Differential gene expression and gene ontologies associated with increasing water-stress in leaf and root transcriptomes of perennial ryegrass (Lolium perenne)
Source: PLoS One. 2019 Jul 30;14(7):e0220518. doi: 10.1371/journal.pone.0220518 (PMC6667212; doi:10.1371/journal.pone.0220518)
Supplement: S2 Results — (DOCX) [file pone.0220518.s017.docx]

**Supplementary Results S2**

**Enriched GO terms identified when including the Late comparisons for the root transcriptome.**

Results S2 Table 1 and S5 Table illustrate the enriched GO terms identified when the non-replicated Late comparisons are included. As with the shoot data, the Late stage identified the greatest number of DEGs across all analysis methods, with a large increase on the overall numbers identified in Early and Middle comparison stages. While the certainty of the differential expression of individual gene models would need to be ascertained through repetition, the overall pattern of enriched GO terms is likely to be indicative of predominant biological processes within the roots at the Late comparison stage. The pattern of DEGs could be divided into 2 major groups (1 and 2) and 2 minor groups, largely associated with just DESeq2 detection (groups 3 and 4, which are not discussed further). Group 1 contained enriched GO terms identified predominantly in expression categories AR- and TC-*ns_ns_up* and *ns_ns_down* and Group 2 just AR- and TC-*ns_ns_down*. The child GO terms for Group 1 were *cellular homeostasis* (BP, GO:0019725), *plasma membrane* (CC, GO:0005886) and *DNA-binding transcription factor activity* (MF, GO:0003700). *Cellular homeostasis* was associated with 30 down-regulated gene models of which 11 were annotated as ‘aquaporins’ and 4 as ‘heavy metal-associated domain’ or ’metal tolerance’. Of the 40 up-regulated gene models, 4 were annotated as aquaporin,12 as ‘heavy metal-associated domain’, ‘metal tolerance’, ‘copper transport’, ‘ferritin’, or ‘vacuolar iron transporter’ and 2 ‘ABC transporter’ gene models which could be associated with heavy-metal transport in UniProt. For the *plasma membrane* category, while both down- and up-regulated categories had high proportions of annotations containing ‘kinase’ or ‘transporter’, 7 of the up-regulated, compared to 1 of the down-regulated gene models, were annotated as Casparian strip membrane proteins (CASP), suggesting transmembrane protein-mediated activity associated with cell-wall remodelling. *DNA-binding transcription factor activity* contained 46 down-regulated and 84 up-regulated gene models, including 6 down-regulated and 19 up-regulated gene-models with ‘WRKY’ annotations. Nine up-regulated gene models also contained the annotation ‘heat shock’ or ‘heat stress’, as compared to a single down-regulated gene model (S4C Table).

| **Results S2 Table 1.** Enriched GO terms associated with selected expression categories of root DEGs using Early, Middle and Late expression comparisons | | | | | | |
| --- | --- | --- | --- | --- | --- | --- |
| **Enriched GO term** | | | | | **Analysis method^2^** | |
| **Group^1^** | **i.d.** | **Category^3^** | **Position^4^** | **Description** | **AR-nnu^5^** | **AR-nnd^5^** |
| 1 | GO:0008152 | BP | 2 | metabolic process | J3,D | J1,J3,D,E,L |
|  | GO:0050896 | BP | 2 | response to stimulus | - | J1,J3,D,E,L |
|  | GO:0009719 | BP | 3 | response to endogenous stimulus | J3,D,E | - |
|  | GO:0006950 | BP | 3 | response to stress | - | J1,J3,D,E,L |
|  | GO:0080134 | BP | 5 | regulation of response to stress | - | - |
|  | GO:0031347 | BP | 6 | regulation of defense response | - | - |
|  | GO:0019748 | BP | 3 | secondary metabolic process | J1,J3,D,E,L | - |
|  | GO:0065008 | BP | 3 | regulation of biological quality | J1,J3,D,E,L | - |
|  | GO:0009058 | BP | 3 | biosynthetic process | J3,D | J1,D |
|  | GO:0009628 | BP | 3 | response to abiotic stimulus | J3,E | - |
|  | GO:0042592 | BP | 4 | homeostatic process | J1,J3,D,E,L | - |
|  | GO:0019725 | BP | 5 | cellular homeostasis | J1,J3,D,E,L | J3 |
|  | GO:0016020 | CC | 3 | membrane | J1,J3,D,E,L | J1,D |
|  | GO:0071944 | CC | 4 | cell periphery | J1,J3,D,E,L | J1,J3,D,E,L |
|  | GO:0005886 | CC | 5 | plasma membrane | J1,J3,D,E,L | J1,J3,D,E,L |
|  | GO:0005215 | MF | 2 | transporter activity | J1,J3,D,E,L | - |
|  | GO:0003677 | MF | 5 | DNA binding | J1,J3,D,E,L | J1,J3,D,E,L |
|  | GO:0140110 | MF | 6 | transcription regulator activity | J1,J3,D,E,L | J1,D |
|  | GO:0003700 | MF | 11 | DNA-binding transcription factor activity | J1,J3,D,E,L | J1,D |
| 2 | GO:0009987 | BP | 2 | cellular process | - | J1,J3,D,E,L |
|  | GO:0071840 | BP | 2 | cellular component organization or biogenesis | - | J1,J3,D,E,L |
|  | GO:0065007 | BP | 2 | biological regulation | - | J3,D,E,L |
|  | GO:0009056 | BP | 3 | catabolic process | - | J1,J3,D,E,L |
|  | GO:0071704 | BP | 3 | organic substance metabolic process | - | J1,J3,D,E,L |
|  | GO:0006807 | BP | 3 | nitrogen compound metabolic process | - | J1,J3,D,E,L |
|  | GO:0016043 | BP | 3 | cellular component organization | - | J1,J3,D,E,L |
|  | GO:0007049 | BP | 3 | cell cycle | - | J1,J3,D,E,L |
|  | GO:0065009 | BP | 3 | regulation of molecular function | - | J1,J3,D,E,L |
|  | GO:0023052 | BP | 3 | signaling | - | J3,E |
|  | GO:0050789 | BP | 3 | regulation of biological process | - | J3,E |
|  | GO:0005975 | BP | 4 | carbohydrate metabolic process | - | J1,J3,D,E,L |
|  | GO:0006091 | BP | 4 | generation of precursor metabolites and energy | - | J1,J3,D,E,L |
|  | GO:0044237 | BP | 4 | cellular metabolic process | - | J1,J3,D,E,L |
|  | GO:0044238 | BP | 4 | primary metabolic process | - | J1,J3,D,E,L |
|  | GO:1901564 | BP | 4 | organonitrogen compound metabolic process | - | J3,D,E,L |
|  | GO:0043170 | BP | 4 | macromolecule metabolic process | - | J1,J3,D,E,L |
|  | GO:0050790 | BP | 4 | regulation of catalytic activity | - | J1,J3,D,E,L |
|  | GO:0006725 | BP | 4 | cellular aromatic compound metabolic process | - | J1,D |
|  | GO:1901360 | BP | 4 | organic cyclic compound metabolic process | - | J1,D |
|  | GO:0046483 | BP | 4 | heterocycle metabolic process | - | J1,D |
|  | GO:0034641 | BP | 4 | cellular nitrogen compound metabolic process | - | J1,D |
|  | GO:0007154 | BP | 4 | cell communication | - | J3,D |
|  | GO:0019538 | BP | 5 | protein metabolic process | - | J3,D,E,L |
|  | GO:0043412 | BP | 5 | macromolecule modification | - | J1,J3,D,E,L |
|  | GO:0044260 | BP | 5 | cellular macromolecule metabolic process | - | J1,J3,D,E,L |
|  | GO:0009698 | BP | 5 | phenylpropanoid metabolic process | - | J1,J3,D,E,L |
|  | GO:0044550 | BP | 5 | secondary metabolite biosynthetic process | - | J1,J3,D,E,L |
|  | GO:0006139 | BP | 5 | nucleobase-containing compound metabolic process | - | J1,D |
|  | GO:0007165 | BP | 5 | signal transduction | - | J3 |
|  | GO:0036211 | BP | 6 | protein modification process | - | J1,J3,D,E,L |
|  | GO:0044267 | BP | 6 | cellular protein metabolic process | - | J1,J3,D,E,L |
|  | GO:0009699 | BP | 6 | phenylpropanoid biosynthetic process | - | J1,J3,D,E,L |
|  | GO:0090304 | BP | 6 | nucleic acid metabolic process | - | J1,D |
|  | GO:0006464 | BP | 7 | cellular protein modification process | - | J1,J3,D,E,L |
|  | GO:0006259 | BP | 7 | DNA metabolic process | - | J1,D,E,L |
|  | GO:0005623 | CC | 2 | cell | - | J1,J3,D,E,L |
|  | GO:0005576 | CC | 2 | extracellular region | - | J1,J3,D,E,L |
|  | GO:0044464 | CC | 3 | cell part | - | J1,J3,D,E,L |
|  | GO:0005622 | CC | 4 | intracellular | - | J1,J3,D,E,L |
|  | GO:0044424 | CC | 5 | intracellular part | - | J1,J3,D,E,L |
|  | GO:0043228 | CC | 5 | non-membrane-bounded organelle | - | J1,J3,D,E,L |
|  | GO:0030312 | CC | 5 | external encapsulating structure | - | J1,J3,D,E,L |
|  | GO:0005618 | CC | 6 | cell wall | - | J1,J3,D,E,L |
|  | GO:0005856 | CC | 7 | cytoskeleton | - | J1,J3,D,E,L |
|  | GO:0043232 | CC | 7 | intracellular non-membrane-bounded organelle | - | J1,J3,D,E,L |
|  | GO:0005634 | CC | 8 | nucleus | - | J1,D |
|  | GO:0003824 | MF | 2 | catalytic activity | - | J1,J3,D,E,L |
|  | GO:0005488 | MF | 2 | binding | - | J1,J3,D,E,L |
|  | GO:0060089 | MF | 2 | molecular transducer activity | - | J1,J3,D,E,L |
|  | GO:0030246 | MF | 3 | carbohydrate binding | - | J3,D,E,L |
|  | GO:0097159 | MF | 3 | organic cyclic compound binding | - | J1,J3,D,E,L |
|  | GO:1901363 | MF | 3 | heterocyclic compound binding | - | J1,J3,D,E,L |
|  | GO:0016740 | MF | 3 | transferase activity | - | J1,J3,D,E,L |
|  | GO:0016787 | MF | 3 | hydrolase activity | - | J1,J3,D,E,L |
|  | GO:0005515 | MF | 3 | protein binding | - | J1,J3,D,E,L |
|  | GO:0038023 | MF | 3 | signaling receptor activity | - | J1,J3,D,E,L |
|  | GO:0016772 | MF | 4 | transferase activity, transferring phosphorus-containing groups | - | J1,J3,D,E,L |
|  | GO:0036094 | MF | 4 | small molecule binding | - | J1,J3,D,E,L |
|  | GO:1901265 | MF | 4 | nucleoside phosphate binding | - | J1,J3,D,E,L |
|  | GO:0016817 | MF | 4 | hydrolase activity, acting on acid anhydrides | - | J1,J3,D,E,L |
|  | GO:0098772 | MF | 4 | molecular function regulator | - | J3,E,L |
|  | GO:0000166 | MF | 5 | nucleotide binding | - | J1,J3,D,E,L |
|  | GO:0016301 | MF | 5 | kinase activity | - | J1,J3,D,E,L |
|  | GO:0016818 | MF | 5 | hydrolase activity, acting on acid anhydrides, in phosphorus-containing anhydrides | - | J1,J3,D,E,L |
|  | GO:0097367 | MF | 5 | carbohydrate derivative binding | - | J1,D |
|  | GO:0030234 | MF | 5 | enzyme regulator activity | - | J3,E,L |
|  | GO:0016462 | MF | 6 | pyrophosphatase activity | - | J1,J3,D,E,L |
|  | GO:0042349 | MF | 6 | guiding stereospecific synthesis activity | - | J1,J3,D,E,L |
|  | GO:0017076 | MF | 6 | purine nucleotide binding | - | J1,D |
|  | GO:0032553 | MF | 6 | ribonucleotide binding | - | J1,D |
|  | GO:0043168 | MF | 6 | anion binding | - | J1,D |
|  | GO:0017111 | MF | 7 | nucleoside-triphosphatase activity | - | J1,J3,D,E,L |
|  | GO:0030554 | MF | 7 | adenyl nucleotide binding | - | J1,D |
|  | GO:0032555 | MF | 7 | purine ribonucleotide binding | - | J1,D |
|  | GO:0003774 | MF | 8 | motor activity | - | J1,J3,D,E,L |
|  | GO:0032559 | MF | 8 | adenyl ribonucleotide binding | - | J1,D |
|  | GO:0043531 | MF | 9 | ADP binding | - | J1,D |
| ^1^Only Groups 1 and 2 are shown. Details of Groups 3 and 4 are given in S4 Table ^2^Analysis methods identifying significant DEGs associated with the indicated enriched GO term and expression category. D = DESeq2, E = edgeR and L = limma-voom. ^3^MF = Molecular Function; BP = Biological Process; CC = Cellular component.  ^4^Ascending numbers indicate a more specific category in the GO terms hierarchies.  ^5^Abbreviated expression categories. u = up; d = down; n = ns. | | | | | | |

Group 2 consisted of 78 enriched GO terms indicating a general down-regulation of biological processes (e.g., *carbohydrate metabolic process* (GO:0005975), *generation of precursor metabolites and energy*, (GO:0006091) *regulation of catalytic activity* (GO:0050790), *phenylpropanoid metabolic process* (GO:0009698), *signal transduction* (GO:0007165), *cellular protein modification process* (GO:0006464), *DNA metabolic process* (GO:0006259)) across a number of cellular components (e.g., *extracellular region* (GO:0005576), *cell wall* (GO:0005618), *nucleus* (GO:0005634) and *cytoskeleton* (GO:0005856)) and encompassing a range of molecular functions (e.g., *protein binding* (GO:0005515), *carbohydrate binding* (GO:0030246), *nucleotide binding* (GO:0000166), *hydrolase activity* (GO:0016787), *signalling receptor activity* (GO:0038023), *kinase activity* (GO:0016301) and *transferase activity* (GO:0016740)). The child GO terms for the enriched GO terms which were generated by all of the analysis methods were *cellular protein modification process* (BP, GO:0006464), *cytoskeleton* (CC, GO:0005856)) and *intracellular non-membrane-bounded organelle* (CC, GO:0043232) and *motor activity* (MF, GO:0003774). *Cellular protein modification process* contained 313 gene models, of which 170 contained the annotation ‘kinase’, indicating a down-regulation of protein phosphorylation at the Late stage. A further 19 gene models were annotated as ‘ubiquitin’ or ‘U-box’, suggesting down-regulation of control mechanisms for protein turnover and metabolism. Gene model annotations associated with *cytoskeleton*, *intracellular non-membrane-bounded organelle* and *motor activity* had a preponderance of terms associated with the cytoskeleton; of the 212 gene models contained within these 3 enriched GO terms, 154 either contained ‘kinesin’, ‘actin’, ‘microtubule’ or ‘tubulin’, or could be associated with the cytoskeleton in Uniprot. Of these 154 cytoskeleton-associated gene models, kinesins were by far the largest class (92) – illustrating the diminishing of the capacity of the cell to continue with dynamic metabolic processes at the Late stage (S4C Table).
