# Supplementary material for: Differential gene expression and gene ontologies associated with increasing water-stress in leaf and root transcriptomes of perennial ryegrass (Lolium perenne)
Source: PLoS One. 2019 Jul 30;14(7):e0220518. doi: 10.1371/journal.pone.0220518 (PMC6667212; doi:10.1371/journal.pone.0220518)
Supplement: S1 Discussion — (DOCX) [file pone.0220518.s018.docx]

**Supplementary Discussion S1**

**AR and TC comparisons**

In this study, in addition to identifying DEGs against the initial, 35% EWC reference sampling point (AR), we also looked at the relative changes between consecutive sampling points (TC). While AR and TC are the same for the Early comparison – they can reflect different patterns of expression for the Middle and Late comparison points; AR comparisons have a stable reference point while TC comparisons have a dynamic reference point. For instance, AR-*ns_ns_down* and TC-*ns_ns_down* are not directly equivalent; of the 1154 gene models that were identified as DEGs in the J3 AR-*ns_ns_down* expression category, only 564 were identified as DEGs in any TC expression category – split across TC-*ns_ns_down* (485), TC-*ns_down_ns* (38), TC-*ns_up_down* (34) and TC-*ns_down_down* (7). Overall for the shoot transcriptome, TC comparisons identified slightly more DEGs in the Middle comparison stage than AR comparisons (c. 4% more averaged across methods) and substantially fewer DEGs in the Late comparison stage (c. 30% less averaged across methods). This latter observation may well reflect a more gradual rise or drop in gene expression over time for a subset of the genes which AR comparisons are more likely to detect than TC comparisons. For example, of the 2156 DEGs detected by DESeq2 in the leaf categories *AR-ns_ns_up* and *AR-ns_ns_down,* for 57% the direction of non-significant (*ns_ns*) differential expression in the Early and Middle stages was the same as the level that became significant at the Late stage (25% would be the random expectation). This rises to 78% if we just compare the direction of differential expression in the non-significant Middle stage with the significant Late stage (50% would be the random expectation). For the root transcriptomes, DESeq2 detected c. 4x more AR DEGs than TC comparisons at the Middle stage and all methods detected c. 1.5x more AR than TC DEGs at the Late stage – probably for the reasons as described above.

In addition to overall numbers of DEGs, there were quite marked differences in the detection of GO terms between AR- and TC- expression categories. Averaging across all the methods, of the total number of GO terms detected from the shoot comparisons, 67% were from the AR-comparisons and 33% from the TC comparisons. The root comparisons showed a similar trend. Why this should be is not immediately apparent. One possibility is that it is initial changes in gene expression levels from the ‘non-stressed’ situation (*i.e.* AR comparisons in this study) that are more relevant to responsive biological processes. Subsequent changes or fluctuations, to which TC comparisons will be more sensitive, may not be as co-ordinated in terms of biological process. Another possibility is that the majority of experiments which have looked at changes in gene expression levels use AR-type rather than TC-type comparisons and this may influence the derived association of annotation classifications and GO terms. Whatever the explanation, the AR comparisons generated a more complex description of the underlying processes, though specific TC expression categories may be useful when exploring possible gene actions. As discussed earlier, the non-overlapping sets of transcription factors identified in the leaf Group 2 AR-*up_ns_ns* and TC-*up_down_up* expression categories enriched within the GO term *DNA-binding transcription factor activity* (GO:0003700) may be an example of this.
